# Insights into the Evolution of Longevity from the Bowhead Whale Genome

**DOI:** 10.1016/j.celrep.2014.12.008

**Published:** 2015-01-06

**Authors:** Michael Keane, Jeremy Semeiks, Andrew E. Webb, Yang I. Li, Víctor Quesada, Thomas Craig, Lone Bruhn Madsen, Sipko van Dam, David Brawand, Patrícia I. Marques, Pawel Michalak, Lin Kang, Jong Bhak, Hyung-Soon Yim, Nick V. Grishin, Nynne Hjort Nielsen, Mads Peter Heide-Jørgensen, Elias M. Oziolor, Cole W. Matson, George M. Church, Gary W. Stuart, John C. Patton, J. Craig George, Robert Suydam, Knud Larsen, Carlos López-Otín, Mary J. O’Connell, John W. Bickham, Bo Thomsen, João Pedro de Magalhães

**Affiliations:** 1 Integrative Genomics of Ageing Group, Institute of Integrative Biology, University of Liverpool, Liverpool L69 7ZB, UK; 2 Howard Hughes Medical Institute and Departments of Biophysics and Biochemistry, University of Texas Southwestern Medical Center, Dallas, TX 75390-9050, USA; 3 Bioinformatics and Molecular Evolution Group, School of Biotechnology, Dublin City University, Glasnevin, Dublin 9, Ireland; 4 MRC Functional Genomics Unit, University of Oxford, Oxford OX1 3QX, UK; 5 Departamento de Bioquímica y Biología Molecular, Instituto Universitario de Oncología (IUOPA), Universidad de Oviedo, 33006 Oviedo, Spain; 6 Department of Molecular Biology and Genetics, Aarhus University, 8830 Tjele, Denmark; 7 Virginia Bioinformatics Institute, Virginia Tech, Blacksburg, VA 24061, USA; 8 Personal Genomics Institute, Genome Research Foundation, Suwon 443-270, Republic of Korea; 9 KIOST, Korea Institute of Ocean Science and Technology, Ansan 426–744, Republic of Korea; 10 Greenland Institute of Natural Resources, 3900 Nuuk, Greenland; 11 Department of Environmental Science, Center for Reservoir and Aquatic Systems Research (CRASR) and Institute for Biomedical Studies, Baylor University, Waco, TX 76798, USA; 12 Department of Genetics, Harvard Medical School, Boston, MA 02115, USA; 13 The Center for Genomic Advocacy (TCGA) and Department of Biology, Indiana State University, Terre Haute, IN 47809, USA; 14 Department of Forestry and Natural Resources, Purdue University, West Lafayette, IN 47907, USA; 15 North Slope Borough, Department of Wildlife Management, Barrow, AK 99723, USA; 16 Battelle Memorial Institute, Houston, TX 77079, USA; 17 Department of Wildlife and Fisheries Sciences, Texas A&M University, College Station, TX 77843, USA

## Abstract

The bowhead whale (*Balaena mysticetus*) is estimated to live over 200 years and is possibly the longest-living mammal. These animals should possess protective molecular adaptations relevant to age-related diseases, particularly cancer. Here, we report the sequencing and comparative analysis of the bowhead whale genome and two transcriptomes from different populations. Our analysis identifies genes under positive selection and bowhead-specific mutations in genes linked to cancer and aging. In addition, we identify gene gain and loss involving genes associated with DNA repair, cell-cycle regulation, cancer, and aging. Our results expand our understanding of the evolution of mammalian longevity and suggest possible players involved in adaptive genetic changes conferring cancer resistance. We also found potentially relevant changes in genes related to additional processes, including thermoregulation, sensory perception, dietary adaptations, and immune response. Our data are made available online (http://www.bowhead-whale.org) to facilitate research in this long-lived species.

## Highlights

• Genome and two transcriptomes of the bowhead whale, the longest-lived mammal
• Bowhead-specific mutations in genes associated with cancer and aging (e.g., ERCC1)
• Duplications in genes associated with DNA repair, cell cycle, and aging (e.g., PCNA)
• Changes in genes related to thermoregulation (UCP1) and other bowhead traits

## Graphical Abstract

**Figure:**
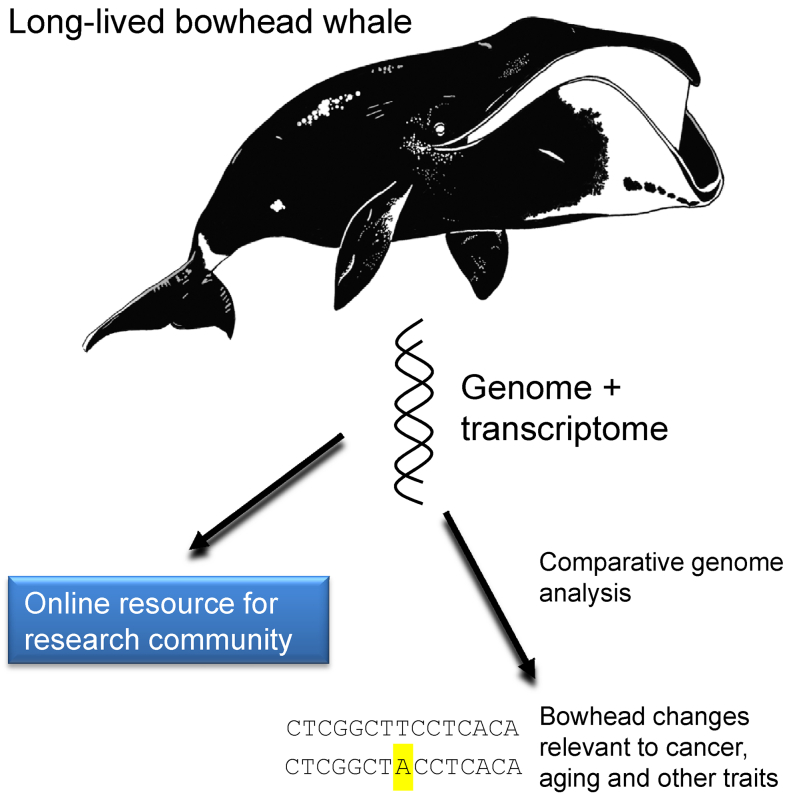

## Teaser

The bowhead whale is the longest-lived mammal, possibly living over 200 years. Keane et al. sequence the bowhead genome and transcriptome and perform a comparative analysis with other cetaceans and mammals. Changes in bowhead genes related to cell cycle, DNA repair, cancer, and aging suggest alterations that may be biologically relevant.

## Introduction

The lifespan of some animals, including quahogs, tortoises, and certain whale species, is far greater than that of humans (Austad, 2010; Finch, 1990). It is remarkable that a warm-blooded species such as the bowhead whale (*Balaena mysticetus*) has not only been estimated to live over 200 years (estimated age of one specimen 211 SE 35 years), suggesting it is the longest-lived mammal, but also exhibits very low disease incidence until an advanced age compared to humans (George et al., 1999; Philo et al., 1993). As in humans, the evolution of longevity in whales was accompanied by low fecundity and longer developmental time (Tacutu et al., 2013), as predicted by evolutionary theory. The cellular, molecular, and genetic mechanisms underlying longevity and resistance to age-related diseases in bowhead whales are unknown, but it is clear that, in order to live so long, these animals must possess preventative mechanisms against cancer, immunosenescence, and neurodegenerative, cardiovascular, and metabolic diseases. In the context of cancer, whales, and bowhead whales, in particular, must possess effective antitumor mechanisms. Indeed, given their large size (in extreme cases adult bowhead whales can weigh up to 100 tons and are therefore among the largest whales) and exceptional longevity, bowhead whale cells must have a significantly lower probability of neoplastic transformation relative to humans (Caulin and Maley, 2011; de Magãlhaes, 2013). Therefore, studying species such as bowhead whales that have greater natural longevity and resistance to age-related diseases than humans may lead to insights on the fundamental mechanisms of aging. Here, we report the sequencing and analysis of the genome of the bowhead whale, a species of the right whale family *Balaenidae* that lives in Arctic and sub-Arctic waters. This work provides clues regarding mechanisms underlying mammalian longevity and will be a valuable resource for researchers studying the evolution of longevity, disease resistance, and basic bowhead whale biology.

## Results

### Sequencing and Annotation of the Bowhead Whale Genome

We sequenced the nuclear genome of a female bowhead whale (*Balaena mysticetus*) using the Illumina HiSeq platform at ∼150× coverage. We followed established standards in the field in terms of sequencing paired-end libraries at high coverage plus mate-paired libraries of varying (3, 5, and 10 kb) insert sizes (Table 1). Contigs and scaffolds were assembled with ALLPATHS-LG (Gnerre et al., 2011). In line with other genomes sequenced with second-generation sequencing platforms, the contig N50 was 34.8 kb and scaffold N50 was 877 kb (Table 1); the longest scaffold in our assembly was 5,861 kb. In total, our assembly is ∼2.3 Gb long. Genome size was estimated experimentally to be 2.91 Gb in another female and 2.87 Gb averaged with one male (see Supplemental Results and Figure S1), but this discrepancy likely reflects highly repetitive regions, as observed for the genomes of other species with similar reported sizes such as the minke whale (Yim et al., 2014).

The full and partial completeness of the bowhead whale draft genome assembly was evaluated as 93.15% and 97.18%, respectively, by the CEGMA pipeline (Parra et al., 2007), which is comparable to the minke whale genome assembly (Yim et al., 2014). We also generated RNA sequencing (RNA-seq) data from seven adult bowhead whale tissues (cerebellum, kidney, muscle, heart, retina, liver, and testis) from specimens from Greenland and Alaska, resulting in two transcriptome assemblies (see Experimental Procedures) and annotated the genome using MAKER2, which combines ab initio methods, homology-based methods, and transcriptome data to derive gene models (Holt and Yandell, 2011). Our annotation contains 22,672 predicted protein-coding genes with an average length of 417 (median 307) amino acid residues. In addition, based on transcriptome data from two Alaskan individuals (Table S1), we estimated 0.5–0.6 SNPs per kilobase of RNA (Table S2). To begin annotation of the bowhead genome, we identified orthologs based on similarity with cow, human, and mouse genes/proteins (see Experimental Procedures), which allowed us to assign predicted gene symbols to 15,831 bowhead genes.

Moreover, to annotate microRNAs in the bowhead genome, we sequenced small RNA libraries prepared from kidney and skeletal muscle. The miRDeep algorithm (Friedländer et al., 2008, 2012) was used to integrate the sequencing data into a model of microRNA biogenesis by Dicer processing of predicted precursor hairpin structures in the genome, thus identifying 546 candidate microRNA genes. Of the 546 candidate miRNAs identified in the bowhead, 395 had seed sequences previously identified in miRNAs from human, cow, or mouse, whereas 151 did not. All of our data are available online from our Bowhead Whale Genome Resource portal (http://www.bowhead-whale.org).

### Analysis of the Draft Bowhead Whale Genome

Repeat sequences make up 41% of the bowhead genome assembly, most of which (78%) belong to the group of transposable elements (TEs). Although long interspersed nuclear elements (LINEs), such as L1, and short interspersed nuclear elements (SINEs) are widespread TEs in most mammalian lineages, the bowhead genome, similar to other cetacean genomes—minke, orca, and common bottlenose dolphin—is virtually devoid of SINEs (Supplemental Folder 1). LINE-1 (L1) is the most abundant TE, particularly in orca (90%) and minke whale (89%) (Figure S2). In comparison, TE diversity (measured with Shannon’s index) in the bowhead genome (0.947) is higher than in orca (0.469) and minke whale (0.515) but lower than in dolphin (1.389) and cow (Bovine Genome Sequencing and Analysis Consortium et al., 2009) (1.534).

As a first assessment of coding genes that could be responsible for bowhead whale adaptations, we used bowhead coding sequences to calculate pairwise dN/dS ratios for 9,682, 12,685, and 11,158 orthologous coding sequences from minke whale (*Balaenoptera acutorostrata*), cow (*Bos taurus*), and dolphin (*Tursiops truncatus*), respectively. It is interesting to note that there are high levels of sequence conservation in the protein coding regions between bowhead and these species: 96% (minke), 92% (dolphin), and 91% (cow). This is not surprising, however, given the long generation time of cetaceans and of the bowhead whale, in particular, with animals only reaching sexual maturity at >20 years (Tacutu et al., 2013).

Because the minke whale is the closest relative to the bowhead (divergence time 25–30 million years ago [Gatesy et al., 2013]) with a sequenced genome and is smaller (<10 tons) and probably much shorter lived (maximum lifespan ∼50 years) (Tacutu et al., 2013), comparisons between the bowhead and minke whale genomes may provide insights on the evolution of bowhead traits and of longevity, in particular. A number of aging- and cancer-associated genes were observed among the 420 predicted bowhead-minke orthologs with dN/dS exceeding 1, including *suppressor of cytokine signaling 2* (*SOCS2*), *aprataxin* (*APTX*), *noggin* (*NOG*), and *leptin* (*LEP*). In addition, the top 5% genes with high dN/dS values for bowhead-minke relative to the values for minke-cow and minke-dolphin orthologs included *forkhead box O3* (*FOXO3*), *excision repair cross-complementing rodent repair deficiency*, *complementation group 3* (*ERCC3*), and *fibroblast growth factor receptor 1* (*FGFR1*). The data on dN/dS ratios are also available on our portal to allow researchers to do their own analysis and quickly retrieve gene(s) of interest.

**Figure 1:**
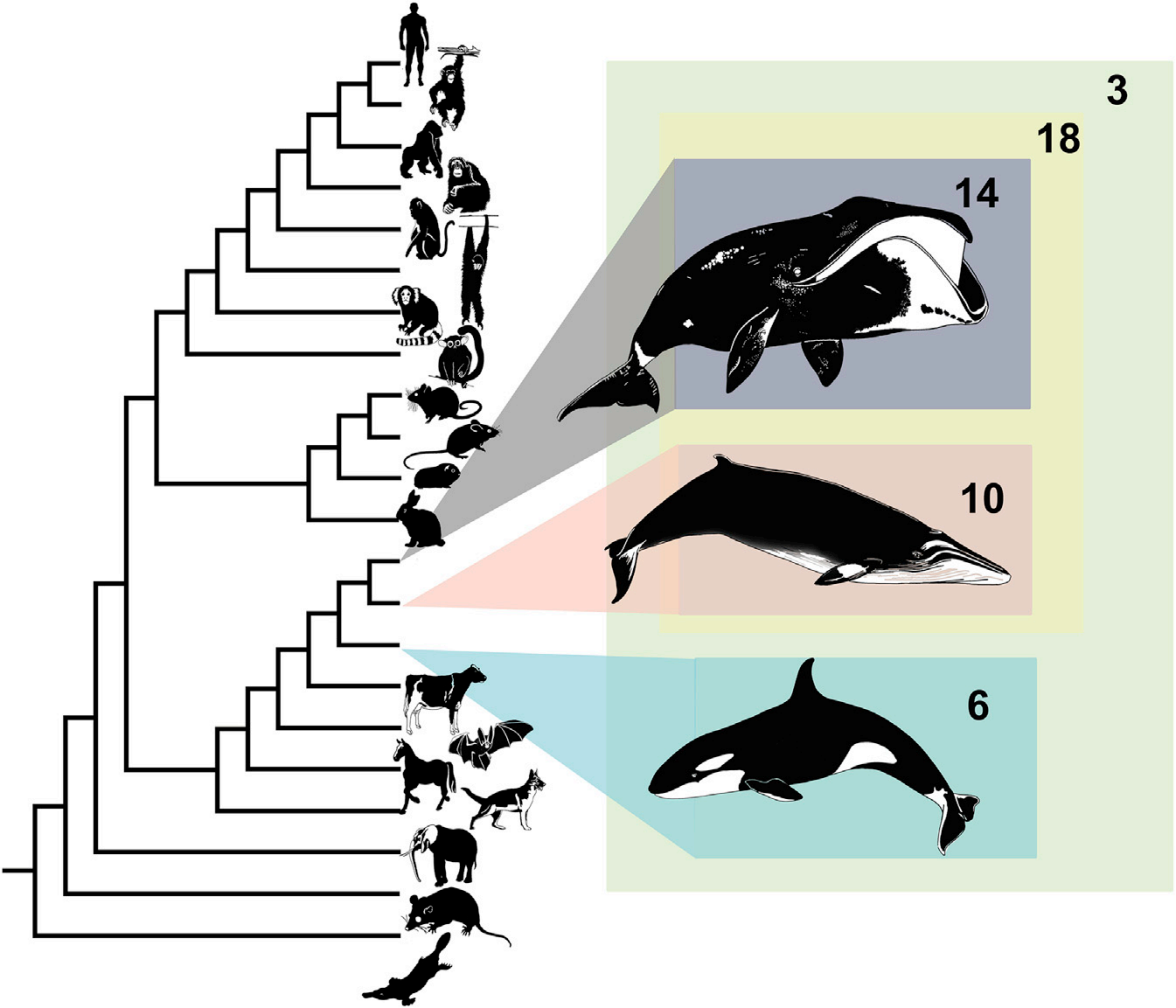
Phylogeny of Mammals Used in Codon-Based Maximum Likelihood Comparison of Selective Pressure Variation The number of candidate genes under positive selection on each lineage is indicated.

In a complementary and more detailed analysis of selective pressure variation, we used codon-based models of evolution (Yang, 2007) to identify candidate genes with evidence of lineage-specific positive selection (see Experimental Procedures). Using bowhead, minke, and orca protein-coding data along with a variety of available high-quality completed genomes from Laurasiatheria, Euarchontoglires, marsupial, and monotreme species, we identified a total of 866 single-gene ortholog families (SGOs) (i.e., these gene families have no more than one copy in each species). We tested each of the extant whale lineages, the ancestral baleen whale, and the most recent common ancestor (MRCA) of bowhead, minke, and orca, a total of five lineages (Figure 1), for evidence of lineage-specific positive selection.

Of the two extant whales analyzed, the number of SGOs exhibiting signatures of lineage-specific positive selection were as follows: bowhead (15 gene families) and minke (ten gene families). The small number of candidates under positive selection likely reflects the high level of protein conservation between bowhead and other cetaceans as well as the stringent filtering of candidates due to data-quality concerns; all results and alignments are provided in Supplemental Folder 1. A few genes associated with disease were identified, including *BMP and activin membrane-bound inhibitor* (*BAMBI*), which has been associated with various pathologies, including cancer, and also poorly studied genes of potential interest like *GRB2-binding adaptor protein, transmembrane* (*GAPT*).

**Figure 2:**
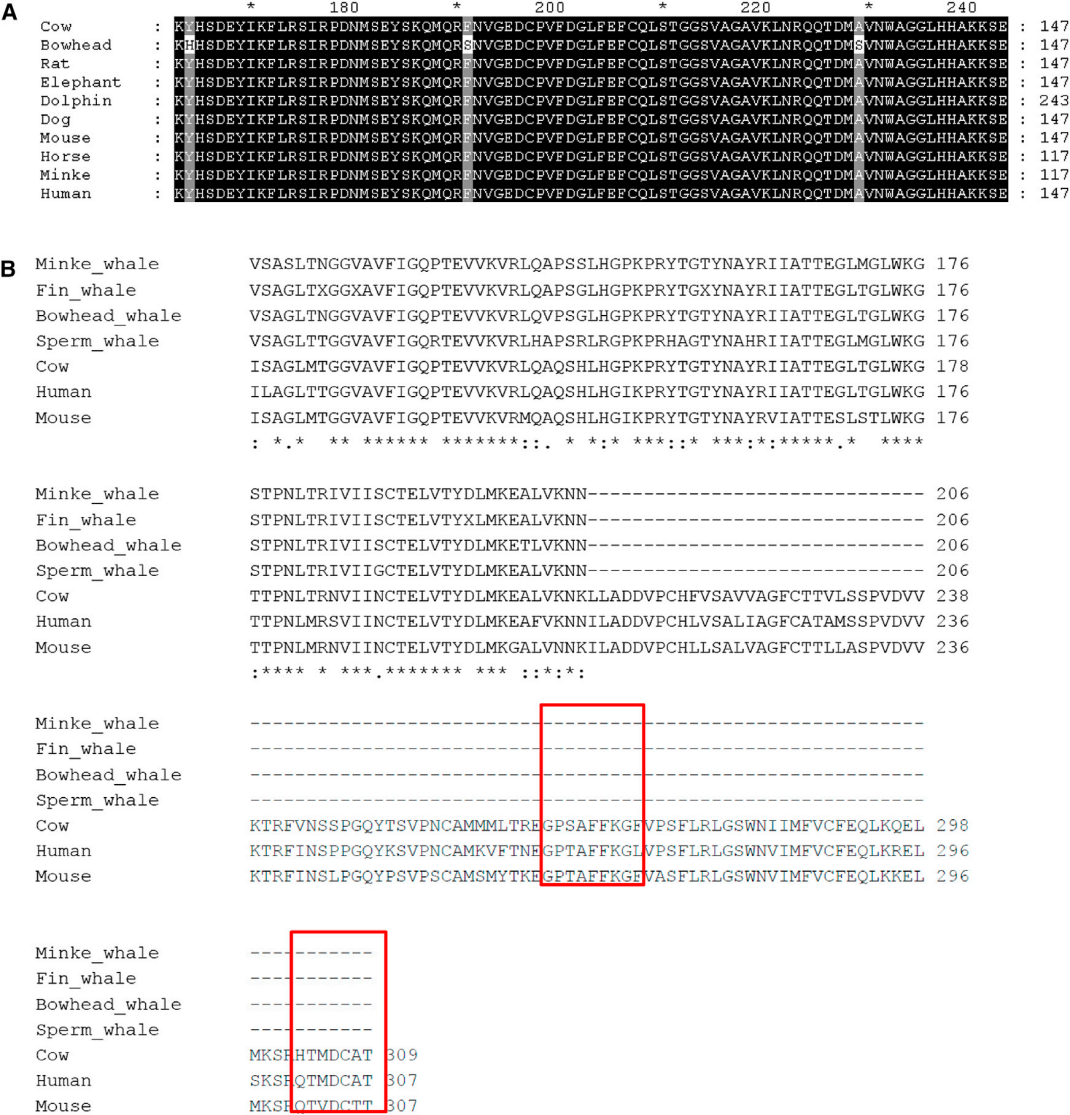
Multiple Protein Sequence Alignments of HDAC2 and UCP1 (A) Partial alignment of bowhead HDAC2 with mammalian orthologs. Unique bowhead residues are highlighted at human positions 68, 95, and 133. (B) Partial alignment of whale UCP1 with mammalian orthologs. Conserved regions involved in UCP1 are marked in red.

In addition to the codon-based models of evolution, we wished to identify bowhead whale specific amino acid replacement substitutions. To this end, we aligned orthologous sequences between the bowhead whale and nine other mammals—a total of 4,358 alignments (see Experimental Procedures). Lineage-specific residues identified in this way have previously been shown to be indicative of significant changes in protein function (Tian et al., 2013). Our analysis revealed several proteins associated with aging and cancer among the top 5% of unique bowhead residues by concentration (i.e., normalized by protein length), including ERCC1 (excision repair cross-complementing rodent repair deficiency, complementation group 1), HDAC1 (histone deacetylase 1), and HDAC2 (Figure 2A). ERCC1 is a member of the nucleotide excision repair pathway (Gillet and Schärer, 2006), and disruption results in greatly reduced lifespan in mice and accelerated aging (Weeda et al., 1997). Histone deacetylases play an important role in the regulation of chromatin structure and transcription (Lee et al., 1993) and have been associated with longevity in *Drosophila* (Rogina et al., 2002). As such, these represent candidates involved in adaptive genetic changes conferring disease resistance in the bowhead whale. The full results are available in Supplemental Folder 1.

In addition to genes related to longevity, several interesting candidate genes emerged from our analysis of lineage-specific residues of potential relevance to other bowhead traits. Of note, a number of proteins related to sensory perception of sound were also identified with bowhead-specific mutations, including otoraplin (OTOR) and cholinergic receptor, nicotinic, alpha 10 (CHRNA10), which could be relevant in the context of the bowhead’s ability to produce high- and low-frequency tones simultaneously (Tervo et al., 2011). In addition, many proteins must play roles in the large differences in size and development between the bowhead and related species and our results reveal possible candidates for further functional studies; for example, in the top ten proteins, SNX3 (sorting nexin 3) has been associated in one patient with eye formation defects and microcephaly (Vervoort et al., 2002), and WDR5 (WD repeat-containing protein 5) has been associated with osteoblast differentiation and bone development (Gori et al., 2006).

In the naked mole rat, a poikilotherm with a low metabolic rate and body temperature when compared to other mammals, unique changes in uncoupling protein 1 (UCP1), which is used to generate heat, have been previously found (Kim et al., 2011). Because the specific metabolic power output of cells in vivo for large whales must be much less than for smaller mammals (West et al., 2002), it is interesting to note that UCP1 of whales has a premature stop codon in C-terminal region, which is functionally important and conserved in other mammals (Figure 2B). It is tempting to speculate that these changes are related to differences in thermoregulation between whales and smaller mammals.

### Potential Gene Duplications and Gene Losses

Gene duplication is a major mechanism through which phenotypic innovations can evolve (Holland et al., 1994; Kaessmann, 2010). Examples of mammalian phenotypic innovations associated to gene duplication include duplication of *RNASE1*, a pancreatic ribonuclease gene, in leaf-eating monkeys that contributed to adaptative changes in diet and digestive physiology (Zhang et al., 2002), a duplication of *GLUD1* in hominoids that subsequently acquired brain-specific functions (Burki and Kaessmann, 2004), and domestication of two syncytin gene copies that contributed to the emergence of placental development in mammals (Dupressoir et al., 2009). We surveyed the bowhead whale genome for expanded gene families that may reflect lineage-specific phenotypic adaptations and traits.

**Figure 3:**
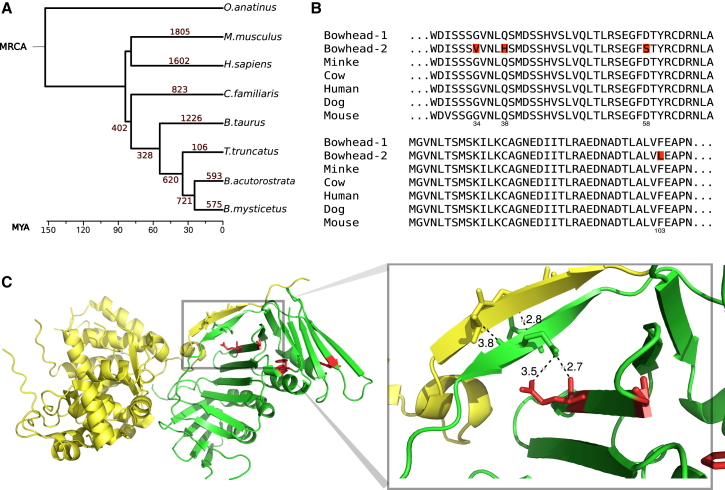
Gene Family Expansion and PCNA (A) Gene family expansion. Numbers in red correspond to the predicted number of gene expansion events during mammalian evolution. Mean divergence time estimates were used from TimeTree (Hedges et al., 2006) for scaling. (B) Multiple sequence alignment of PCNA residues 28–107, showing bowhead whale-specific duplication (gene IDs: bmy 16007 and bmy 21945). Lineage-specic amino acids in the duplicated PCNA of bowhead whales are highlighted in red. (C) Crystal structure of the PCNA (green) and FEN-1 (yellow) complex. Lineage-specific residues on the PCNA structure are colored in red. A zoom in on the structures reveals a putative interaction between two β sheets, one within PCNA and another within FEN-1. This interaction may be altered through a second interaction between the PCNA β sheet and a lineage-specic change from glutamine to histidine within PCNA. Distance measurements between pairs of atoms are marked in black. PDB accession number: 1UL1. See also Table S3 and Figure S3.

In the bowhead whale lineage, 575 gene families were predicted to have expanded (Figure 3). However, because gene expansion predictions are susceptible to false-positives owing to pseudogenes and annotation artifacts among other biases, we applied a stringent filter based on percentage of identity (Experimental Procedures) that reduced the number of candidate expansions to 41 (see Supplemental Folder 1 for the complete list). A functional enrichment analysis of these gene families, using default parameters in DAVID (Huang et al., 2009), only revealed a statistically significant enrichment (after correction for multiple hypothesis testing; Bonferroni <0.001) for genes associated with translation/ribosome. Given the association between translation and aging, for instance, in the context of loss of proteostasis (López-Otín et al., 2013), it is possible that these results reflect relevant adaptations in the bowhead whale.

Upon manual inspection of the gene expansion results, we found several duplicates of note. For instance, *proliferating cell nuclear antigen* (*PCNA*) is duplicated in bowhead whales with one copy harboring four lineage-specific residue changes (Figure 3B). Based on our RNA-seq data mapped to the genome (see Experimental Procedures and full results in Supplemental Folder 1), both *PCNA* copies are expressed in bowhead whale muscle, kidney, retina, and testis. By mapping the lineage-specific residues onto the structure of PCNA in complex with FEN-1, we uncovered one amino acid substitution (Q38H), which may affect the interaction between PCNA and FEN-1 (Figure 3C). A subsequent branch-site test for selective pressure variation (see Experimental Procedures and Table S3) revealed that one substitution, D58S, may have undergone positive selection in the bowhead-whale lineage (with a posterior probability score of 0.983). The duplication of *PCNA* during bowhead-whale evolution is of particular interest due to its involvement in DNA damage repair (Hoege et al., 2002) and association with aging in that its levels in aged rat liver seem to relate to the decrease in the rate of cell proliferation (Tanno et al., 1996).

Another notable duplicated gene is *late endosomal/lysosomal adaptor, MAPK and MTOR activator 1 (LAMTOR1)*, in which six bowhead-specific amino acid changes were identified (Figure S3). LAMTOR1 is involved in amino acid sensing and activation of mTORC1, a gene strongly associated with aging and cancer (Cornu et al., 2013). The original *LAMTOR1* copy was expressed in all bowhead whale adult tissues for which we have data, with the duplicate having much lower (but detectable) expression in heart and retina. Also of note, putative duplications of *26S proteasome non-ATPase regulatory subunit 4 (PSMD4)* and *ubiquitin carboxyl-terminal esterase L3* (*UCHL3*) were identified with evidence of expression, which is intriguing considering the known involvement of the proteasome-ubiquitin system in aging (López-Otín et al., 2013) and given previous evidence that this system is under selection specific to lineages where longevity increased (Li and de Magalhães, 2013); *UCHL3* has also been involved in neurodegeneration (Kurihara et al., 2001). Other gene duplications of potential interest for their role in mitosis, cancer, and stress response include *cAMP-regulated phosphoprotein 19* (*ARPP19*), which has three copies even though we only detected expression of two copies, *stomatin-like 2* (*STOML2*), *heat shock factor binding protein 1* (*HSBP1*) with four copies of which two appear to be expressed, *spermine synthase (SMS)* and *suppression of tumorigenicity 13* (*ST13*).

Similar to previous genome characterizations, we chose the complete set of known protease genes for a detailed supervised analysis of gene loss (Quesada et al., 2009). This procedure highlighted multiple gene loss events potentially related to the evolution of several cetacean traits, including adaptations affecting the immune system, blood homeostasis, digestive system, and dentition (Figure S4). Thus, the cysteine protease CASP12, a modulator of the activity of inflammatory caspases, has at least one conserved premature stop codon in bowhead and minke whales. Interestingly, whereas this protease is conserved and functional in almost all of the terrestrial mammals, most human populations display different deleterious variants (Fischer et al., 2002), presumably with the same functional consequences as the premature stop codons in whales. Likewise, two paralogues of carboxypeptidase A (CPA2 and CPA3) have been pseudogenized in bowhead and minke whales. Notably, CPA variants have been associated with increased risk for prostate cancer in humans (Ross et al., 2009), which could be of interest in the context of reduced cancer susceptibility in whales compared with humans (de Magalhães, 2013).

Additionally, we found that multiple coagulation factors have been lost in bowhead and minke whales. The finding of bowhead whale-specific changes is also noteworthy because it could be related to the special characteristics of this mammal. For example, OTUD6A, a cysteine protease with a putative role in the innate immune system (Kayagaki et al., 2007), is specifically lacking in the assembled genome and expressed sequences of the bowhead whale. In addition, whereas the enamel metalloprotease MMP20 has been lost in bowhead and minke whales (Yim et al., 2014), our analysis suggests that these genomic events happened independently (see alignments in Supplemental Folder 1). Finally, as aforementioned, the cysteine protease UCHL3 seems to have been duplicated through a retrotranscription-mediated event in a common ancestor to bowhead and minke whales, although only the genome of the bowhead whale shows a complete, putatively functional open reading frame for this extra copy of the gene. UCHL3 may play a role in adipogenesis (van Beekum et al., 2012), which indicates that this duplication might be related to the adaptation of the bowhead whale to the challenging arctic environment. These results suggest specific scenarios for the role of proteolysis in the evolution of *Mysticetes*. Specifically, given the relationship between immunity and aging (López-Otín et al., 2013), some of these findings might open new approaches for the study of this outstanding cetacean.

## Discussion

The genetic and molecular mechanisms by which longevity evolves remain largely unexplained. Given the declining costs of DNA sequencing, de novo genome sequencing is rapidly becoming affordable. The sequencing of genomes of long-lived species allows comparative genomics to be employed to study the evolution of longevity and has already provided candidate genes for further functional studies (de Magalhães and Keane, 2013). Nonetheless, deciphering the genetic basis of species differences in longevity has major intrinsic challenges (de Magalhães and Keane, 2013), and much work remains to uncover the underlying mechanisms by which some species live much longer than others. In this context, studying a species so long lived and with such an extraordinary resistance to age-related diseases as the bowhead whale will help elucidate mechanisms and genes conferring longevity and disease resistance in mammals. Remarkably, large whales with over 1,000 times more cells than humans do not exhibit an increased cancer risk (Caulin and Maley, 2011), suggesting the existence of natural mechanisms that can suppress cancer more effectively in these animals. Having the genome sequence of the bowhead whale will allow researchers to study basic molecular processes and identify maintenance mechanisms that help preserve life, avoid entropy, and repair molecular damage. When compared to transcriptome data (Seim et al., 2014), the genome’s greater completeness and quality permits additional (e.g., gene loss and duplication) and more thorough analyses. Besides, whereas the genomes of many commercially important agricultural species have been reported, the bowhead genome sequence is the first for a species key to a subsistence diet of indigenous communities. One of the outputs of this project will be to facilitate and drive research in this long-lived species. Data and results from this project are thus made freely available to the scientific community on an online portal (http://www.bowhead-whale.org/). We provide this key resource for studying the bowhead whale and its various traits, including its exceptional longevity and resistance to diseases.

## Experimental Procedures

### DNA and RNA Sampling in Greenland

Bowhead (*Balaena mysticetus*) DNA used for genome sequencing was isolated from muscle tissue sampled from a 51-year-old female (ID no. 325) caught in the Disko Bay, West Greenland in 2009 (Heide-Jørgensen et al., 2012). Tissue samples were stored at –20°C immediately after collection. Age estimation was performed using the aspartic acid racemization technique (Garde et al., 2007). CITES no. 12GL1003387 was used for transfer of biological material. Bowhead RNA used for RNA-seq and small RNA analysis was isolated from two different individuals: kidney samples were from a 44-year-old female (ID no. 500) and muscle samples were isolated from a 44-year-old male (ID no. 322). For more details of the individual whales, see Heide-Jørgensen et al. (2012).

### Genome Sequencing

DNA was extracted following standard protocols, quantified using Qubit and run on an agarose gel to ensure no degradation had occurred. We then generated ∼150× coverage of the genome using the Illumina HiSeq 2000 platform with 100 bp reads, sequencing paired-end libraries, and mate-paired libraries with insert sizes of 3, 5, and 10 kb (Table 1). Sequencing was performed at the Liverpool Centre for Genomic Research (CGR; http://www.liv.ac.uk/genomic-research/).

### Genome Assembly

Libraries were preprocessed in-house by the CGR to remove adaptor sequences. The raw fastq files were trimmed for the presence of the Illumina adaptor sequence using Cutadapt and then subjected to window-based quality trimming using Sickle with a minimum window quality score of 20. A minimum read-length filter of 10 bp was also applied. Libraries were then assembled with ALLPATHS-LG (Gnerre et al., 2011), which performed all assembly steps including read error correction, initial read alignment, and scaffolding. ALLPATHS-LG build 43762 was used with the default input parameters, including K = 96. Several build parameters were automatically determined by the software at run time per its standard algorithm. Of 2.88 × 10^9^ paired fragment reads and 1.87 × 10^9^ paired jumping reads, 0.015% were removed as poly(A) and 1.5% were removed due to low-frequency kmers; 54% of jumping read pairs were error-corrected, and overall 33% of jumping pairs were redundant. In total, we used 216 Gbp for the 2.3 Gb assembly, meaning that coverage retained for the assembly was ∼95×. Full assembly and read usage data are shown in Supplemental Folder 2. Assembly completeness was assayed with CEGMA by searching for 248 core eukaryotic genes (Parra et al., 2007).

### Genome Size Determination

To determine the genome size for bowhead whale, spleen tissues were acquired from one male (10B17) and one female (10B18). Both whales were harvested in 2010 as part of the native subsistence hunt in Barrow, Alaska. Sample processing and staining followed the methods of Vindeløv and Christensen (1994). Instrument description and additional methodological details are provided in Oziolor et al. (2014). Briefly, flow cytometric genome size determination is based on propidium iodide fluorescent staining of nuclear DNA. Mean fluorescence is calculated for cells in the G0 and G1 phases of the cell cycle. This method requires direct comparison to known standards to convert measured fluorescence to pg of DNA. The primary standard used in this study was the domestic chicken (*Gallus gallus domesticus*). Chicken red blood cells are widely used as a genome size standard, with an accepted genome size of C = 1.25 pg. Chicken whole blood was purchased from Innovative Research. Mouse (*Mus musculus*) and rat (*Rattus norvegicus*) were included as internal checks, with estimates for both falling within 3% of previously published genome size estimates (Vinogradov, 1998). Spleen tissues from three male 129/SvEvTac laboratory mice and a single male Harlan SD Sprague-Dawley laboratory rat were used.

### Transcriptome Sequencing and Assembly: Greenland Samples

Total RNA was extracted from the kidney and muscle employing the mirVanaTM RNA extraction kit (Ambion). RNA integrity of the individual RNA samples was assessed on a 1% agarose gel using an Agilent 2100 Bioanalyzer (Agilent Technologies). Library preparation was performed using the ScriptSeqTM mRNA-seq library preparation kit from Epicenter according to the manufacturer’s protocol (Epicenter) and sequenced (100 bp paired end) as multiplexed samples using the Illumina HiSeq 2000 analyzer. Fastq generation and demultiplexing were performed using the CASAVA 1.8.2 package (Illumina). The fastq files were filtered for adapters, quality, and length using Trimmomatic (v.0.27), with a window size of 4, a base quality cutoff of 20, and a minimum length of 60 (Lohse et al., 2012). De novo transcriptome assembly was performed using the short read assembler software Trinity (release 2013-02-25), which is based on the de Bruijn graph method for assembly, with default settings (Grabherr et al., 2011).

### Transcriptome Sequencing and Assembly: Alaskan Samples

Tissue biopsies were obtained from two male bowhead whales harvested by Inupiat hunters at Barrow, Alaska during the Fall hunt of 2010; heart, cerebellum, liver, and testes were biopsied from male bowhead number 10B16, and retina from male bowhead 10B20. Samples were immediately placed in liquid nitrogen and transported in a dry shipper to Purdue University. RNA was extracted using TRIZOL reagent (Invitrogen) following the manufacturer’s protocol. RNA was purified using an Invitrogen PureLink Micro-to-Midi columns from the Total RNA Purification System using the standard protocol. RNA quantity and quality was estimated with a spectrophotometer (Nanodrop) and by gel electrophoresis using an Agilent model 2100 Bioanalyzer. cDNA libraries were constructed by random priming of chemically sheared poly A captured RNA. Randomly primed DNA products were blunt ended. Products from 450–650 bp were then isolated using a PippenPrep. After the addition of an adenine to the fragments, a Y primer amplification was used to produce properly tailed products. Paired-end sequences of 100 bp per end were generated using the Illumina HiScan platform. Sequences with primer concatamers, weak signal, and/or poly A/T tails were culled. The Trinity software package for de novo assembly (Grabherr et al., 2011) was used for transcript reconstruction (Table S1).

### Small RNA Sequencing and Annotation

To annotate microRNA genes in the bowhead genome, we conducted deep sequencing of two small RNA libraries prepared from muscle and kidney tissues (Greenland samples). Total RNA was isolated using mirVana miRNA Isolation Kit (Ambion). Small RNA in the 15-40 nucleotides range was gel purified and small RNA libraries were prepared for next-generation sequencing using the ScriptMiner Small RNA-Seq Library Preparation Kit (Epicenter). The two libraries were sequenced on an Illumina Hi-Seq 2000 instrument to generate single end sequences of 50 nucleotides. Primary data analysis was done using the Illumina CASAVA Pipeline software v.1.8.2, and the sequence reads were further processed by trimming for adapters and filtering for low quality using Trimmomatic (Lohse et al., 2012). Identification of conserved and novel candidate microRNA genes in the bowhead genome was accomplished by applying the miRDeep2 algorithm (Friedländer et al., 2008, 2012).

### Evaluation of Repeat Elements

To evaluate the percentage of repeat elements, RepeatMasker (v.4.0.3; http://www.repeatmasker.org/) was used to identify repeat elements, with parameters set as “-s -species mammal.” RMBlast was used as a sequence search engine to list out all types of repeats. Percentage of repeat elements was calculated as the total number of repeat region divided by the total length of the genome, excluding the N-region. Genomes of minke whale (*Balaenoptera acutorostrata*), orca (*Orcinus orca*), common bottlenose dolphin (*Tursiops truncates*), and cow (*Bos taurus*) were downloaded from NCBI and run in parallel for comparison with the bowhead genome.

### Genome Annotation

Putative genes were located in the assembly by structural annotation with MAKER2 (Holt and Yandell, 2011), which combined both bowhead transcriptomes with comparative and de novo prediction methods including BLASTX, Exonerate, SNAP, Genemark, and Augustus. In addition to the RNA-seq data, the entire SwissProt database and the draft proteome of dolphin were used as input to the comparative methods. Repetitive elements were found with RepeatMasker (http://www.repeatmasker.org/). The complete set of MAKER input parameters, including training sets used for the de novo prediction methods, are listed in Supplemental Folder 2. In total, 22,672 protein-coding genes were predicted with an average length of 417 (median 307) amino acid residues.

The RNA-seq data from seven adult bowhead tissues described above were then mapped to the genome: FastQC (http://www.bioinformatics.babraham.ac.uk/projects/fastqc/) was used for quality control to make sure that data of all seven samples was of acceptable quality. STAR (Dobin et al., 2013) was used to generate genome files from the bowhead assembly and to map the reads to the bowhead genome with 70.3% of reads mapping, which is in line with other results including those in the minke whale (Yim et al., 2014). To count the reads overlapping genes, we used ReadCounter (van Dam et al., 2015). The results obtained from all seven samples were combined into a single file describing the number of nonambiguously mapping reads for each gene (full results in Supplemental Folder 1). Of the 22,672 predicted protein-coding genes, 89.5% had at least ten reads mapping and 97.5% of predicted genes had at least one read mapping to them, which is again comparable to other genomes like the minke whale genome (Yim et al., 2014).

To allow the identification of orthologous relationships with bowhead proteins, all cow protein sequences were downloaded from Ensembl (Flicek et al., 2013). Cow was initially used because it is the closest relative to the bowhead with a high-quality annotated genome available. First, BLASTP (10^−5^) was used to find the best hit in the cow proteome for every predicted bowhead protein, and then the reciprocal best hit for each cow protein was defined as an ortholog. In addition, human and mouse orthologs from the OPTIC pipeline (see below) were used to assign predicted gene symbols to genes and proteins. A total of 15,831 bowhead genes have a putative gene symbol based on these predictions. Homologs in minke whale and dolphin were also derived and are available on our bowhead genome portal.

### Genome Portal

To facilitate further studies of these animals, we constructed an online genome portal: The Bowhead Whale Genome Resource (http://www.bowhead-whale.org/). Its database structure, interface, and functionality were adapted from our existing Naked Mole Rat Genome Resource (Keane et al., 2014). Our data and results are available from the portal, and supplemental methods and data files are also available on GitHub (https://github.com/maglab/bowhead-whale-supplementary).

### Pairwise dN/dS Analysis

The CodeML program from the PAML package was used to calculate pairwise dN/dS ratios (Yang, 2007). This is done using the ratio of nonsynonymous substitutions per nonsynonymous site (dN) to synonymous substitutions per synonymous site (dS), dN/dS, or ω (Yang, 2007). Specifically, these pairwise dN/dS ratios were calculated for bowhead coding sequences and orthologous sequences from minke, cow, and dolphin, excluding coding sequences that were less than 50% of the length of the orthologous sequence. The results were then ranked by decreasing dN/dS and are available on our bowhead genome portal. In addition, the ratio of the bowhead-minke dN/dS value to the higher of the dN/dS values for minke-cow and minke-dolphin was calculated to identify genes that evolved more rapidly on the bowhead lineage.

### Assessment of Selective Pressure Variation across Single-Gene Orthologous Families Using Codon-Based Models of Evolution

To accurately assess variation in selective pressure on the bowhead, minke, and orca lineages in comparison to extant terrestrial mammals, we created a protein-coding database spanning the placental mammals. Along with the orca (http://www.ncbi.nlm.nih.gov/bioproject/189949), minke (Yim et al., 2014), and bowhead data described above, we extracted protein coding sequences from Ensembl Biomart v.73 (Flicek et al., 2013) for the following 18 genomes: chimpanzee, cow, dog, elephant, gibbon (5.6× coverage), gorilla, guinea pig, horse, human, macaque, marmoset, microbat, mouse, opossum, orangutan, platypus, rabbit, and rat. These genomes were all high coverage (mostly >6× coverage) with the exception of gibbon (Supplemental Folder 2). Sequence similarity searches were performed using mpi-BLAST (v 1.6.0) (Altschul et al., 1990) (http://www.mpiblast.org/) on all proteins using a threshold of 10^−7^. Gene families were identified using in-house software that clusters genes based on reciprocal BLAST hits (Altschul et al., 1990). We identified a total of 6,630 gene families from which we extracted the single-gene orthologous families (SGOs). Families were considered SGOs if we identified a single-gene representative in each species (one-to-one orthologs), and to account for lower coverage genomes and missing data we also considered cases where a specific gene was not present in a species, i.e., one-to-zero orthology. SGOs were only considered for subsequent analysis if they contained more than seven species in total and if they contained no internal stop codons (indicative of sequencing errors). In total, we retained 866 SGOs for further analysis. Multiple sequence alignments (MSAs) were generated using default parameters in PRANK (v.100802) (Löytynoja and Goldman, 2008). To minimize potential false-positives due to poor sequence quality, the MSAs of the 866 SGOs underwent strict data-quality filtering. The first filter prohibited the presence of gaps in the MSA if created by unique insertions (>12 bp) in either bowhead or minke sequences. The second filter required unaligned bowhead or minke sequences to be at least half the length of their respective MSA. These two filters refined the number of testable SGOs to 319. The gene phylogeny of each SGO was inferred from the species phylogeny (Morgan et al., 2013). CodeML from the PAML software package (v.4.4e) (Yang, 2007) was employed for our selective pressure variation analyses. We analyzed each of the 319 refined SGOs using the nested codon-based models of evolution under a maximum likelihood framework. We employed the likelihood ratio test (LRT) using nested models of sequence evolution to evaluate a variety of models of codon sequence evolution (Yang, 2007). In general, these codon models allow for variable dN/dS ratios (referred to as ω throughout) among sites in the alignment, along different lineages on our phylogenetic tree, or a combination of both variations across lineages and sites. To assess the significance of fit of each model to the data, we used the recommended LRTs in CodeML (Yang, 2007) for comparing nested models (see Supplemental Folder 2). The LRT test statistic approximates the chi-square (χ2) distribution critical value with degrees of freedom equal to the number of additional free parameters in the alternative model. The goal of the codon-based modeling is to determine the selective pressures at work in a lineage and site-specific manner.

The models applied follow the standard nomenclature (i.e., model M1, M2, A, and A null) (Yang, 2007). Model M1 assumes that there are two classes of sites—those with an ω value of zero and those with an ω value of 1. Model M2 allows for three classes of sites—one with an ω value of zero, one with an ω value of one and one with an ω value that is not fixed to any value. Given the relationship between M1 and M2, they can be tested for the significance of the difference of the fit of these two models using an LRT with df = 2. Finally, we used model A that allows the ω value to vary across sites and across different lineages in combination. With model A, we can estimate the proportion of sites and the dN/dS ratio in the foreground lineage of interest in comparison to the background lineages and the estimated dN/dS ratio is free to vary above 1 (i.e., positive selection). Model A can be compared with its site-specific counterpart (model M1) using the LRT with df = 2. In addition, the lineage and site-specific model model A null was applied as a second LRT with model A. In model A null, the additional site category is fixed at neutral rather than being estimated from the data, and this LRT provides an additional test for model A (Zhang et al., 2005). In this way, we performed independent tests on each of the extant cetacean lineages (orca, minke, and bowhead), as well as testing each ancestral cetacean branch (the MRCA of the two baleen whales and the MRCA of all three cetaceans), to determine if there were signatures of positive selection that are unique to each lineage (Yang and dos Reis, 2011). Using empirical Bayesian estimations, we identified the specific residues that are positively selected in each lineage tested. Positive selection was inferred if all of the following criteria were met: (1) if the LRT was significant, (2) if the parameters estimated under that model were concurrent with positive selection, and (3) if the alignment in that region was of high quality (as judged by alignment completeness and quality in that region). The posterior probability (PP) of a positively selected site is estimated using two calculations: Naive Empirical Bayes (NEB) or Bayes Empirical Bayes (BEB) (Yang, 2007). If both NEB and BEB are predicted, we reported the BEB results as they have been shown to be more robust under certain conditions (Yang et al., 2005). For all models used in the analysis where ω is estimated from the data, a variety of starting ω values was used for the calculation of likelihood estimates. This ensures that the global minimum is reached.

### Identification of Proteins with Bowhead-Unique Residues

An in-house Perl pipeline was used to align each bowhead protein with orthologs from nine other mammals: human (*Homo sapiens*), dog (*Canis familiaris*), mouse (*Mus musculus*), rat (*Rattus norvegicus*), minke whale (*Balaenoptera acutorostrata*), cow (*Bos taurus*), dolphin (*Tursiops truncatus*), horse (*Equus caballus*), and elephant (*Loxodonta africana*) and then identify the unique bowhead amino acid residues. Gaps were excluded from the analysis, and a maximum of one unknown residue was allowed in species other than the bowhead. The results were ranked by the number of unique residues normalized by the protein length (full results in Supplemental Folder 1).

### Gene Expansion Analysis, Filtering, and Expression

Human, mouse, dog, cow, dolphin, and platypus genomes and gene annotations were obtained from Ensembl (Flicek et al., 2013), the genome and gene annotation of minke whale were obtained from Yim et al. (2014). In total, 21,069, 22,275, 19,292, 19,988, 15,769, 17,936, 20,496, and 22,733 human, mouse, dog, cow, dolphin, platypus, minke whale, and bowhead whale genes, respectively, were used to construct orthology mappings using OPTIC (Heger and Ponting, 2007). Briefly, OPTIC builds phylogenetic trees for gene families by first assigning orthology relationships based on pairwise orthologs computed using PhyOP (Goodstadt and Ponting, 2006). Then, a tree-based method, PhyOP, is used to cluster genes into orthologous groups, and, last, gene members are aligned and phylogenetic trees built with TreeBeST (Vilella et al., 2009). Further details are available in the OPTIC paper (Heger and Ponting, 2007). Predicted orthology groups can be accessed at http://genserv.anat.ox.ac.uk/clades/vertebrates_bowhead.

To identify gene families that underwent expansion, gene trees were reconciled with the consensus species tree, and duplicated nodes were identified. The tree used, derived from TimeTree (Hedges et al., 2006), was: (mm_oanatinus5, ((mm_cfamiliaris3, (mm_btaurus, (mm_ttruncatus, (mm_balaenoptera, mm_bmysticetus)))), (mm_hsapiens10, mm_mmusculus5))). The following algorithm was used to reconcile gene and species trees.

A stringent filter was applied to the data so that gene duplicates in bowhead whales were required to differ by at most 10% in protein sequence from a cognate copy but were also required to differ by at least 1% to avoid assembly artifacts and to remove recently duplicated copies with no function. Further manual inspection of the alignments was performed. Gene expression inferred from our RNA-seq data was used to check the expression of duplicates.

An in-house peptide-sensitive approach was used to align the PCNA cDNA into codons, and CodeML/PAML was used to test M0, a one-rate model that assumes the same rate of evolution in all branches against M2^∧^a, a branch site test with one rate for the background and one rate for the bowhead whale branch (Yang, 2007).

## Author Contributions

G.M.C., J.C.G., R.S., J.W.B., B.T., and J.P.M. conceived and designed the study; L.B.M., E.M.O., and C.W.M. performed the experiments; M.K., J.S., A.E.W., Y.I.L., V.Q., L.B.M., S.v.D., D.B., P.I.M., P.M., L.K., J.B., H.-S.Y., G.W.S., J.C.P., C.L.-O., M.J.O., J.W.C., and J.P.M. analyzed the data; T.C., N.V.G., N.H.N., M.P.H.-J., R.S., and K.L. contributed reagents/materials/analysis tools; and M.K. and J.P.M. wrote the paper.

## Figures and Tables

**Table 1:** Statistics of the Bowhead Whale Genome Sequencing

Sequence Data Generated		
Libraries	Total Data (Gb)	Sequence Coverage (for 2.91 Gb)
200 bp paired-end	149.1	51.2×
500 bp paired-end	141.7	48.7×
3 kb mate-paired	57.3	19.7×
5 kb mate-paired	72.5	24.9×
10 kb mate-paired	28.5	9.8×
**Total**	449.1	154.3×

**Genome Assembly Statistics**			
**Assembly**	**N50 (kb)**	**Number**	**Total Size (Gb)**
Contigs	34.8	113,673	2.1
Scaffolds	877	7,227	2.3

See also Figures S1 and S2.

## Accession Numbers

Our data and results can be downloaded from the Bowhead Whale Genome Resource (http://www.bowhead-whale.org/downloads/). In addition, data are available at the NCBI BioProject PRJNA194091 with raw sequencing reads in the Sequence Read Archive (SRP050351).

## Supplemental Information

Document S1. Supplemental Results, Figures S1–S4, and Tables S1–S3

Supplemental Folder 1. Full List of Transposable Elements Distribution, Full List of Filtered and Unfiltered Gene Duplications, Gene Loss Alignments, Full List of Candidates under Selection, Selective Pressure Variation QC Alignments, and Full List of Bowhead-Specific Mutations, Related to Results

Supplemental Folder 2. Species Used in the Analysis of Selective Pressure Variation, Single-Gene Orthologous Families Tested, Likelihood Ratio Tests, MAKER Parameters, and Full Assembly and Read Usage Data, Related to Experimental Procedures

Document S2. Article plus Supplemental Information
