# Supplementary material for: Insights into the Evolution of Longevity from the Bowhead Whale Genome
Source: Cell Rep. 2015 Jan 6;10(1):112–22. doi: 10.1016/j.celrep.2014.12.008 (PMC4536333; doi:10.1016/j.celrep.2014.12.008)
Supplement: Document S1. Supplemental Results, Figures S1–S4, and Tables S1–S3 [file mmc1.pdf]

Cell Reports, Volume *10*

Supplemental Information

Insights into the Evolution of Longevity from the Bowhead Whale Genome

Michael Keane, Jeremy Semeiks, Andrew E. Webb, Yang I. Li, Víctor Quesada, Thomas Craig, Lone Bruhn Madsen, Sipko van Dam, David Brawand, Patrícia I. Marques, Pawel Michalak, Lin Kang, Jong Bhak, Hyung-Soon Yim, Nick V. Grishin, Nynne Hjort Nielsen, Mads Peter Heide-Jørgensen, Elias M. Oziolor, Cole W. Matson, George M. Church, Gary W. Stuart, John C. Patton, J. Craig George, Robert Suydam, Knud Larsen, Carlos López-Otín, Mary J. O'Connell, John W. Bickham, Bo Thomsen, and João Pedro de Magalhães

Supplemental Data

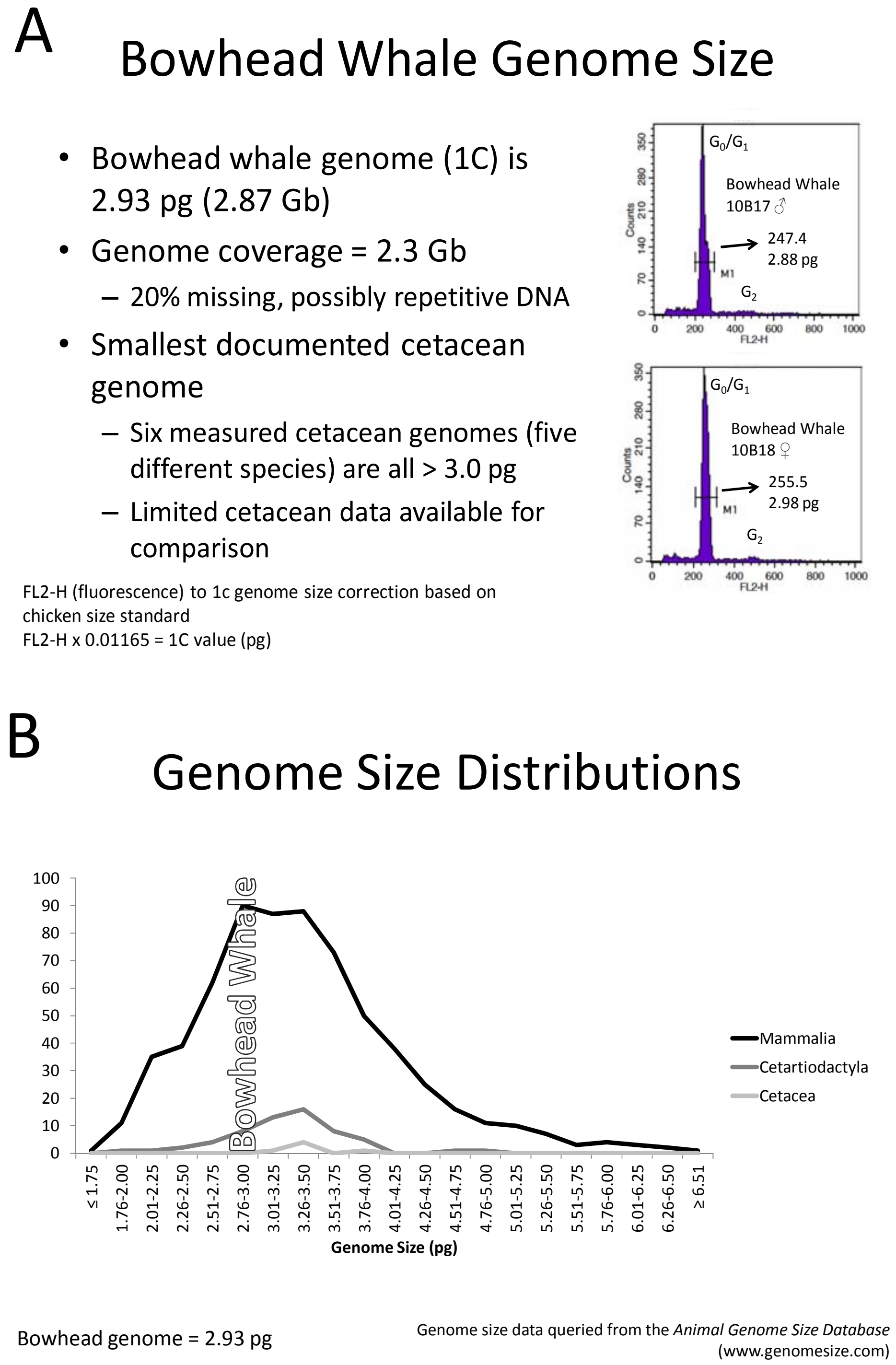

Figure S1: Bowhead whale genome size, Related to Table 1. S1A—DNA flow histograms (right two panels) of a male and female bowhead whale showing an approximately 3% difference

in estimated genome sizes. The mean estimated genome size is C = 2.93 pg. S1B—Distribution of genome sizes of Mammalia, Cetartiodactyla, and Cetacea. Bowhead whales have an estimated genome size (2.93 pg) well below the mammalian mean (3.5 pg). This is the first species of baleen whale to be reported and has the lowest C-value of any cetacean. Some cetartiodactyls have lower genome sizes but most are higher than bowheads. Related to Results.

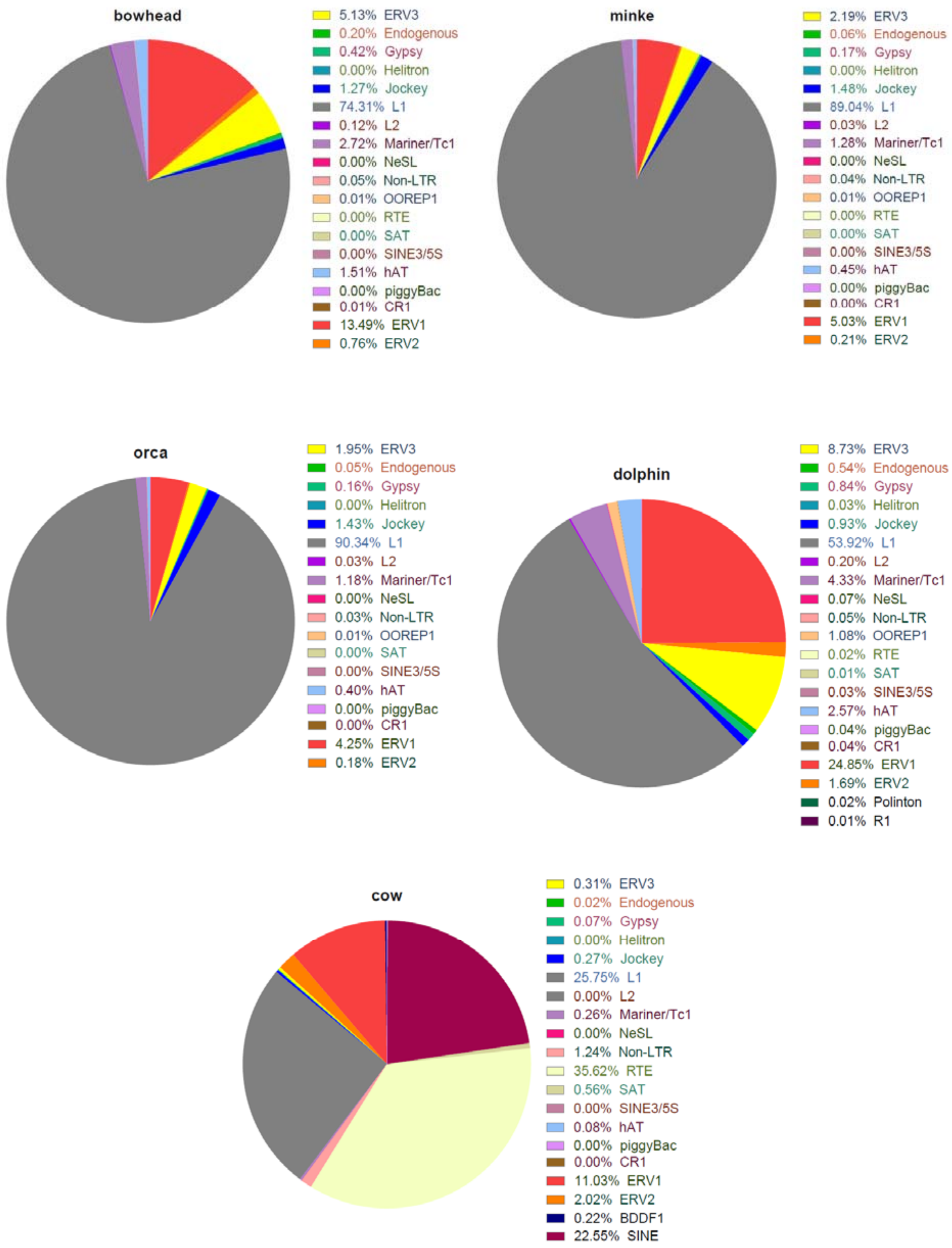

Figure S2: Repeat sequences, Related to Table 1. Transposable elements in bowhead whale and related species.

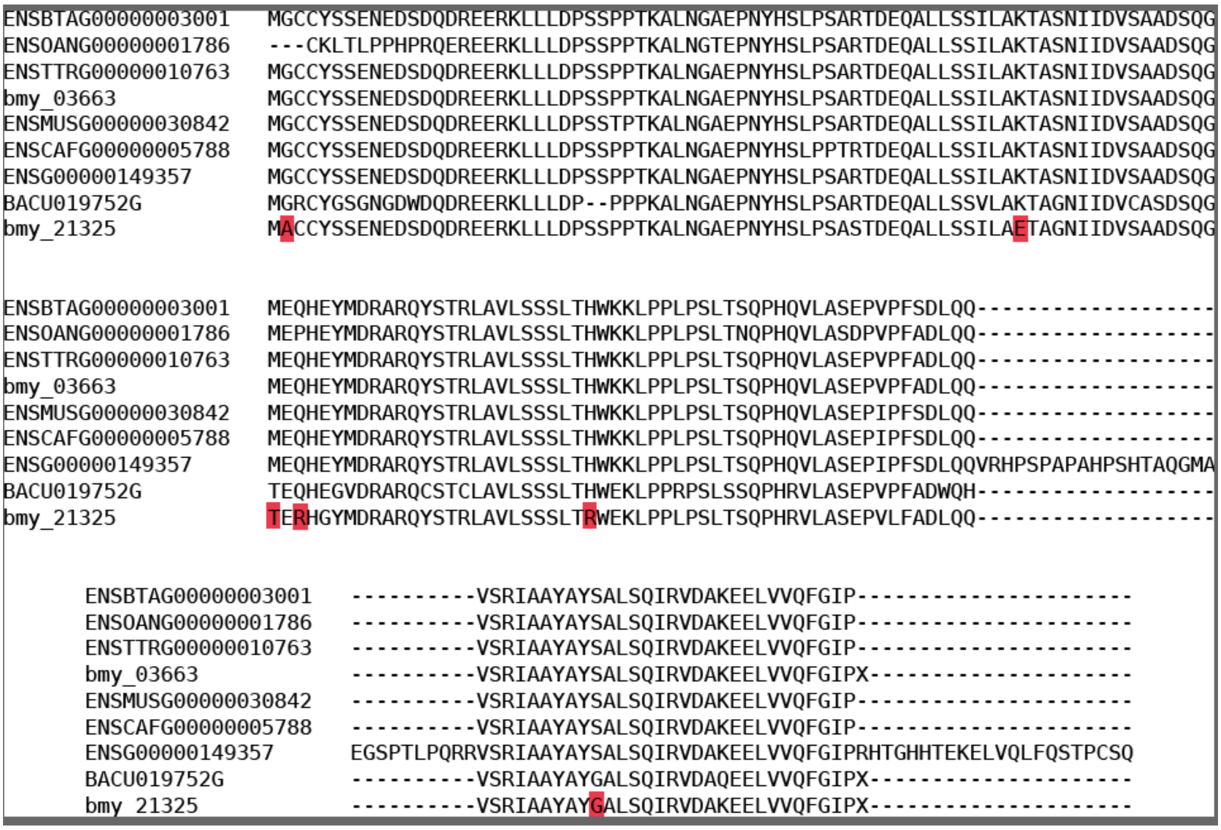

Figure S3: Putative LAMTOR1 gene duplication in the bowhead, Related to Figure 3.

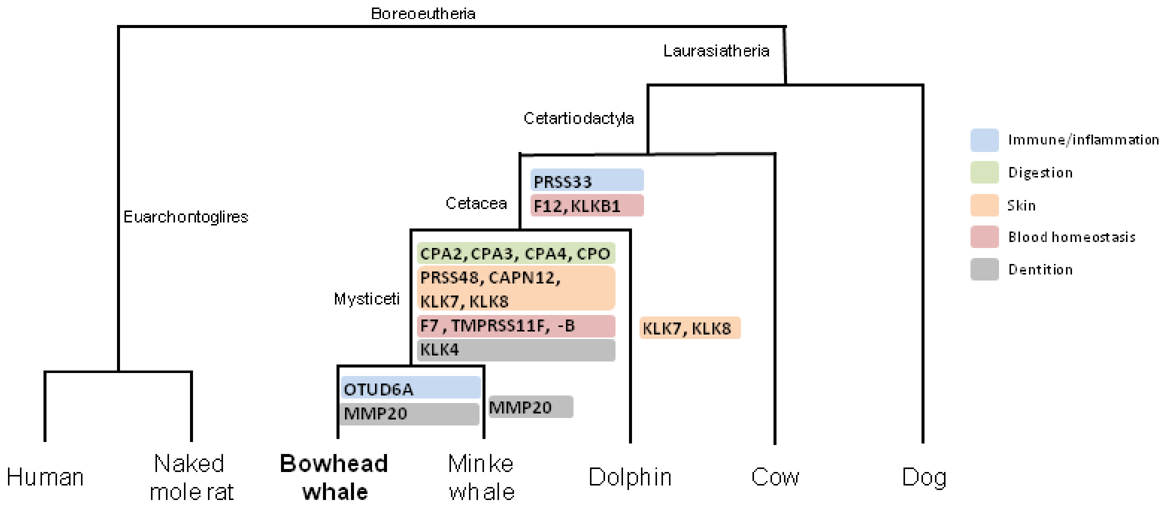

Figure S4: Genomic losses in the bowhead whale degradome, Related to Results. Each gene is depicted on the right side of the branch where each loss is inferred. Putative roles of each protease are shown in different colours.

Table S1: RNA sequencing of 5 tissues from two bowhead whales, Related to Results and Experimental Procedures. All Reads refers to all sequenced fragments of any size, Large Contigs includes contigs comprised of multiple reads of 500 bp or larger, and All Contigs refers to small and large contigs combined.

**All Reads**	**Total reads**	138,495,774
	**Total bases**	13,162,565,851
	**Size range of reads**	2-101
	**N50 (modal size)**	101
	**Average length**	95
**Large Contigs**	**Contig size**	≥500
	**Total large contigs**	157,699
	**Total number of bases**	322,342,312
	**Contig size range**	500-24765
	**N50 (modal size)**	3,442
	**Average length**	2,044
**All Contigs**	**Total number of contigs**	423,657
	**Total number of bases**	401,340,157
	**Contig size range**	201-24765
	**N50 (modal size)**	2,436
	**Average length**	947
**Annotations**	**Number of annotated contigs**	81,319

Table S2: SNP frequencies estimated for each tissue per size class of contigs, Related to Results. Tissues 1-4 are from bowhead 10B16 and retina is from 10B20.

Contig Size (bp)	Tissue					
	1. Cerebellum	2. Heart	3. Liver	4. Testes	5. Retina	Tissues 1-4
≥201	2.7E-04	2.7E-04	2.7E-04	2.8E-04	3.1E-04	3.9E-04
>500	3.3E-04	3.2E-04	3.2E-04	3.4E-04	3.8E-04	4.8E-04
>1000	3.6E-04	3.5E-04	3.6E-04	3.8E-04	4.2E-04	5.2E-04
>2000	3.9E-04	3.8E-04	3.8E-04	4.1E-04	4.5E-04	5.6E-04
>3000	4.0E-04	3.9E-04	3.9E-04	4.2E-04	4.6E-04	5.7E-04
>4000	4.2E-04	4.0E-04	4.0E-04	4.4E-04	4.7E-04	5.9E-04
>5000	4.3E-04	4.0E-04	4.0E-04	4.5E-04	4.7E-04	6.0E-04
>6000	4.5E-04	4.2E-04	4.2E-04	4.7E-04	4.9E-04	6.2E-04

Table S3: Branch-site test Bayes empirical Bayes values for putative positively selected sites in PCNA, Related to Figure 3. *Indicates statistical significance.

Site	Sub.	BEB
34	V	0.774
38	H	0.753
58	S	0.983*
103	L	0.758
231	T	0.748

Supplemental Results

Genome size estimation

Simple ratios, assuming a chicken genome size of C = 1.25 pg, were used to convert mean fluorescence to pg of DNA. Mouse and rat tissues, which were included as an additional confirmation of genome size estimation accuracy, were within 2% and 3%, respectively, of published values (data not shown). Bowhead whale genome sizes were estimated using both chicken as a size standard, and by averaging the estimates produced from all three size standards (chicken, mouse, and rat) independently. The results from these two methods yielded estimates of 2.93 and 2.92 pg, respectively. Of particular interest was the variability in individual bowhead whale genome size estimates, an approximately 3% difference between our two samples (Figure S1A). While not known during sample processing and initial analysis, bowhead #10B17, the individual with the smaller genome (2.88 pg), was a male, whereas bowhead #10B18, the individual with the larger genome (2.98 pg) was a female. This difference in genome size is entirely accounted for by the expected differences in masses of X and Y chromosomes. As is customary, the final bowhead whale genome size estimate was calculated as the average of the male and female genome sizes, 2.93 pg or 2.87 Gb (Figure S1A).

This is the first cytometric-based estimate of genome size for a baleen whale. The value C = 2.93 pg is the lowest value yet for a cetacean (Figure S1B) and is on the low end of values for Cetartiodactyla (artiodactyls and cetaceans). The average of all mammals is C = 3.5 pg, so bowheads are low for mammals. Most of the mammalian species with lower genome sizes are animal with small body size and high metabolic rates including bats, shrews and some rodents. Only toothed whales are available for comparison and thus it is not known if bowheads are atypical for baleen whales. Nevertheless it is apparent from these results that bowheads are at the low end of the scale for mammals in general.

There are two possible explanations for the relatively small genome of the bowhead whale. The first is that it could be a plesiomorphic character unchanged during the evolution and diversification of cetartiodactyls. This is possible given the fact that low genome sizes are also found in suids, camelids, giraffids, cervids and bovids, notwithstanding the fact that most cetartiodactyls have higher values (http://www.genomesize.com/) and the ancestral character state is not known.

The second possible explanation is that the low genome size of the bowhead is a derived, adaptive, character state that has evolved as a result of nucleotypic effects. A correlate to small genome size is not obvious but could be related to metabolic rate or gas exchange in this highly specialized diving mammal.

Significance of the genome size estimate of bowheads also relates to its genome sequence. There is a discrepancy in the genome size as measured in base pairs (one picogram = 978 megabases) with flow cytometry compared to the total sequence length in the genome sequence (Figure S1A). The flow cytometric method is 20% higher than the sequence total and this is likely due to the inability of the bioinformatics methods to assemble repetitive DNA sequences. So, the estimated genome size gives us an independent estimate of the amount of sequence not represented in the assembled genome sequence.

Additional studies of genome size are needed for baleen whales in order to determine if the bowhead is an outlier or if this group of mammals has an unexpectedly small genome size. In this way perhaps the adaptive correlates, if any exist, can be determined. In addition, it is anticipated that other baleen whales will be the subjects of genome sequences and a better understanding of the amount of DNA sequence not assembled is useful for determining the overall percent coverage of the genome sequence.

RNA sequencing in Alaskan specimens

Sequence analysis of RNA from 5 tissues representing two bowhead whales produced a total of 138,495,774 sequence reads comprising >13 billion bp after quality control and primer trimming. The numbers and sizes of reads and contigs are reported in Table S1. The total number of annotated contigs was 81,319. The estimated number of bowhead contigs identified as being homologous to human genes was approximately 14,000 or ca. 60% of the known human genes.

Table S2 shows the estimated frequencies of SNPs among the 5 tissues sampled. The two individuals sampled can be compared by reference to retina (bowhead 10B20) and Tissues 1-4 (bowhead 10B16). The data are shown for 8 size classes of contigs. As contigs size increases, the frequency of estimated SNPs increases. With this method, there appears to be approximately 0.5-0.6 SNPs per 1,000 bases of RNA.

Analysis of bowhead whale protease genes

Proteases form a diverse group of enzymes that share the ability to hydrolyze peptide bonds. The biological and pathological significance of this enzymatic activity has prompted the definition of the degradome as the complete repertoire of proteases in an organism[1]. From a genomic point of view, the degradome is highly attractive for several reasons. First, it is composed of a large number of genes. Thus, the human degradome includes about 600 protease genes, which represents almost 3% of the total annotated human protein-coding genes. Moreover, catalytic domains of proteases exhibit a high sequence diversity, which is further increased by the frequent attachment of auxiliary, non-proteolytic domains to the catalytic moieties[2]. Some of the protease genes have been shown to occur in genomic clusters, which is convenient for the study of short-term evolution. By contrast, most protease genes are randomly distributed throughout the annotated genomes. Therefore, the degradome forms a representative subset of the coding

genome of a species. Notably, this structural diversity also reflects the multiple biological roles of proteases in every organism. Thus, beyond their obvious role in protein digestion, proteases also mediate regulatory processes through their ability to perform highly specific reactions of proteolytic processing, which have contributed to the acquisition of different functional capacities during evolution.

The comparison of the degradomes of the bowhead whale to those of minke whale, human and other mammals shows multiple events of gene loss in cetaceans and very few events of productive gene duplication. As expected, both whales share most of these genomic hallmarks, which probably reflect milestones in their evolution, including immune challenges, diet specialization, skin adaptation to the aquatic environment and changes in blood pressure and coagulation. Nevertheless, there are also some features specific for bowhead whale (Fig. S4).

Immunity and inflammation

The immune system and inflammatory pathways must respond to a very different environment in aquatic mammals compared to their terrestrial counterparts. In addition, there is a large and growing body of research on the influence of the immune system in the ageing process[3]. As long-lived mammals, whales, and particularly the bowhead whale, provide adequate models to understand the physiological adaptations that allow individuals to survive past their reproductive age[4]. Consistent with this, we have found several high-impact variants in proteases related to these functions in cetaceans. Thus, the cysteine protease ***CASP12***, a modulator of the activity of inflammatory caspases, has at least one conserved premature stop codon in bowhead and minke whales (see alignments in Supplemental Data File 1). Interestingly, while this protease is conserved and functional in almost all of the terrestrial mammals, most human populations display different deleterious variants[5], presumably with the same functional consequences as the premature stop codons in whales. Human individuals who display the uninterrupted version of CASP12, as well as animal models simulating this variant, are more sensitive to infection and sepsis[6,7]. Related to this loss, we have found that one of the splicing forms of the immunoproteasome subunit ***PSMB8***, a threonine protease, was pseudogenized through a frameshift mutation causing two premature stop codons in a common ancestor to baleen whales (Supplemental Data File 1). The immunoproteasome is a modified form of the proteasome induced by interferon gamma which is important in MHC class I peptide display. Thus, while in most mammals there are two major splicing forms of this gene, both of them expressed in multiple tissues (http://www.ncbi.nlm.nih.gov/IEB/Research/Acembly/av.cgi?db=35g&c=Gene&l=PSMB8), baleen whales only have one. In humans, a missense mutation of *PSMB8* which would affect both major splicing forms, leads to an autoinflammatory syndrome with lipodystrophy[8]. Notably, ***THOP1***, another modulator of MHC class I peptide display[9], is one of the most important targets of selection in cetaceans, with specific variants which we have confirmed in bowhead whale (Supplemental Data File 1). Similarly, a bowhead whale-specific change could be the loss of ***OTUD6A***, also known as *DUB2A*, which has a putative role in the innate immune system[10,11]. However, these results need independent confirmation, since

complete losses can be mimicked by assembly artifacts. The serine protease ***PRSS33*** has been lost in cetaceans through two conserved premature stop codons (Supplemental Data File 1). Notably, all known losses of this macrophage-specific gene in mammals are independent. Chimpanzees lost *PRSS33* through an Alu-mediated recombination mechanism[12,13], whereas the orthologs in orangutans and rhesus monkeys show different premature stop codons[14]. Therefore, this protease has been independently lost in multiple mammals, including cetaceans, probably reflecting the need for quick evolution of the immune system in different circumstances. Finally, the haptoglobin cluster of serine proteases (***HP*** and ***HPN***) has been previously singled out as a target for selection in cetaceans[15]. Bowhead *HP* is not in fact an ortholog of human *HP*, but of both human *HP* and *HPR* after a primate-specific duplication. After adding human *HPR* and several additional mammalian sequences to the alignment, we have confirmed most of the cetacean-specific residue changes, with the exception of N259D, which is also an aspartic acid in dogs (ENSCAFP00000029992) (Supplemental Data File 1). This result supports the hypothesis that *HP*, encoding an antioxidant and proangiogenic protein, has undergone selective pressure in cetaceans, as has also been shown in primates. Taken together, these events show that, similar to other mammalian species, selective pressure in cetaceans has been significant on proteins involved in the immune system. It is noteworthy that some of the cetacean targets of selective pressure have also been selected in primates, in spite of their very different environment.

Coagulation and blood pressure control

Multiple coagulation factors, most of them from the S01 family of serine proteases, have been lost in bowhead and minke whales. One of these proteases, F12, has also been inactivated in dolphins (Supplemental Data File 1), and therefore its loss probably occurred at an early stage of adaptation to the aquatic medium. Thus, all three orthologs show a change in the catalytic site of the protease which would yield an inactive protease. In the case of the whales, early stop codons suggest that the protein is not produced. In humans, a deficiency in F12 causes alterations in the coagulation process[16]. This shows one example of how adaptation to a new environment is sometimes driven through changes that may be harmful in the original circumstances, in a process known as Dobzhansky anomaly. A related serine protease gene, KLKB1, has also been pseudogenized in a common ancestor to both whales, and is not found in dolphins. Both F12 and KLKB1 participate in the kinin-kallikrein system, with known roles in inflammation, blood pressure control, coagulation and pain. In fact, a genome association analysis has found variants of these serine proteases related to increased levels of vasoactive peptides[17]. Another protease involved in this system, MME or neprilysin, has been singled out as one the preferential targets of selection in cetaceans[15], with specific changes that we have also found in bowhead whale (Supplemental Data File 1). Similarly, ACE2 and LNPEP, involved in the related renin-angiotensin system, show multiple cetacean-specific sites with functional consequences, which we have confirmed in bowhead whales[15]. Finally, the related serine proteases F7, TMPRSS11F and TMPRSS11B are pseudogenes in bowhead and minke whales, but seem to be functional genes in dolphins. These changes suggest that the mammalian potential for clotting and blood

pressure are excessive in an aquatic environment, and these systems had to be modulated through the loss of proteases implicated in related proteolytic cascades.

Digestive system

Several paralogues of carboxypeptidase A from the M14 family of metalloproteases have been pseudogenized in bowhead and minke whales. Thus, ***CPA2*** and ***CPA3*** show premature stop codons in bowhead whale (Supplemental Data File 1). Most of these stop codons are conserved in the genome of the minke whale. However, the overall sequence of the predicted proteins is well conserved, which suggests that these pseudogenization events took place recently in a common ancestor. Consistent with this, dolphins show normal orthologs for each of the human CPA genes. The pattern of specific inactivation by point mutations instead of by gene loss might be related to the fact that all CPA-like genes are clustered in the genome. This mechanism might be related to the need to preserve CPA1 and CPA5 active. Both CPA1 and CPA2 are expressed mainly in pancreas and play an important role in protein digestion and absorption[18]. Therefore, the loss of CPA2 is likely to be related to the specialized diet of cetaceans. Supporting this hypothesis, we have also found conserved premature stop codons in the cetacean orthologs of CPO (Supplemental Data File 1), an additional carboxypeptidase from the same family which is expressed in intestinal epithelial cells[19]. The specific evolution of proteases involved in the digestion of dietary proteins in cetaceans is further supported by the finding of five cetacean-specific sites in ***ANPEP***, not present in other mammals[15]. *ANPEP* encodes a metalloprotease implicated in the final digestion of peptides generated from hydrolysis of proteins by gastric and pancreatic proteases[20]. The loss of CPA3 might be related to the same adaptive mechanism, since this enzyme is also found in pancreatic secretions[21]. Interestingly, CPA3 has also been studied in connection to the modulation of innate immune response and blood pressure[22], which suggests that the loss of this protein might be involved in adaptation to the aquatic environment.

Skin

Multiple kallikreins from the S01 family of serine proteases have been likewise pseudogenized in both bowhead and minke whales (Supplemental Data File 1). Interestingly, two of the lost kallikreins, ***KLK7*** and ***KLK8***, have been implicated in skin homeostasis[23] and are also absent in dolphins. While bowhead and minke whales show conserved premature stop codons in the predicted sequence of these genes, dolphins display premature stop codons at different positions, suggesting a case of converging molecular evolution. The specific loss of two genes through independent mechanisms strongly suggests that this is an important evolutionary event, which could be related to the adaptation of the mammalian skin to aquatic environments. In fact, KLK8 has been directly related to terminal differentiation and desquamation of the stratum corneum, the outmost layer of the skin in mammals[24]. An additional skin-specific but not so well characterized serine protease, ***PRSS48***, has been similarly lost in both whales. Finally, ***CAPN12***, a cysteine protease preferentially expressed at the cortex of the hair follicle[25], has been lost in bowhead and minke whales (Supplemental Data File 1). According to these observations, some of the differential characteristics of cetacean skin, like their parakeratotic stratum corneum with

incomplete keratinization or its renewal through flaking rather than desquamation, might be related to the loss of several proteases[26,27]. Also noteworthy is the duplication of the cysteine protease ***UCHL3*** through a retrotranscription-mediated process. While this duplication seems to have happened in a common ancestor to mysticetes, only the genome of the bowhead whale shows a complete, putatively functional coding sequence for a *UCHL3*-like protease. This protease has been linked to adipogenesis, which suggests that this duplication might be related to the adaptation to the harsh arctic climate where this whale thrives.

Dentition

KLK4 was pseudogenized through a frameshift mutation in a common ancestor to both whales, but not in dolphins (Supplemental Data File 1). This protease is involved in dental enamel formation, and its pseudogenization in mammals, in concert with that of the metalloprotease ***MMP20***, leads to amelogenesis imperfecta in mammals[28,29]. The loss of MMP20 in mysticetes has been previously documented[15,30]. We have found that the pseudogenization of bowhead whale *MMP20* has followed a different path to that of minke whale (Supplemental Data File 1). Thus, unlike the minke whale ortholog, the predicted open reading frame of bowhead whale *MMP20* contains no early stop codons. Instead, the initiation methionine has been mutated to an isoleucine, which is expected to hamper translation of an active protein. Even if a different methionine residue were used as initiator, the resulting protein would lose its signal peptide, which is necessary for its extracellular function. Therefore, the loss of both *KLK4* and *MMP20* is likely to be related to the loss of teeth in the suborder Mysticeti. Even though an insertion of a SINE element has been proposed as a common mechanism for the loss of *MMP20* in mysticetes, our data support different independent mechanisms in several of the species.

Supplemental References

1 Quesada, V., Ordonez, G. R., Sanchez, L. M., Puente, X. S. & Lopez-Otin, C. The Degradome database: mammalian proteases and diseases of proteolysis. *Nucleic Acids Res* **37**, D239-243 (2009).

2 Lopez-Otin, C. & Overall, C. M. Protease degradomics: a new challenge for proteomics. *Nat Rev Mol Cell Biol* **3**, 509-519 (2002).

3 Lopez-Otin, C., Blasco, M. A., Partridge, L., Serrano, M. & Kroemer, G. The hallmarks of aging. *Cell* **153**, 1194-1217 (2013).

4 Sierra, E. *et al.* Muscular senescence in cetaceans: adaptation towards a slow muscle fibre phenotype. *Sci Rep* **3**, 1795 (2013).

5 Fischer, H., Koenig, U., Eckhart, L. & Tschachler, E. Human caspase 12 has acquired deleterious mutations. *Biochem Biophys Res Commun* **293**, 722-726 (2002).

6 Saleh, M. *et al.* Differential modulation of endotoxin responsiveness by human caspase-12 polymorphisms. *Nature* **429**, 75-79 (2004).

7 Yeretssian, G. *et al.* Gender differences in expression of the human caspase-12 long variant determines susceptibility to Listeria monocytogenes infection. *Proc Natl Acad Sci U S A* **106**, 9016-9020 (2009).

8 Kitamura, A. *et al.* A mutation in the immunoproteasome subunit PSMB8 causes autoinflammation and lipodystrophy in humans. *J Clin Invest* **121**, 4150-4160 (2011).

9 York, I. A. *et al.* The cytosolic endopeptidase, thimet oligopeptidase, destroys antigenic peptides and limits the extent of MHC class I antigen presentation. *Immunity* **18**, 429-440 (2003).

10 Kayagaki, N. *et al.* DUBA: a deubiquitinase that regulates type I interferon production. *Science* **318**, 1628-1632 (2007).

11 Meenhuis, A., Verwijmeren, C., Roovers, O. & Touw, I. P. The deubiquitinating enzyme DUB2A enhances CSF3 signalling by attenuating lysosomal routing of the CSF3 receptor. *Biochem J* **434**, 343-351 (2011).

12 Puente, X. S., Gutierrez-Fernandez, A., Ordonez, G. R., Hillier, L. W. & Lopez-Otin, C. Comparative genomic analysis of human and chimpanzee proteases. *Genomics* **86**, 638-647 (2005).

13 Johnson, M. E. *et al.* Recurrent duplication-driven transposition of DNA during hominoid evolution. *Proc Natl Acad Sci U S A* **103**, 17626-17631 (2006).

14 Locke, D. P. *et al.* Comparative and demographic analysis of orang-utan genomes. *Nature* **469**, 529-533 (2011).

15 Yim, H. S. *et al.* Minke whale genome and aquatic adaptation in cetaceans. *Nat Genet* **46**, 88-92 (2014).

16 Renne, T., Schmaier, A. H., Nickel, K. F., Blomback, M. & Maas, C. In vivo roles of factor XII. *Blood* **120**, 4296-4303 (2012).

17 Verweij, N. *et al.* Genome-wide association study on plasma levels of midregional-proadrenomedullin and C-terminal-pro-endothelin-1. *Hypertension* **61**, 602-608 (2013).

18 Szmola, R. *et al.* Chymotrypsin C is a co-activator of human pancreatic procarboxypeptidases A1 and A2. *J Biol Chem* **286**, 1819-1827 (2011).

19 Lyons, P. J. & Fricker, L. D. Carboxypeptidase O is a glycosylphosphatidylinositol-anchored intestinal peptidase with acidic amino acid specificity. *J Biol Chem* **286**, 39023-39032 (2011).

20 Fairweather, S. J., Broer, A., O'Mara, M. L. & Broer, S. Intestinal peptidases form functional complexes with the neutral amino acid transporter B(0)AT1. *Biochem J* **446**, 135-148 (2012).

21 Whitcomb, D. C. & Lowe, M. E. Human pancreatic digestive enzymes. *Dig Dis Sci* **52**, 1-17 (2007).

22 Pejler, G., Knight, S. D., Henningsson, F. & Wernersson, S. Novel insights into the biological function of mast cell carboxypeptidase A. *Trends Immunol* **30**, 401-408 (2009).
23 Kishibe, M. *et al.* Kallikrein 8 is involved in skin desquamation in cooperation with other kallikreins. *J Biol Chem* **282**, 5834-5841 (2007).
24 Kuwae, K. *et al.* Epidermal expression of serine protease, neuropsin (KLK8) in normal and pathological skin samples. *Mol Pathol* **55**, 235-241 (2002).
25 Dear, T. N., Meier, N. T., Hunn, M. & Boehm, T. Gene structure, chromosomal localization, and expression pattern of Capn12, a new member of the calpain large subunit gene family. *Genomics* **68**, 152-160 (2000).
26 Spearman, R. I. The epidermal stratum corneum of the whale. *J Anat* **113**, 373-381 (1972).
27 Haldiman, J. T. *et al.* Epidermal and papillary dermal characteristics of the bowhead whale (Balaena mysticetus). *Anat Rec* **211**, 391-402 (1985).
28 Wang, S. K. *et al.* Novel KLK4 and MMP20 mutations discovered by whole-exome sequencing. *J Dent Res* **92**, 266-271 (2013).
29 Yamakoshi, Y. *et al.* Enamel proteins and proteases in Mmp20 and Klk4 null and double-null mice. *Eur J Oral Sci* **119 Suppl 1**, 206-216 (2011).
30 Meredith, R. W., Gatesy, J., Cheng, J. & Springer, M. S. Pseudogenization of the tooth gene enamelysin (MMP20) in the common ancestor of extant baleen whales. *Proc Biol Sci* **278**, 993-1002 (2011).
