# Supplementary material for: Insights into the Evolution of Longevity from the Bowhead Whale Genome
Source: Cell Rep. 2015 Jan 6;10(1):112–22. doi: 10.1016/j.celrep.2014.12.008 (PMC4536333; doi:10.1016/j.celrep.2014.12.008)
Supplement: Supplemental Folder 1. Full List of Transposable Elements Distribution, Full List of Filtered and Unfiltered Gene Duplications, Gene Loss Alignments, Full List of Candidates under Selection, Selective Pressure Variation QC Alignments, and Full List of Bowhead-Specific Mutations, Related to Results [file mmc2.zip › gene_loss_alignments.pdf]

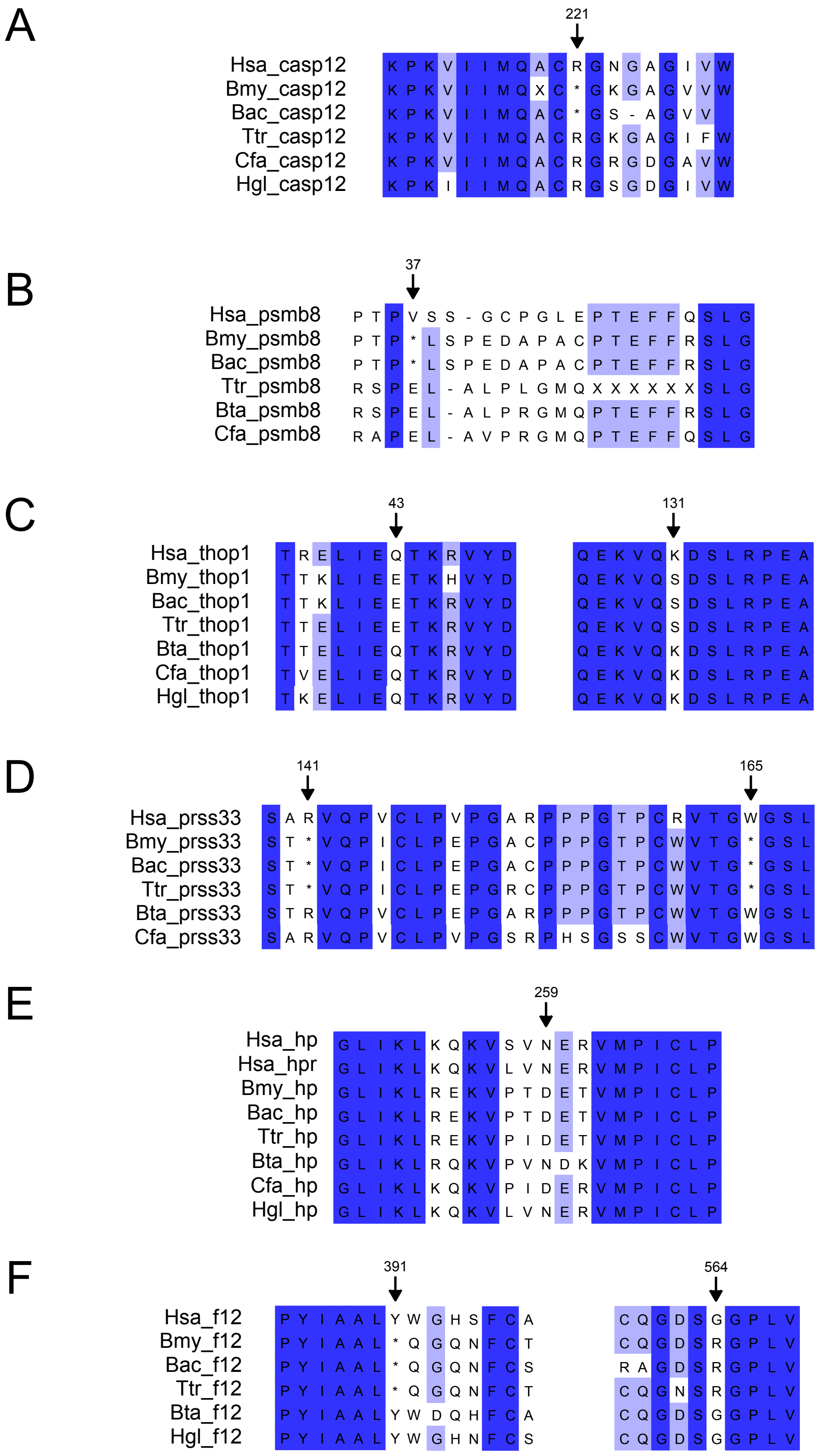

Alignments for selected sequences in cetaceans and other mammals. Numbers correspond to human proteases. *Hsa*, human; *Bmy*, bowhead whale; *Bac*, minke whale; *Ttr*, bottlenose dolphin; *Bta*, cow; *Cfa*, dog; *Hgl*, naked mole rat (continued). Related to Results.

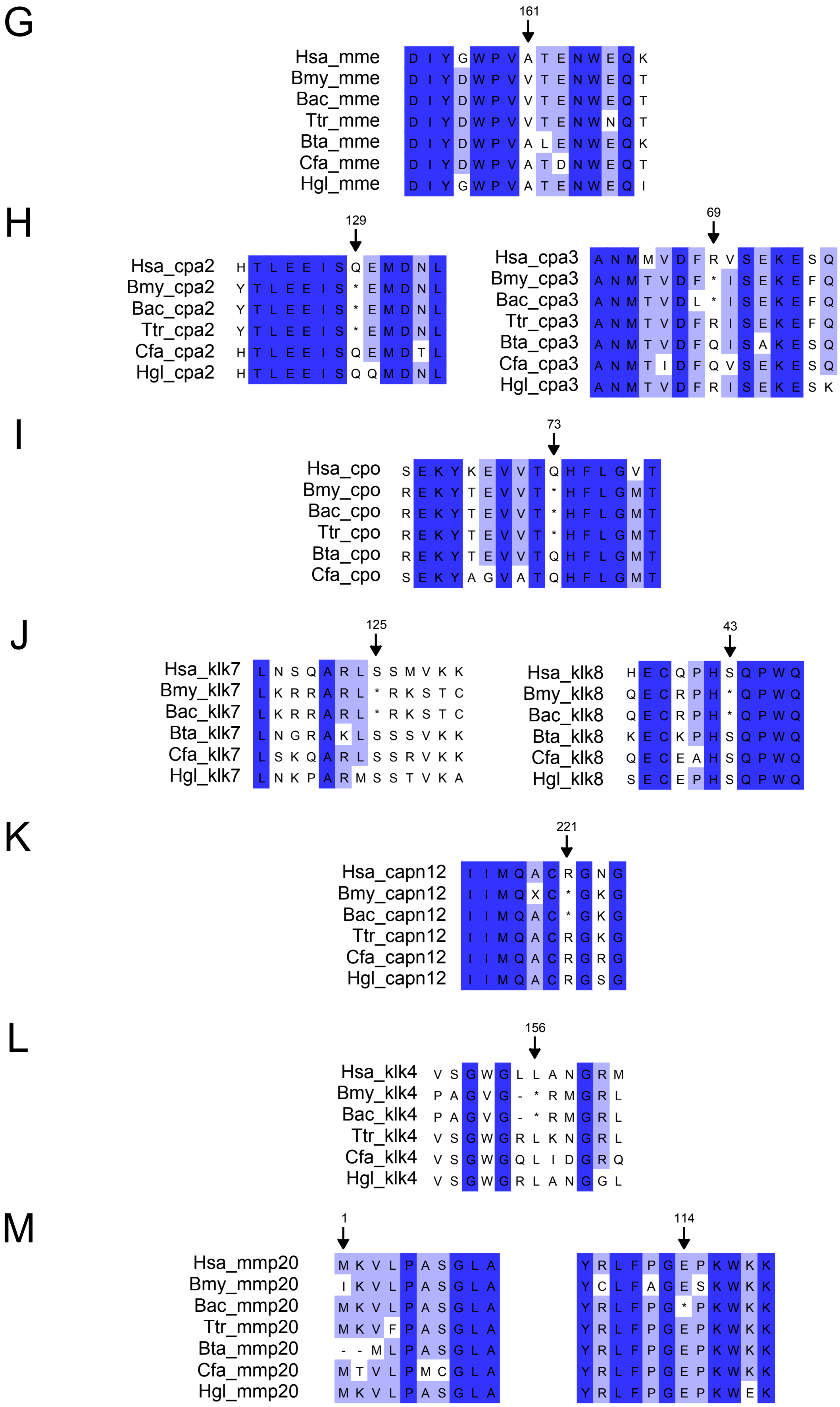

G
161
Hsa_mme D I Y G W P V A T E N W E Q K
Bmy_mme D I Y D W P V V T E N W E Q T
Bac_mme D I Y D W P V V T E N W E Q T
Ttr_mme D I Y D W P V V T E N W N Q T
Bta_mme D I Y D W P V A L E N W E Q K
Cfa_mme D I Y D W P V A T D N W E Q T
Hgl_mme D I Y G W P V A T E N W E Q I
H
129
Hsa_cpa2 H T L E E I S Q E M D N L
Bmy_cpa2 Y T L E E I S * E M D N L
Bac_cpa2 Y T L E E I S * E M D N L
Ttr_cpa2 Y T L E E I S * E M D N L
Cfa_cpa2 H T L E E I S Q E M D T L
Hgl_cpa2 H T L E E I S Q Q M D N L
69
Hsa_cpa3 A N M M V D F R V S E K E S Q
Bmy_cpa3 A N M T V D F * I S E K E F Q
Bac_cpa3 A N M T V D L * I S E K E F Q
Ttr_cpa3 A N M T V D F R I S E K E F Q
Bta_cpa3 A N M T V D F Q I S A K E S Q
Cfa_cpa3 A N M T I D F Q V S E K E S Q
Hgl_cpa3 A N M T V D F R I S E K E S K
I
73
Hsa_cpo S E K Y K E V V T Q H F L G V T
Bmy_cpo R E K Y T E V V T * H F L G M T
Bac_cpo R E K Y T E V V T * H F L G M T
Ttr_cpo R E K Y T E V V T * H F L G M T
Bta_cpo R E K Y T E V V T Q H F L G M T
Cfa_cpo S E K Y A G V A T Q H F L G M T
J
125
Hsa_klk7 L N S Q A R L S S M V K K
Bmy_klk7 L K R R A R L * R K S T C
Bac_klk7 L K R R A R L * R K S T C
Bta_klk7 L N G R A K L S S S V K K
Cfa_klk7 L S K Q A R L S S R V K K
Hgl_klk7 L N K P A R M S S T V K A
43
Hsa_klk8 H E C Q P H S Q P W Q
Bmy_klk8 Q E C R P H * Q P W Q
Bac_klk8 Q E C R P H * Q P W Q
Bta_klk8 K E C K P H S Q P W Q
Cfa_klk8 Q E C E A H S Q P W Q
Hgl_klk8 S E C E P H S Q P W Q
K
221
Hsa_capn12 I I M Q A C R G N G
Bmy_capn12 I I M Q X C * G K G
Bac_capn12 I I M Q A C * G K G
Ttr_capn12 I I M Q A C R G K G
Cfa_capn12 I I M Q A C R G R G
Hgl_capn12 I I M Q A C R G S G
L
156
Hsa_klk4 V S G W G L L A N G R M
Bmy_klk4 P A G V G - * R M G R L
Bac_klk4 P A G V G - * R M G R L
Ttr_klk4 V S G W G R L K N G R L
Cfa_klk4 V S G W G Q L I D G R Q
Hgl_klk4 V S G W G R L A N G G L
M
1
Hsa_mmp20 M K V L P A S G L A
Bmy_mmp20 I K V L P A S G L A
Bac_mmp20 M K V L P A S G L A
Ttr_mmp20 M K V F P A S G L A
Bta_mmp20 - - M L P A S G L A
Cfa_mmp20 M T V L P M C G L A
Hgl_mmp20 M K V L P A S G L A
114
Y R L F P G E P K W K K
Y C L F A G E S K W K K
Y R L F P G * P K W K K
Y R L F P G E P K W K K
Y R L F P G E P K W K K
Y R L F P G E P K W K K
Y R L F P G E P K W E K
