# Supplementary material for: Insights into the Evolution of Longevity from the Bowhead Whale Genome
Source: Cell Rep. 2015 Jan 6;10(1):112–22. doi: 10.1016/j.celrep.2014.12.008 (PMC4536333; doi:10.1016/j.celrep.2014.12.008)
Supplement: Document S2. Article plus Supplemental Information [file mmc4.pdf]

Cell Reports

Resource

Insights into the Evolution of Longevity from the Bowhead Whale Genome

Graphical Abstract

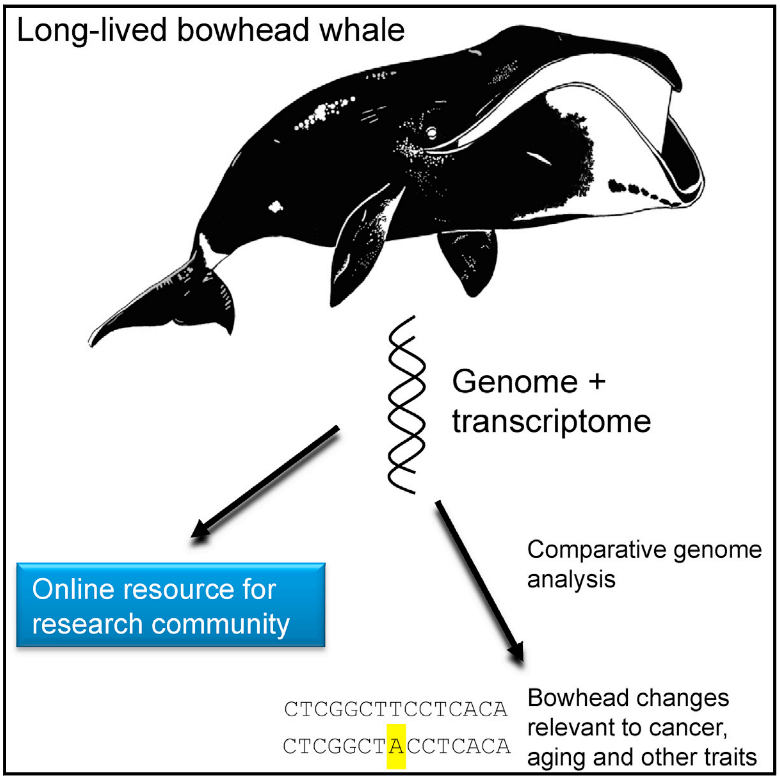

Highlights

- Genome and two transcriptomes of the bowhead whale, the longest-lived mammal
- Bowhead-specific mutations in genes associated with cancer and aging (e.g., ERCC1)
- Duplications in genes associated with DNA repair, cell cycle, and aging (e.g., PCNA)
- Changes in genes related to thermoregulation (UCP1) and other bowhead traits

Authors

Michael Keane, Jeremy Semeiks, ..., Bo Thomsen, João Pedro de Magalhães

Correspondence

jp@senescence.info

In Brief

The bowhead whale is the longest-lived mammal, possibly living over 200 years. Keane et al. sequence the bowhead genome and transcriptome and perform a comparative analysis with other cetaceans and mammals. Changes in bowhead genes related to cell cycle, DNA repair, cancer, and aging suggest alterations that may be biologically relevant.

CrossMark

Keane et al., 2015, Cell Reports *10*, 112–122
January 6, 2015 ©2015 The Authors
http://dx.doi.org/10.1016/j.celrep.2014.12.008

CellPress

Cell Reports
Resource

Insights into the Evolution of Longevity from the Bowhead Whale Genome

Michael Keane,[1,18] Jeremy Semeiks,[2,18] Andrew E. Webb,[3,18] Yang I. Li,[4,18,19] Víctor Quesada,[5,18] Thomas Craig,[1] Lone Bruhn Madsen,[6] Sipko van Dam,[1] David Brawand,[4] Patrícia I. Marques,[5] Pawel Michalak,[7] Lin Kang,[7] Jong Bhak,[8] Hyung-Soon Yim,[9] Nick V. Grishin,[2] Nynne Hjort Nielsen,[10] Mads Peter Heide-Jørgensen,[10] Elias M. Oziolor,[11] Cole W. Matson,[11] George M. Church,[12] Gary W. Stuart,[13] John C. Patton,[14] J. Craig George,[15] Robert Suydam,[15] Knud Larsen,[6] Carlos López-Otín,[5] Mary J. O'Connell,[3] John W. Bickham,[16,17] Bo Thomsen,[6] and João Pedro de Magalhães[1,*]
[1]Integrative Genomics of Ageing Group, Institute of Integrative Biology, University of Liverpool, Liverpool L69 7ZB, UK
[2]Howard Hughes Medical Institute and Departments of Biophysics and Biochemistry, University of Texas Southwestern Medical Center, Dallas, TX 75390-9050, USA
[3]Bioinformatics and Molecular Evolution Group, School of Biotechnology, Dublin City University, Glasnevin, Dublin 9, Ireland
[4]MRC Functional Genomics Unit, University of Oxford, Oxford OX1 3QX, UK
[5]Departamento de Bioquímica y Biología Molecular, Instituto Universitario de Oncología (IUOPA), Universidad de Oviedo, 33006 Oviedo, Spain
[6]Department of Molecular Biology and Genetics, Aarhus University, 8830 Tjele, Denmark
[7]Virginia Bioinformatics Institute, Virginia Tech, Blacksburg, VA 24061, USA
[8]Personal Genomics Institute, Genome Research Foundation, Suwon 443-270, Republic of Korea
[9]KIOST, Korea Institute of Ocean Science and Technology, Ansan 426–744, Republic of Korea
[10]Greenland Institute of Natural Resources, 3900 Nuuk, Greenland
[11]Department of Environmental Science, Center for Reservoir and Aquatic Systems Research (CRASR) and Institute for Biomedical Studies, Baylor University, Waco, TX 76798, USA
[12]Department of Genetics, Harvard Medical School, Boston, MA 02115, USA
[13]The Center for Genomic Advocacy (TCGA) and Department of Biology, Indiana State University, Terre Haute, IN 47809, USA
[14]Department of Forestry and Natural Resources, Purdue University, West Lafayette, IN 47907, USA
[15]North Slope Borough, Department of Wildlife Management, Barrow, AK 99723, USA
[16]Battelle Memorial Institute, Houston, TX 77079, USA
[17]Department of Wildlife and Fisheries Sciences, Texas A&M University, College Station, TX 77843, USA
[18]Co-first author
[19]Present address: Department of Genetics, Stanford University, Stanford, CA 94305, USA
*Correspondence: jp@senescence.info
http://dx.doi.org/10.1016/j.celrep.2014.12.008
This is an open access article under the CC BY license (http://creativecommons.org/licenses/by/3.0/).

SUMMARY

The bowhead whale (*Balaena mysticetus*) is estimated to live over 200 years and is possibly the longest-living mammal. These animals should possess protective molecular adaptations relevant to age-related diseases, particularly cancer. Here, we report the sequencing and comparative analysis of the bowhead whale genome and two transcriptomes from different populations. Our analysis identifies genes under positive selection and bowhead-specific mutations in genes linked to cancer and aging. In addition, we identify gene gain and loss involving genes associated with DNA repair, cell-cycle regulation, cancer, and aging. Our results expand our understanding of the evolution of mammalian longevity and suggest possible players involved in adaptive genetic changes conferring cancer resistance. We also found potentially relevant changes in genes related to additional processes, including thermoregulation, sensory perception, dietary adaptations, and immune response. Our data are made available online (http://www.bowhead-whale.org) to facilitate research in this long-lived species.

INTRODUCTION

The lifespan of some animals, including quahogs, tortoises, and certain whale species, is far greater than that of humans (Austad, 2010; Finch, 1990). It is remarkable that a warm-blooded species such as the bowhead whale (*Balaena mysticetus*) has not only been estimated to live over 200 years (estimated age of one specimen 211 SE 35 years), suggesting it is the longest-lived mammal, but also exhibits very low disease incidence until an advanced age compared to humans (George et al., 1999; Philo et al., 1993). As in humans, the evolution of longevity in whales was accompanied by low fecundity and longer developmental time (Tacutu et al., 2013), as predicted by evolutionary theory. The cellular, molecular, and genetic mechanisms underlying longevity and resistance to age-related diseases in bowhead

Table 1. Statistics of the Bowhead Whale Genome Sequencing

Sequence Data Generated		
Libraries	Total Data (Gb)	Sequence Coverage (for 2.91 Gb)
200 bp paired-end	149.1	51.2×
500 bp paired-end	141.7	48.7×
3 kb mate-paired	57.3	19.7×
5 kb mate-paired	72.5	24.9×
10 kb mate-paired	28.5	9.8×
**Total**	449.1	154.3×

Genome Assembly Statistics			
Assembly	N50 (kb)	Number	Total Size (Gb)
Contigs	34.8	113,673	2.1
Scaffolds	877	7,227	2.3

See also Figures S1 and S2.

whales are unknown, but it is clear that, in order to live so long, these animals must possess preventative mechanisms against cancer, immunosenescence, and neurodegenerative, cardiovascular, and metabolic diseases. In the context of cancer, whales, and bowhead whales, in particular, must possess effective antitumor mechanisms. Indeed, given their large size (in extreme cases adult bowhead whales can weigh up to 100 tons and are therefore among the largest whales) and exceptional longevity, bowhead whale cells must have a significantly lower probability of neoplastic transformation relative to humans (Caulin and Maley, 2011; de Magãlhaes, 2013). Therefore, studying species such as bowhead whales that have greater natural longevity and resistance to age-related diseases than humans may lead to insights on the fundamental mechanisms of aging. Here, we report the sequencing and analysis of the genome of the bowhead whale, a species of the right whale family *Balaenidae* that lives in Arctic and sub-Arctic waters. This work provides clues regarding mechanisms underlying mammalian longevity and will be a valuable resource for researchers studying the evolution of longevity, disease resistance, and basic bowhead whale biology.

RESULTS

Sequencing and Annotation of the Bowhead Whale Genome

We sequenced the nuclear genome of a female bowhead whale (*Balaena mysticetus*) using the Illumina HiSeq platform at ~150× coverage. We followed established standards in the field in terms of sequencing paired-end libraries at high coverage plus mate-paired libraries of varying (3, 5, and 10 kb) insert sizes (Table 1). Contigs and scaffolds were assembled with ALLPATHS-LG (Gnerre et al., 2011). In line with other genomes sequenced with second-generation sequencing platforms, the contig N50 was 34.8 kb and scaffold N50 was 877 kb (Table 1); the longest scaffold in our assembly was 5,861 kb. In total, our assembly is ~2.3 Gb long. Genome size was estimated experimentally to be 2.91 Gb in another female and 2.87 Gb averaged with one male (see Supplemental Results and Figure S1), but this discrepancy likely reflects highly repetitive regions, as observed for the genomes of other species with similar reported sizes such as the minke whale (Yim et al., 2014).

The full and partial completeness of the bowhead whale draft genome assembly was evaluated as 93.15% and 97.18%, respectively, by the CEGMA pipeline (Parra et al., 2007), which is comparable to the minke whale genome assembly (Yim et al., 2014). We also generated RNA sequencing (RNA-seq) data from seven adult bowhead whale tissues (cerebellum, kidney, muscle, heart, retina, liver, and testis) from specimens from Greenland and Alaska, resulting in two transcriptome assemblies (see Experimental Procedures) and annotated the genome using MAKER2, which combines ab initio methods, homology-based methods, and transcriptome data to derive gene models (Holt and Yandell, 2011). Our annotation contains 22,672 predicted protein-coding genes with an average length of 417 (median 307) amino acid residues. In addition, based on transcriptome data from two Alaskan individuals (Table S1), we estimated 0.5–0.6 SNPs per kilobase of RNA (Table S2). To begin annotation of the bowhead genome, we identified orthologs based on similarity with cow, human, and mouse genes/proteins (see Experimental Procedures), which allowed us to assign predicted gene symbols to 15,831 bowhead genes.

Moreover, to annotate microRNAs in the bowhead genome, we sequenced small RNA libraries prepared from kidney and skeletal muscle. The miRDeep algorithm (Friedländer et al., 2008, 2012) was used to integrate the sequencing data into a model of microRNA biogenesis by Dicer processing of predicted precursor hairpin structures in the genome, thus identifying 546 candidate microRNA genes. Of the 546 candidate miRNAs identified in the bowhead, 395 had seed sequences previously identified in miRNAs from human, cow, or mouse, whereas 151 did not. All of our data are available online from our Bowhead Whale Genome Resource portal (http://www.bowhead-whale.org).

Analysis of the Draft Bowhead Whale Genome

Repeat sequences make up 41% of the bowhead genome assembly, most of which (78%) belong to the group of transposable elements (TEs). Although long interspersed nuclear elements (LINEs), such as L1, and short interspersed nuclear elements (SINEs) are widespread TEs in most mammalian lineages, the bowhead genome, similar to other cetacean genomes—minke, orca, and common bottlenose dolphin—is virtually devoid of SINEs (Supplemental Folder 1). LINE-1 (L1) is the most abundant TE, particularly in orca (90%) and minke whale (89%) (Figure S2). In comparison, TE diversity (measured with Shannon's index) in the bowhead genome (0.947) is higher than in orca (0.469) and minke whale (0.515) but lower than in dolphin (1.389) and cow (Bovine Genome Sequencing and Analysis Consortium et al., 2009) (1.534).

As a first assessment of coding genes that could be responsible for bowhead whale adaptations, we used bowhead coding sequences to calculate pairwise dN/dS ratios for 9,682, 12,685, and 11,158 orthologous coding sequences from minke whale (*Balaenoptera acutorostrata*), cow (*Bos taurus*), and dolphin (*Tursiops truncatus*), respectively. It is interesting to note that there are high levels of sequence conservation in the protein coding regions between bowhead and these species: 96% (minke),

OPEN
ACCESS
CellPress

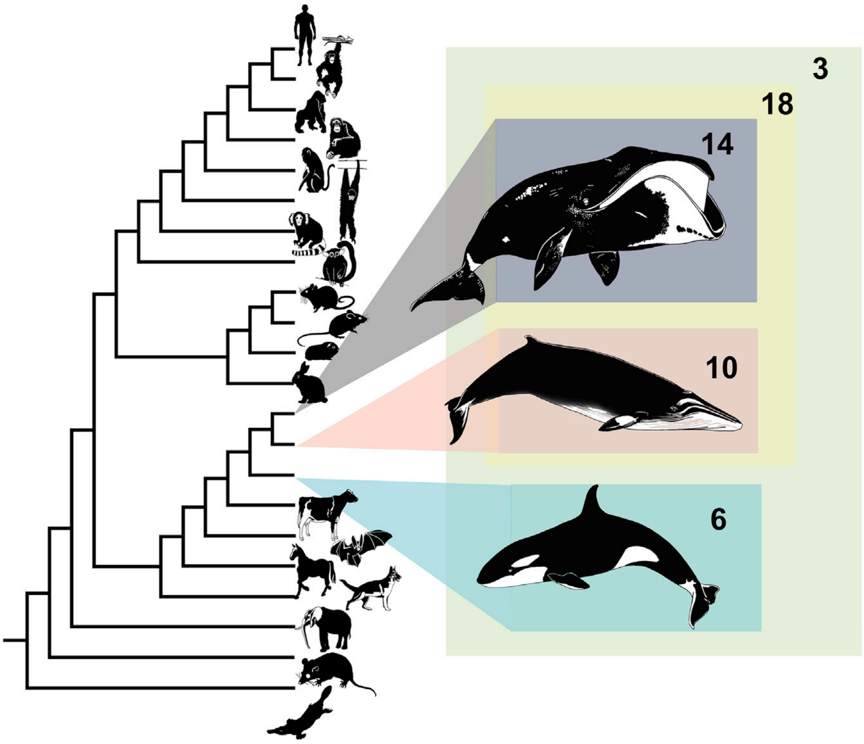

Figure 1. Phylogeny of Mammals Used in Codon-Based Maximum Likelihood Comparison of Selective Pressure Variation

The number of candidate genes under positive selection on each lineage is indicated.

92% (dolphin), and 91% (cow). This is not surprising, however, given the long generation time of cetaceans and of the bowhead whale, in particular, with animals only reaching sexual maturity at >20 years (Tacutu et al., 2013).

Because the minke whale is the closest relative to the bowhead (divergence time 25–30 million years ago [Gatesy et al., 2013]) with a sequenced genome and is smaller (<10 tons) and probably much shorter lived (maximum lifespan ~50 years) (Tacutu et al., 2013), comparisons between the bowhead and minke whale genomes may provide insights on the evolution of bowhead traits and of longevity, in particular. A number of aging- and cancer-associated genes were observed among the 420 predicted bowhead-minke orthologs with dN/dS exceeding 1, including *suppressor of cytokine signaling 2* (*SOCS2*), *aprataxin* (*APTX*), *noggin* (*NOG*), and *leptin* (*LEP*). In addition, the top 5% genes with high dN/dS values for bowhead-minke relative to the values for minke-cow and minke-dolphin orthologs included *forkhead box O3* (*FOXO3*), *excision repair cross-complementing rodent repair deficiency*, *complementation group 3* (*ERCC3*), and *fibroblast growth factor receptor 1* (*FGFR1*). The data on dN/dS ratios are also available on our portal to allow researchers to do their own analysis and quickly retrieve gene(s) of interest.

In a complementary and more detailed analysis of selective pressure variation, we used codon-based models of evolution (Yang, 2007) to identify candidate genes with evidence of lineage-specific positive selection (see Experimental Procedures). Using bowhead, minke, and orca protein-coding data along with a variety of available high-quality completed genomes from Laurasiatheria, Euarchontoglires, marsupial, and monotreme species, we identified a total of 866 single-gene ortholog families (SGOs) (i.e., these gene families have no more than one copy in each species). We tested each of the extant whale lineages, the ancestral baleen whale, and the most recent common ancestor (MRCA) of bowhead, minke, and orca, a total of five lineages (Figure 1), for evidence of lineage-specific positive selection.

Of the two extant whales analyzed, the number of SGOs exhibiting signatures of lineage-specific positive selection were as follows: bowhead (15 gene families) and minke (ten gene families). The small number of candidates under positive selection likely reflects the high level of protein conservation between bowhead and other cetaceans as well as the stringent filtering of candidates due to data-quality concerns; all results and alignments are provided in Supplemental Folder 1. A few genes associated with disease were identified, including *BMP and activin membrane-bound inhibitor* (*BAMBI*), which has been associated with various pathologies, including cancer, and also poorly studied genes of potential interest like *GRB2-binding adaptor protein, transmembrane* (*GAPT*).

In addition to the codon-based models of evolution, we wished to identify bowhead whale specific amino acid replacement substitutions. To this end, we aligned orthologous sequences between the bowhead whale and nine other mammals—a total of 4,358 alignments (see Experimental Procedures). Lineage-specific residues identified in this way have previously been shown to be indicative of significant changes in protein function (Tian et al., 2013). Our analysis revealed several proteins associated with aging and cancer among the top 5% of unique bowhead residues by concentration (i.e., normalized by protein length), including ERCC1 (excision repair cross-complementing rodent repair deficiency, complementation group 1), HDAC1 (histone deacetylase 1), and HDAC2 (Figure 2A). ERCC1 is a member of the nucleotide excision repair pathway (Gillet and Schärer, 2006), and disruption results in greatly reduced lifespan in mice and accelerated aging (Weeda et al., 1997). Histone deacetylases play an important role in the regulation of chromatin structure and transcription (Lee et al., 1993) and have been associated with longevity in *Drosophila* (Rogina et al., 2002). As such, these represent candidates involved in adaptive genetic changes conferring disease resistance in the bowhead whale. The full results are available in Supplemental Folder 1.

In addition to genes related to longevity, several interesting candidate genes emerged from our analysis of lineage-specific residues of potential relevance to other bowhead traits. Of

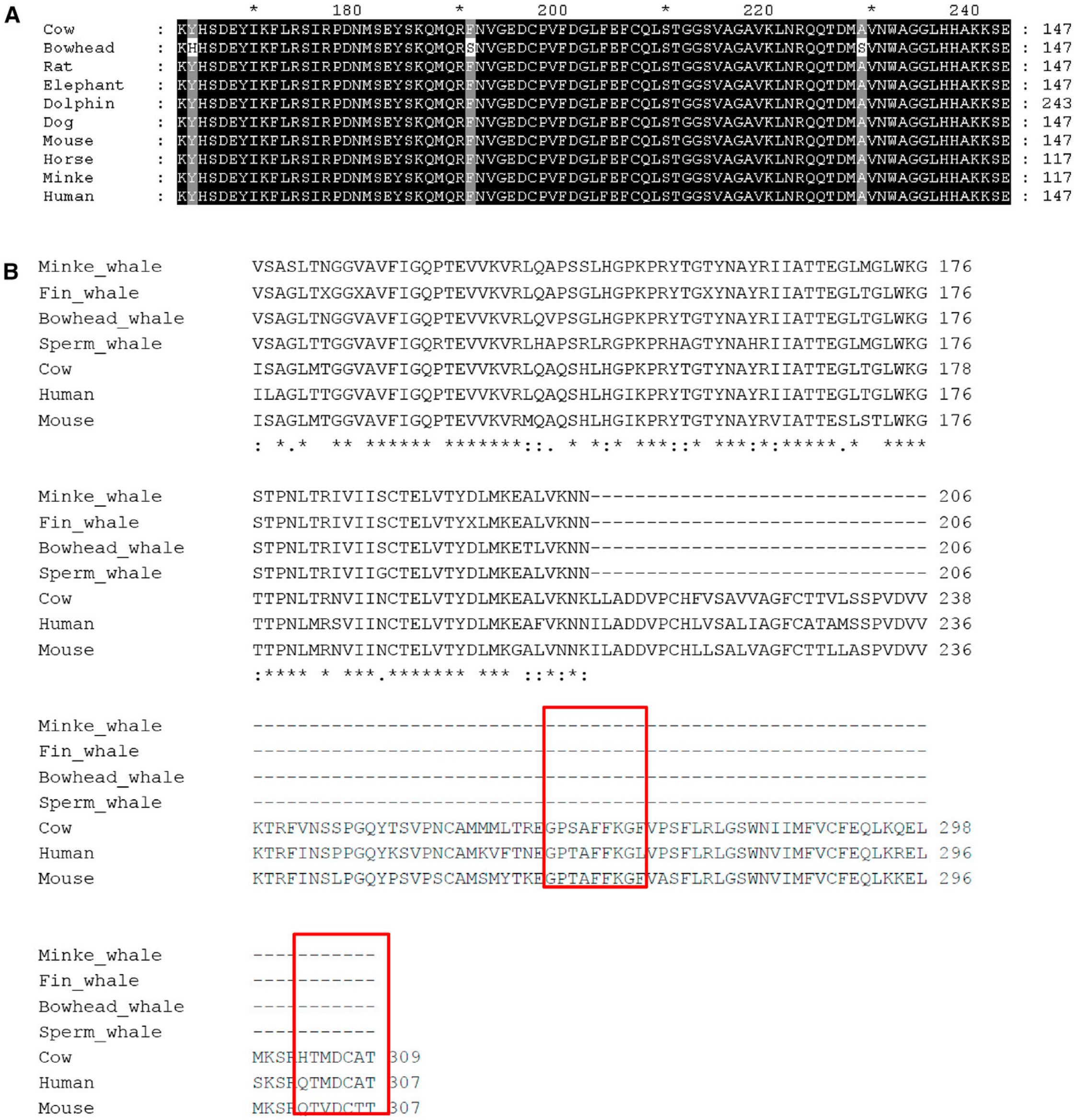

Figure 2. Multiple Protein Sequence Alignments of HDAC2 and UCP1

(A) Partial alignment of bowhead HDAC2 with mammalian orthologs. Unique bowhead residues are highlighted at human positions 68, 95, and 133.

(B) Partial alignment of whale UCP1 with mammalian orthologs. Conserved regions involved in UCP1 are marked in red.

note, a number of proteins related to sensory perception of sound were also identified with bowhead-specific mutations, including otoraplin (OTOR) and cholinergic receptor, nicotinic, alpha 10 (CHRNA10), which could be relevant in the context of the bowhead's ability to produce high- and low-frequency tones simultaneously (Tervo et al., 2011). In addition, many proteins must play roles in the large differences in size and development between the bowhead and related species and our results reveal possible candidates for further functional studies; for example, in the top ten proteins, SNX3 (sorting nexin 3) has been associated in one patient with eye formation defects and microcephaly (Vervoort et al., 2002), and WDR5 (WD repeat-containing protein 5) has been associated with osteoblast differentiation and bone development (Gori et al., 2006).

In the naked mole rat, a poikilotherm with a low metabolic rate and body temperature when compared to other mammals, unique changes in uncoupling protein 1 (UCP1), which is used to generate heat, have been previously found (Kim et al., 2011). Because the specific metabolic power output of cells in vivo for large whales must be much less than for smaller mammals (West et al., 2002), it is interesting to note that UCP1 of whales has a premature stop codon in C-terminal region, which is functionally important and conserved in other mammals (Figure 2B). It is tempting to speculate that these changes are related

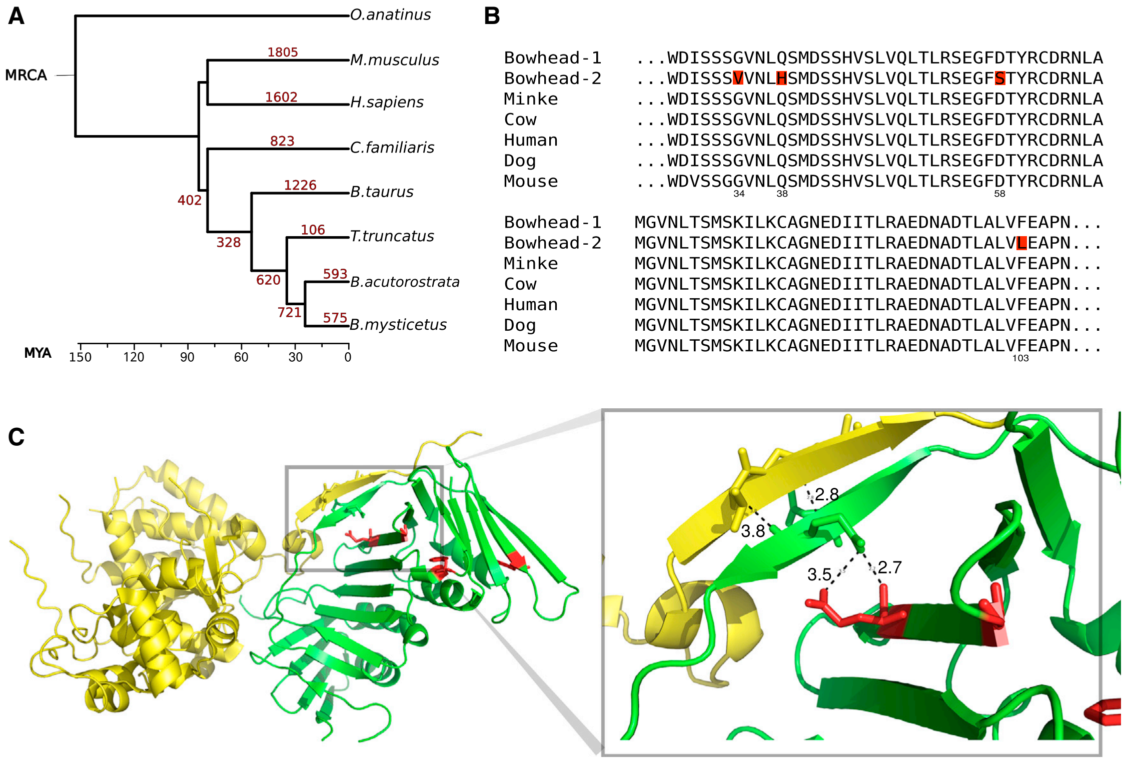

Figure 3. Gene Family Expansion and PCNA

(A) Gene family expansion. Numbers in red correspond to the predicted number of gene expansion events during mammalian evolution. Mean divergence time estimates were used from TimeTree (Hedges et al., 2006) for scaling.

(B) Multiple sequence alignment of PCNA residues 28–107, showing bowhead whale-specific duplication (gene IDs: bmy 16007 and bmy 21945). Lineage-specic amino acids in the duplicated PCNA of bowhead whales are highlighted in red.

(C) Crystal structure of the PCNA (green) and FEN-1 (yellow) complex. Lineage-specific residues on the PCNA structure are colored in red. A zoom in on the structures reveals a putative interaction between two β sheets, one within PCNA and another within FEN-1. This interaction may be altered through a second interaction between the PCNA β sheet and a lineage-specic change from glutamine to histidine within PCNA. Distance measurements between pairs of atoms are marked in black. PDB accession number: 1UL1.

See also Table S3 and Figure S3.

to differences in thermoregulation between whales and smaller mammals.

Potential Gene Duplications and Gene Losses

Gene duplication is a major mechanism through which phenotypic innovations can evolve (Holland et al., 1994; Kaessmann, 2010). Examples of mammalian phenotypic innovations associated to gene duplication include duplication of *RNASE1*, a pancreatic ribonuclease gene, in leaf-eating monkeys that contributed to adaptative changes in diet and digestive physiology (Zhang et al., 2002), a duplication of *GLUD1* in hominoids that subsequently acquired brain-specific functions (Burki and Kaessmann, 2004), and domestication of two syncytin gene copies that contributed to the emergence of placental development in mammals (Dupressoir et al., 2009). We surveyed the bowhead whale genome for expanded gene families that may reflect lineage-specific phenotypic adaptations and traits.

In the bowhead whale lineage, 575 gene families were predicted to have expanded (Figure 3). However, because gene expansion predictions are susceptible to false-positives owing to pseudogenes and annotation artifacts among other biases, we applied a stringent filter based on percentage of identity (Experimental Procedures) that reduced the number of candidate expansions to 41 (see Supplemental Folder 1 for the complete list). A functional enrichment analysis of these gene families, using default parameters in DAVID (Huang et al., 2009), only revealed a statistically significant enrichment (after correction for multiple hypothesis testing; Bonferroni <0.001) for genes associated with translation/ribosome. Given the association between translation and aging, for instance, in the context of loss of proteostasis (López-Otín et al., 2013), it is possible that these results reflect relevant adaptations in the bowhead whale.

Upon manual inspection of the gene expansion results, we found several duplicates of note. For instance, *proliferating cell nuclear antigen* (*PCNA*) is duplicated in bowhead whales with one copy harboring four lineage-specific residue changes (Figure 3B). Based on our RNA-seq data mapped to the genome (see Experimental Procedures and full results in Supplemental Folder 1), both *PCNA* copies are expressed in bowhead whale muscle, kidney, retina, and testis. By mapping the lineage-specific residues onto the structure of PCNA in complex with

FEN-1, we uncovered one amino acid substitution (Q38H), which may affect the interaction between PCNA and FEN-1 (Figure 3C). A subsequent branch-site test for selective pressure variation (see Experimental Procedures and Table S3) revealed that one substitution, D58S, may have undergone positive selection in the bowhead-whale lineage (with a posterior probability score of 0.983). The duplication of *PCNA* during bowhead-whale evolution is of particular interest due to its involvement in DNA damage repair (Hoege et al., 2002) and association with aging in that its levels in aged rat liver seem to relate to the decrease in the rate of cell proliferation (Tanno et al., 1996).

Another notable duplicated gene is *late endosomal/lysosomal adaptor, MAPK and MTOR activator 1 (LAMTOR1)*, in which six bowhead-specific amino acid changes were identified (Figure S3). LAMTOR1 is involved in amino acid sensing and activation of mTORC1, a gene strongly associated with aging and cancer (Cornu et al., 2013). The original *LAMTOR1* copy was expressed in all bowhead whale adult tissues for which we have data, with the duplicate having much lower (but detectable) expression in heart and retina. Also of note, putative duplications of *26S proteasome non-ATPase regulatory subunit 4 (PSMD4)* and *ubiquitin carboxyl-terminal esterase L3* (*UCHL3*) were identified with evidence of expression, which is intriguing considering the known involvement of the proteasome-ubiquitin system in aging (López-Otín et al., 2013) and given previous evidence that this system is under selection specific to lineages where longevity increased (Li and de Magalhães, 2013); *UCHL3* has also been involved in neurodegeneration (Kurihara et al., 2001). Other gene duplications of potential interest for their role in mitosis, cancer, and stress response include *cAMP-regulated phosphoprotein 19* (*ARPP19*), which has three copies even though we only detected expression of two copies, *stomatin-like 2* (*STOML2*), *heat shock factor binding protein 1* (*HSBP1*) with four copies of which two appear to be expressed, *spermine synthase (SMS)* and *suppression of tumorigenicity 13* (*ST13*).

Similar to previous genome characterizations, we chose the complete set of known protease genes for a detailed supervised analysis of gene loss (Quesada et al., 2009). This procedure highlighted multiple gene loss events potentially related to the evolution of several cetacean traits, including adaptations affecting the immune system, blood homeostasis, digestive system, and dentition (Figure S4). Thus, the cysteine protease CASP12, a modulator of the activity of inflammatory caspases, has at least one conserved premature stop codon in bowhead and minke whales. Interestingly, whereas this protease is conserved and functional in almost all of the terrestrial mammals, most human populations display different deleterious variants (Fischer et al., 2002), presumably with the same functional consequences as the premature stop codons in whales. Likewise, two paralogues of carboxypeptidase A (CPA2 and CPA3) have been pseudogenized in bowhead and minke whales. Notably, CPA variants have been associated with increased risk for prostate cancer in humans (Ross et al., 2009), which could be of interest in the context of reduced cancer susceptibility in whales compared with humans (de Magalhães, 2013).

Additionally, we found that multiple coagulation factors have been lost in bowhead and minke whales. The finding of bowhead whale-specific changes is also noteworthy because it could be related to the special characteristics of this mammal. For example, OTUD6A, a cysteine protease with a putative role in the innate immune system (Kayagaki et al., 2007), is specifically lacking in the assembled genome and expressed sequences of the bowhead whale. In addition, whereas the enamel metalloprotease MMP20 has been lost in bowhead and minke whales (Yim et al., 2014), our analysis suggests that these genomic events happened independently (see alignments in Supplemental Folder 1). Finally, as aforementioned, the cysteine protease UCHL3 seems to have been duplicated through a retrotranscription-mediated event in a common ancestor to bowhead and minke whales, although only the genome of the bowhead whale shows a complete, putatively functional open reading frame for this extra copy of the gene. UCHL3 may play a role in adipogenesis (van Beekum et al., 2012), which indicates that this duplication might be related to the adaptation of the bowhead whale to the challenging arctic environment. These results suggest specific scenarios for the role of proteolysis in the evolution of *Mysticetes*. Specifically, given the relationship between immunity and aging (López-Otín et al., 2013), some of these findings might open new approaches for the study of this outstanding cetacean.

DISCUSSION

The genetic and molecular mechanisms by which longevity evolves remain largely unexplained. Given the declining costs of DNA sequencing, de novo genome sequencing is rapidly becoming affordable. The sequencing of genomes of long-lived species allows comparative genomics to be employed to study the evolution of longevity and has already provided candidate genes for further functional studies (de Magalhães and Keane, 2013). Nonetheless, deciphering the genetic basis of species differences in longevity has major intrinsic challenges (de Magalhães and Keane, 2013), and much work remains to uncover the underlying mechanisms by which some species live much longer than others. In this context, studying a species so long lived and with such an extraordinary resistance to age-related diseases as the bowhead whale will help elucidate mechanisms and genes conferring longevity and disease resistance in mammals. Remarkably, large whales with over 1,000 times more cells than humans do not exhibit an increased cancer risk (Caulin and Maley, 2011), suggesting the existence of natural mechanisms that can suppress cancer more effectively in these animals. Having the genome sequence of the bowhead whale will allow researchers to study basic molecular processes and identify maintenance mechanisms that help preserve life, avoid entropy, and repair molecular damage. When compared to transcriptome data (Seim et al., 2014), the genome's greater completeness and quality permits additional (e.g., gene loss and duplication) and more thorough analyses. Besides, whereas the genomes of many commercially important agricultural species have been reported, the bowhead genome sequence is the first for a species key to a subsistence diet of indigenous communities. One of the outputs of this project will be to facilitate and drive research in this long-lived species. Data and results from this project are thus made freely available to the scientific community on an online portal (http://www.bowhead-whale.org/). We provide

this key resource for studying the bowhead whale and its various traits, including its exceptional longevity and resistance to diseases.

EXPERIMENTAL PROCEDURES

DNA and RNA Sampling in Greenland

Bowhead (*Balaena mysticetus*) DNA used for genome sequencing was isolated from muscle tissue sampled from a 51-year-old female (ID no. 325) caught in the Disko Bay, West Greenland in 2009 (Heide-Jørgensen et al., 2012). Tissue samples were stored at –20°C immediately after collection. Age estimation was performed using the aspartic acid racemization technique (Garde et al., 2007). CITES no. 12GL1003387 was used for transfer of biological material. Bowhead RNA used for RNA-seq and small RNA analysis was isolated from two different individuals: kidney samples were from a 44-year-old female (ID no. 500) and muscle samples were isolated from a 44-year-old male (ID no. 322). For more details of the individual whales, see Heide-Jørgensen et al. (2012).

Genome Sequencing

DNA was extracted following standard protocols, quantified using Qubit and run on an agarose gel to ensure no degradation had occurred. We then generated ~150× coverage of the genome using the Illumina HiSeq 2000 platform with 100 bp reads, sequencing paired-end libraries, and mate-paired libraries with insert sizes of 3, 5, and 10 kb (Table 1). Sequencing was performed at the Liverpool Centre for Genomic Research (CGR; http://www.liv.ac.uk/genomic-research/).

Genome Assembly

Libraries were preprocessed in-house by the CGR to remove adaptor sequences. The raw fastq files were trimmed for the presence of the Illumina adaptor sequence using Cutadapt and then subjected to window-based quality trimming using Sickle with a minimum window quality score of 20. A minimum read-length filter of 10 bp was also applied. Libraries were then assembled with ALLPATHS-LG (Gnerre et al., 2011), which performed all assembly steps including read error correction, initial read alignment, and scaffolding. ALLPATHS-LG build 43762 was used with the default input parameters, including K = 96. Several build parameters were automatically determined by the software at run time per its standard algorithm. Of 2.88×10^9 paired fragment reads and 1.87×10^9 paired jumping reads, 0.015% were removed as poly(A) and 1.5% were removed due to low-frequency kmers; 54% of jumping read pairs were error-corrected, and overall 33% of jumping pairs were redundant. In total, we used 216 Gbp for the 2.3 Gb assembly, meaning that coverage retained for the assembly was ~95×. Full assembly and read usage data are shown in Supplemental Folder 2. Assembly completeness was assayed with CEGMA by searching for 248 core eukaryotic genes (Parra et al., 2007).

Genome Size Determination

To determine the genome size for bowhead whale, spleen tissues were acquired from one male (10B17) and one female (10B18). Both whales were harvested in 2010 as part of the native subsistence hunt in Barrow, Alaska. Sample processing and staining followed the methods of Vindeløv and Christensen (1994). Instrument description and additional methodological details are provided in Oziolor et al. (2014). Briefly, flow cytometric genome size determination is based on propidium iodide fluorescent staining of nuclear DNA. Mean fluorescence is calculated for cells in the G0 and G1 phases of the cell cycle. This method requires direct comparison to known standards to convert measured fluorescence to pg of DNA. The primary standard used in this study was the domestic chicken (*Gallus gallus domesticus*). Chicken red blood cells are widely used as a genome size standard, with an accepted genome size of C = 1.25 pg. Chicken whole blood was purchased from Innovative Research. Mouse (*Mus musculus*) and rat (*Rattus norvegicus*) were included as internal checks, with estimates for both falling within 3% of previously published genome size estimates (Vinogradov, 1998). Spleen tissues from three male 129/SvEvTac laboratory mice and a single male Harlan SD Sprague-Dawley laboratory rat were used.

Transcriptome Sequencing and Assembly: Greenland Samples

Total RNA was extracted from the kidney and muscle employing the mirVanaTM RNA extraction kit (Ambion). RNA integrity of the individual RNA samples was assessed on a 1% agarose gel using an Agilent 2100 Bioanalyzer (Agilent Technologies). Library preparation was performed using the ScriptSeqTM mRNA-seq library preparation kit from Epicenter according to the manufacturer's protocol (Epicenter) and sequenced (100 bp paired end) as multiplexed samples using the Illumina HiSeq 2000 analyzer. Fastq generation and demultiplexing were performed using the CASAVA 1.8.2 package (Illumina). The fastq files were filtered for adapters, quality, and length using Trimmomatic (v.0.27), with a window size of 4, a base quality cutoff of 20, and a minimum length of 60 (Lohse et al., 2012). De novo transcriptome assembly was performed using the short read assembler software Trinity (release 2013-02-25), which is based on the de Bruijn graph method for assembly, with default settings (Grabherr et al., 2011).

Transcriptome Sequencing and Assembly: Alaskan Samples

Tissue biopsies were obtained from two male bowhead whales harvested by Inupiat hunters at Barrow, Alaska during the Fall hunt of 2010; heart, cerebellum, liver, and testes were biopsied from male bowhead number 10B16, and retina from male bowhead 10B20. Samples were immediately placed in liquid nitrogen and transported in a dry shipper to Purdue University. RNA was extracted using TRIZOL reagent (Invitrogen) following the manufacturer's protocol. RNA was purified using an Invitrogen PureLink Micro-to-Midi columns from the Total RNA Purification System using the standard protocol. RNA quantity and quality was estimated with a spectrophotometer (Nanodrop) and by gel electrophoresis using an Agilent model 2100 Bioanalyzer. cDNA libraries were constructed by random priming of chemically sheared poly A captured RNA. Randomly primed DNA products were blunt ended. Products from 450–650 bp were then isolated using a PippenPrep. After the addition of an adenine to the fragments, a Y primer amplification was used to produce properly tailed products. Paired-end sequences of 100 bp per end were generated using the Illumina HiScan platform. Sequences with primer concatamers, weak signal, and/or poly A/T tails were culled. The Trinity software package for de novo assembly (Grabherr et al., 2011) was used for transcript reconstruction (Table S1).

Small RNA Sequencing and Annotation

To annotate microRNA genes in the bowhead genome, we conducted deep sequencing of two small RNA libraries prepared from muscle and kidney tissues (Greenland samples). Total RNA was isolated using mirVana miRNA Isolation Kit (Ambion). Small RNA in the 15-40 nucleotides range was gel purified and small RNA libraries were prepared for next-generation sequencing using the ScriptMiner Small RNA-Seq Library Preparation Kit (Epicenter). The two libraries were sequenced on an Illumina Hi-Seq 2000 instrument to generate single end sequences of 50 nucleotides. Primary data analysis was done using the Illumina CASAVA Pipeline software v.1.8.2, and the sequence reads were further processed by trimming for adapters and filtering for low quality using Trimmomatic (Lohse et al., 2012). Identification of conserved and novel candidate microRNA genes in the bowhead genome was accomplished by applying the miRDeep2 algorithm (Friedländer et al., 2008, 2012).

Evaluation of Repeat Elements

To evaluate the percentage of repeat elements, RepeatMasker (v.4.0.3; http://www.repeatmasker.org/) was used to identify repeat elements, with parameters set as "-s -species mammal." RMBlast was used as a sequence search engine to list out all types of repeats. Percentage of repeat elements was calculated as the total number of repeat region divided by the total length of the genome, excluding the N-region. Genomes of minke whale (*Balaenoptera acutorostrata*), orca (*Orcinus orca*), common bottlenose dolphin (*Tursiops truncates*), and cow (*Bos taurus*) were downloaded from NCBI and run in parallel for comparison with the bowhead genome.

Genome Annotation

Putative genes were located in the assembly by structural annotation with MAKER2 (Holt and Yandell, 2011), which combined both bowhead

transcriptomes with comparative and de novo prediction methods including BLASTX, Exonerate, SNAP, Genemark, and Augustus. In addition to the RNA-seq data, the entire SwissProt database and the draft proteome of dolphin were used as input to the comparative methods. Repetitive elements were found with RepeatMasker (http://www.repeatmasker.org/). The complete set of MAKER input parameters, including training sets used for the de novo prediction methods, are listed in Supplemental Folder 2. In total, 22,672 protein-coding genes were predicted with an average length of 417 (median 307) amino acid residues.

The RNA-seq data from seven adult bowhead tissues described above were then mapped to the genome: FastQC (http://www.bioinformatics.babraham.ac.uk/projects/fastqc/) was used for quality control to make sure that data of all seven samples was of acceptable quality. STAR (Dobin et al., 2013) was used to generate genome files from the bowhead assembly and to map the reads to the bowhead genome with 70.3% of reads mapping, which is in line with other results including those in the minke whale (Yim et al., 2014). To count the reads overlapping genes, we used ReadCounter (van Dam et al., 2015). The results obtained from all seven samples were combined into a single file describing the number of nonambiguously mapping reads for each gene (full results in Supplemental Folder 1). Of the 22,672 predicted protein-coding genes, 89.5% had at least ten reads mapping and 97.5% of predicted genes had at least one read mapping to them, which is again comparable to other genomes like the minke whale genome (Yim et al., 2014).

To allow the identification of orthologous relationships with bowhead proteins, all cow protein sequences were downloaded from Ensembl (Flicek et al., 2013). Cow was initially used because it is the closest relative to the bowhead with a high-quality annotated genome available. First, BLASTP (10^{-5}) was used to find the best hit in the cow proteome for every predicted bowhead protein, and then the reciprocal best hit for each cow protein was defined as an ortholog. In addition, human and mouse orthologs from the OPTIC pipeline (see below) were used to assign predicted gene symbols to genes and proteins. A total of 15,831 bowhead genes have a putative gene symbol based on these predictions. Homologs in minke whale and dolphin were also derived and are available on our bowhead genome portal.

Genome Portal

To facilitate further studies of these animals, we constructed an online genome portal: The Bowhead Whale Genome Resource (http://www.bowhead-whale.org/). Its database structure, interface, and functionality were adapted from our existing Naked Mole Rat Genome Resource (Keane et al., 2014). Our data and results are available from the portal, and supplemental methods and data files are also available on GitHub (https://github.com/maglab/bowhead-whale-supplementary).

Pairwise dN/dS Analysis

The CodeML program from the PAML package was used to calculate pairwise dN/dS ratios (Yang, 2007). This is done using the ratio of nonsynonymous substitutions per nonsynonymous site (dN) to synonymous substitutions per synonymous site (dS), dN/dS, or ω (Yang, 2007). Specifically, these pairwise dN/dS ratios were calculated for bowhead coding sequences and orthologous sequences from minke, cow, and dolphin, excluding coding sequences that were less than 50% of the length of the orthologous sequence. The results were then ranked by decreasing dN/dS and are available on our bowhead genome portal. In addition, the ratio of the bowhead-minke dN/dS value to the higher of the dN/dS values for minke-cow and minke-dolphin was calculated to identify genes that evolved more rapidly on the bowhead lineage.

Assessment of Selective Pressure Variation across Single-Gene Orthologous Families Using Codon-Based Models of Evolution

To accurately assess variation in selective pressure on the bowhead, minke, and orca lineages in comparison to extant terrestrial mammals, we created a protein-coding database spanning the placental mammals. Along with the orca (http://www.ncbi.nlm.nih.gov/bioproject/189949), minke (Yim et al., 2014), and bowhead data described above, we extracted protein coding sequences from Ensembl Biomart v.73 (Flicek et al., 2013) for the following 18 genomes: chimpanzee, cow, dog, elephant, gibbon (5.6× coverage), gorilla, guinea pig, horse, human, macaque, marmoset, microbat, mouse, opossum, orangutan, platypus, rabbit, and rat. These genomes were all high coverage (mostly >6× coverage) with the exception of gibbon (Supplemental Folder 2). Sequence similarity searches were performed using mpi-BLAST (v 1.6.0) (Altschul et al., 1990) (http://www.mpiblast.org/) on all proteins using a threshold of 10^{-7}. Gene families were identified using in-house software that clusters genes based on reciprocal BLAST hits (Altschul et al., 1990). We identified a total of 6,630 gene families from which we extracted the single-gene orthologous families (SGOs). Families were considered SGOs if we identified a single-gene representative in each species (one-to-one orthologs), and to account for lower coverage genomes and missing data we also considered cases where a specific gene was not present in a species, i.e., one-to-zero orthology. SGOs were only considered for subsequent analysis if they contained more than seven species in total and if they contained no internal stop codons (indicative of sequencing errors). In total, we retained 866 SGOs for further analysis. Multiple sequence alignments (MSAs) were generated using default parameters in PRANK (v.100802) (Löytynoja and Goldman, 2008). To minimize potential false-positives due to poor sequence quality, the MSAs of the 866 SGOs underwent strict data-quality filtering. The first filter prohibited the presence of gaps in the MSA if created by unique insertions (>12 bp) in either bowhead or minke sequences. The second filter required unaligned bowhead or minke sequences to be at least half the length of their respective MSA. These two filters refined the number of testable SGOs to 319. The gene phylogeny of each SGO was inferred from the species phylogeny (Morgan et al., 2013). CodeML from the PAML software package (v.4.4e) (Yang, 2007) was employed for our selective pressure variation analyses. We analyzed each of the 319 refined SGOs using the nested codon-based models of evolution under a maximum likelihood framework. We employed the likelihood ratio test (LRT) using nested models of sequence evolution to evaluate a variety of models of codon sequence evolution (Yang, 2007). In general, these codon models allow for variable dN/dS ratios (referred to as ω throughout) among sites in the alignment, along different lineages on our phylogenetic tree, or a combination of both variations across lineages and sites. To assess the significance of fit of each model to the data, we used the recommended LRTs in CodeML (Yang, 2007) for comparing nested models (see Supplemental Folder 2). The LRT test statistic approximates the chi-square ($\chi 2$) distribution critical value with degrees of freedom equal to the number of additional free parameters in the alternative model. The goal of the codon-based modeling is to determine the selective pressures at work in a lineage and site-specific manner.

The models applied follow the standard nomenclature (i.e., model M1, M2, A, and A null) (Yang, 2007). Model M1 assumes that there are two classes of sites—those with an ω value of zero and those with an ω value of 1. Model M2 allows for three classes of sites—one with an ω value of zero, one with an ω value of one and one with an ω value that is not fixed to any value. Given the relationship between M1 and M2, they can be tested for the significance of the difference of the fit of these two models using an LRT with df = 2. Finally, we used model A that allows the ω value to vary across sites and across different lineages in combination. With model A, we can estimate the proportion of sites and the dN/dS ratio in the foreground lineage of interest in comparison to the background lineages and the estimated dN/dS ratio is free to vary above 1 (i.e., positive selection). Model A can be compared with its site-specific counterpart (model M1) using the LRT with df = 2. In addition, the lineage and site-specific model model A null was applied as a second LRT with model A. In model A null, the additional site category is fixed at neutral rather than being estimated from the data, and this LRT provides an additional test for model A (Zhang et al., 2005). In this way, we performed independent tests on each of the extant cetacean lineages (orca, minke, and bowhead), as well as testing each ancestral cetacean branch (the MRCA of the two baleen whales and the MRCA of all three cetaceans), to determine if there were signatures of positive selection that are unique to each lineage (Yang and dos Reis, 2011). Using empirical Bayesian estimations, we identified the specific residues that are positively selected in each lineage tested. Positive selection was inferred if all of the following criteria were met: (1) if the LRT was significant, (2) if the parameters estimated under that model were concurrent with positive selection, and (3) if the alignment in that region was of high quality (as judged by alignment

completeness and quality in that region). The posterior probability (PP) of a positively selected site is estimated using two calculations: Naive Empirical Bayes (NEB) or Bayes Empirical Bayes (BEB) (Yang, 2007). If both NEB and BEB are predicted, we reported the BEB results as they have been shown to be more robust under certain conditions (Yang et al., 2005). For all models used in the analysis where ω is estimated from the data, a variety of starting ω values was used for the calculation of likelihood estimates. This ensures that the global minimum is reached.

Identification of Proteins with Bowhead-Unique Residues

An in-house Perl pipeline was used to align each bowhead protein with orthologs from nine other mammals: human (*Homo sapiens*), dog (*Canis familiaris*), mouse (*Mus musculus*), rat (*Rattus norvegicus*), minke whale (*Balaenoptera acutorostrata*), cow (*Bos taurus*), dolphin (*Tursiops truncatus*), horse (*Equus caballus*), and elephant (*Loxodonta africana*) and then identify the unique bowhead amino acid residues. Gaps were excluded from the analysis, and a maximum of one unknown residue was allowed in species other than the bowhead. The results were ranked by the number of unique residues normalized by the protein length (full results in Supplemental Folder 1).

Gene Expansion Analysis, Filtering, and Expression

Human, mouse, dog, cow, dolphin, and platypus genomes and gene annotations were obtained from Ensembl (Flicek et al., 2013), the genome and gene annotation of minke whale were obtained from Yim et al. (2014). In total, 21,069, 22,275, 19,292, 19,988, 15,769, 17,936, 20,496, and 22,733 human, mouse, dog, cow, dolphin, platypus, minke whale, and bowhead whale genes, respectively, were used to construct orthology mappings using OPTIC (Heger and Ponting, 2007). Briefly, OPTIC builds phylogenetic trees for gene families by first assigning orthology relationships based on pairwise orthologs computed using PhyOP (Goodstadt and Ponting, 2006). Then, a tree-based method, PhyOP, is used to cluster genes into orthologous groups, and, last, gene members are aligned and phylogenetic trees built with TreeBeST (Vilella et al., 2009). Further details are available in the OPTIC paper (Heger and Ponting, 2007). Predicted orthology groups can be accessed at http://genserv.anat.ox.ac.uk/clades/vertebrates_bowhead.

To identify gene families that underwent expansion, gene trees were reconciled with the consensus species tree, and duplicated nodes were identified. The tree used, derived from TimeTree (Hedges et al., 2006), was: (mm_oanatinus5, ((mm_cfamiliaris3, (mm_btaurus, (mm_ttruncatus, (mm_balaenoptera, mm_bmysticetus)))), (mm_hsapiens10, mm_mmusculus5))). The following algorithm was used to reconcile gene and species trees.

A stringent filter was applied to the data so that gene duplicates in bowhead whales were required to differ by at most 10% in protein sequence from a cognate copy but were also required to differ by at least 1% to avoid assembly artifacts and to remove recently duplicated copies with no function. Further manual inspection of the alignments was performed. Gene expression inferred from our RNA-seq data was used to check the expression of duplicates.

An in-house peptide-sensitive approach was used to align the PCNA cDNA into codons, and CodeML/PAML was used to test M0, a one-rate model that assumes the same rate of evolution in all branches against M2^a, a branch site test with one rate for the background and one rate for the bowhead whale branch (Yang, 2007).

ACCESSION NUMBERS

Our data and results can be downloaded from the Bowhead Whale Genome Resource (http://www.bowhead-whale.org/downloads/). In addition, data are available at the NCBI BioProject PRJNA194091 with raw sequencing reads in the Sequence Read Archive (SRP050351).

SUPPLEMENTAL INFORMATION

Supplemental Information includes Supplemental Results, four figures, three tables, and two supplemental data files and can be found with this article online at http://dx.doi.org/10.1016/j.celrep.2014.12.008.

AUTHOR CONTRIBUTIONS

G.M.C., J.C.G., R.S., J.W.B., B.T., and J.P.M. conceived and designed the study; L.B.M., E.M.O., and C.W.M. performed the experiments; M.K., J.S., A.E.W., Y.I.L., V.Q., L.B.M., S.v.D., D.B., P.I.M., P.M., L.K., J.B., H.-S.Y., G.W.S., J.C.P., C.L.-O., M.J.O., J.W.C., and J.P.M. analyzed the data; T.C., N.V.G., N.H.N., M.P.H.-J., R.S., and K.L. contributed reagents/materials/analysis tools; and M.K. and J.P.M. wrote the paper.

ACKNOWLEDGMENTS

This project was supported by grants from the Life Extension Foundation and the Methuselah Foundation to J.P.M. We thank the Inuit whaling captains of the Alaska Eskimo Whaling Commission and the Barrow Whaling Captains' Association for allowing us to sample their whales and their willingness to support the transcriptome study. This study was also partly funded by the Augustinus Foundation to K.L. T.C. was supported by a Wellcome Trust grant (WT094386MA) to J.P.M. and M.K. was supported by a studentship from the University of Liverpool's Faculty of Health and Life Sciences. J.S. was supported by grants from the NIH (GM094575 to N.V.G.) and the Welch Foundation (I-1505 to N.V.G.). Y.I.L. was supported by a Nuffield Department of Medicine Prize studentship from the University of Oxford. C.L.-O. is an Investigator of the Botin Foundation also supported by grants from Ministerio de Economía y Competitividad-Spain and Instituto de Salud Carlos III (RTICC)-Spain. M.J.O. and A.E.W. are funded by Science Foundation Ireland Research Frontiers Programme Grant (EOB2763) to M.J.O. M.J.O. would also like to acknowledge the Fulbright Commission for the Fulbright Scholar Award 2012-2013. M.J.O. and A.E.W. thank the SFI/HEA Irish Centre for High-End Computing (ICHEC) for processor time and technical support. This work was also supported by the Korea Institute of Ocean Science and Technology (KIOST) in-house program (PE99212). Further thanks to Prof. Chris Ponting and Dr. Andreas Heger for hosting gene orthology predictions from OPTIC, the University of Liverpool High Performance Computing facilities for processor time, Eric de Sousa for help with RNA-seq data analysis, and to Louise Crompton for assistance in compiling and formatting the bibliography. Last, we are grateful to the staff at the Liverpool Centre for Genomic Research for advice during this project.

Received: September 7, 2014
Revised: November 21, 2014
Accepted: December 3, 2014
Published: December 24, 2014

REFERENCES

Altschul, S.F., Gish, W., Miller, W., Myers, E.W., and Lipman, D.J. (1990). Basic local alignment search tool. J. Mol. Biol. *215*, 403–410.

Austad, S.N. (2010). Methusaleh's Zoo: how nature provides us with clues for extending human health span. J. Comp. Pathol. *142* (*Suppl 1*), S10–S21.

Burki, F., and Kaessmann, H. (2004). Birth and adaptive evolution of a hominoid gene that supports high neurotransmitter flux. Nat. Genet. *36*, 1061–1063.

Caulin, A.F., and Maley, C.C. (2011). Peto's Paradox: evolution's prescription for cancer prevention. Trends Ecol. Evol. *26*, 175–182.

Bovine Genome Sequencing and Analysis Consortium, Elsik, C.G., Tellam, R.L., Worley, K.C., Gibbs, R.A., Muzny, D.M., Weinstock, G.M., Adelson, D.L., Eichler, E.E., Elnitski, L., et al. (2009). The genome sequence of taurine cattle: a window to ruminant biology and evolution. Science *324*, 522–528.

Cornu, M., Albert, V., and Hall, M.N. (2013). mTOR in aging, metabolism, and cancer. Curr. Opin. Genet. Dev. *23*, 53–62.

de Magalhães, J.P. (2013). How ageing processes influence cancer. Nat. Rev. Cancer *13*, 357–365.

de Magalhães, J.P., and Keane, M. (2013). Endless paces of degeneration—applying comparative genomics to study evolution's moulding of longevity. EMBO Rep. *14*, 661–662.

Dobin, A., Davis, C.A., Schlesinger, F., Drenkow, J., Zaleski, C., Jha, S., Batut, P., Chaisson, M., and Gingeras, T.R. (2013). STAR: ultrafast universal RNA-seq aligner. Bioinformatics *29*, 15–21.

Dupressoir, A., Vernochet, C., Bawa, O., Harper, F., Pierron, G., Opolon, P., and Heidmann, T. (2009). Syncytin-A knockout mice demonstrate the critical role in placentation of a fusogenic, endogenous retrovirus-derived, envelope gene. Proc. Natl. Acad. Sci. USA *106*, 12127–12132.

Finch, C. (1990). Longevity, Senescence, and the Genome (Chicago: University of Chicago Press).

Fischer, H., Koenig, U., Eckhart, L., and Tschachler, E. (2002). Human caspase 12 has acquired deleterious mutations. Biochem. Biophys. Res. Commun. *293*, 722–726.

Flicek, P., Ahmed, I., Amode, M.R., Barrell, D., Beal, K., Brent, S., Carvalho-Silva, D., Clapham, P., Coates, G., Fairley, S., et al. (2013). Ensembl 2013. Nucleic Acids Res. *41*, D48–D55.

Friedländer, M.R., Chen, W., Adamidi, C., Maaskola, J., Einspanier, R., Knespel, S., and Rajewsky, N. (2008). Discovering microRNAs from deep sequencing data using miRDeep. Nat. Biotechnol. *26*, 407–415.

Friedländer, M.R., Mackowiak, S.D., Li, N., Chen, W., and Rajewsky, N. (2012). miRDeep2 accurately identifies known and hundreds of novel microRNA genes in seven animal clades. Nucleic Acids Res. *40*, 37–52.

Garde, E., Heide-Jorgensen, M.P., Hansen, S.H., Nachman, G., and Forchhammer, M.C. (2007). Age-specific growth and remarkable longevity in narwhals (Monodon monoceros) from West Greenland as estimated by aspartic acid racemization. J. Mammal. *88*, 49–58.

Gatesy, J., Geisler, J.H., Chang, J., Buell, C., Berta, A., Meredith, R.W., Springer, M.S., and McGowen, M.R. (2013). A phylogenetic blueprint for a modern whale. Mol. Phylogenet. Evol. *66*, 479–506.

George, J.C., Bada, J., Zeh, J., Scott, L., Brown, S.E., O'Hara, T., and Suydam, R. (1999). Age and growth estimates of bowhead whales (Balaena mysticetus) via aspartic acid racemization. Can. J. Zool. *77*, 571–580.

Gillet, L.C.J., and Schärer, O.D. (2006). Molecular mechanisms of mammalian global genome nucleotide excision repair. Chem. Rev. *106*, 253–276.

Gnerre, S., Maccallum, I., Przybylski, D., Ribeiro, F.J., Burton, J.N., Walker, B.J., Sharpe, T., Hall, G., Shea, T.P., Sykes, S., et al. (2011). High-quality draft assemblies of mammalian genomes from massively parallel sequence data. Proc. Natl. Acad. Sci. USA *108*, 1513–1518.

Goodstadt, L., and Ponting, C.P. (2006). Phylogenetic reconstruction of orthology, paralogy, and conserved synteny for dog and human. PLoS Comput. Biol. *2*, e133.

Gori, F., Friedman, L.G., and Demay, M.B. (2006). Wdr5, a WD-40 protein, regulates osteoblast differentiation during embryonic bone development. Dev. Biol. *295*, 498–506.

Grabherr, M.G., Haas, B.J., Yassour, M., Levin, J.Z., Thompson, D.A., Amit, I., Adiconis, X., Fan, L., Raychowdhury, R., Zeng, Q., et al. (2011). Full-length transcriptome assembly from RNA-seq data without a reference genome. Nat. Biotechnol. *29*, 644–652.

Hedges, S.B., Dudley, J., and Kumar, S. (2006). TimeTree: a public knowledge-base of divergence times among organisms. Bioinformatics *22*, 2971–2972.

Heger, A., and Ponting, C.P. (2007). Evolutionary rate analyses of orthologs and paralogs from 12 Drosophila genomes. Genome Res. *17*, 1837–1849.

Heide-Jørgensen, M.P., Garde, E., Nielsen, N.H., Andersen, O.N., and Hansen, S.H. (2012). A note on biological data from the hunt of bowhead whales in West Greenland 2009-2011. J. Cetacean Res. Manag. *12*, 329–333.

Hoege, C., Pfander, B., Moldovan, G.L., Pyrowolakis, G., and Jentsch, S. (2002). RAD6-dependent DNA repair is linked to modification of PCNA by ubiquitin and SUMO. Nature *419*, 135–141.

Holland, P.W., Garcia-Fernàndez, J., Williams, N.A., and Sidow, A. (1994). Gene duplications and the origins of vertebrate development. Dev. Suppl., 125–133.

Holt, C., and Yandell, M. (2011). MAKER2: an annotation pipeline and genome-database management tool for second-generation genome projects. BMC Bioinformatics *12*, 491.

Huang, W., Sherman, B.T., and Lempicki, R.A. (2009). Systematic and integrative analysis of large gene lists using DAVID bioinformatics resources. Nat. Protoc. *4*, 44–57.

Kaessmann, H. (2010). Origins, evolution, and phenotypic impact of new genes. Genome Res. *20*, 1313–1326.

Kayagaki, N., Phung, Q., Chan, S., Chaudhari, R., Quan, C., O'Rourke, K.M., Eby, M., Pietras, E., Cheng, G., Bazan, J.F., et al. (2007). DUBA: a deubiquitinase that regulates type I interferon production. Science *318*, 1628–1632.

Keane, M., Craig, T., Alföldi, J., Berlin, A.M., Johnson, J., Seluanov, A., Gorbunova, V., Di Palma, F., Lindblad-Toh, K., Church, G.M., and de Magalhães, J.P. (2014). The Naked Mole Rat Genome Resource: facilitating analyses of cancer and longevity-related adaptations. Bioinformatics *30*, 3558–3560.

Kim, E.B., Fang, X., Fushan, A.A., Huang, Z., Lobanov, A.V., Han, L., Marino, S.M., Sun, X., Turanov, A.A., Yang, P., et al. (2011). Genome sequencing reveals insights into physiology and longevity of the naked mole rat. Nature *479*, 223–227.

Kurihara, L.J., Kikuchi, T., Wada, K., and Tilghman, S.M. (2001). Loss of Uch-L1 and Uch-L3 leads to neurodegeneration, posterior paralysis and dysphagia. Hum. Mol. Genet. *10*, 1963–1970.

Lee, D.Y., Hayes, J.J., Pruss, D., and Wolffe, A.P. (1993). A positive role for histone acetylation in transcription factor access to nucleosomal DNA. Cell *72*, 73–84.

Li, Y., and de Magalhães, J.P. (2013). Accelerated protein evolution analysis reveals genes and pathways associated with the evolution of mammalian longevity. Age (Dordr.) *35*, 301–314.

Lohse, M., Bolger, A.M., Nagel, A., Fernie, A.R., Lunn, J.E., Stitt, M., and Usadel, B. (2012). RobiNA: a user-friendly, integrated software solution for RNA-seq-based transcriptomics. Nucleic Acids Res. *40*, W622–W627.

López-Otín, C., Blasco, M.A., Partridge, L., Serrano, M., and Kroemer, G. (2013). The hallmarks of aging. Cell *153*, 1194–1217.

Löytynoja, A., and Goldman, N. (2008). A model of evolution and structure for multiple sequence alignment. Philos. Trans. R. Soc. Lond. B Biol. Sci. *363*, 3913–3919.

Morgan, C.C., Foster, P.G., Webb, A.E., Pisani, D., McInerney, J.O., and O'Connell, M.J. (2013). Heterogeneous models place the root of the placental mammal phylogeny. Mol. Biol. Evol. *30*, 2145–2156.

Oziolor, E.M., Bigorgne, E., Aguilar, L., Usenko, S., and Matson, C.W. (2014). Evolved resistance to PCB- and PAH-induced cardiac teratogenesis, and reduced CYP1A activity in Gulf killifish (Fundulus grandis) populations from the Houston Ship Channel, Texas. Aquat. Toxicol. *150*, 210–219.

Parra, G., Bradnam, K., and Korf, I. (2007). CEGMA: a pipeline to accurately annotate core genes in eukaryotic genomes. Bioinformatics *23*, 1061–1067.

Philo, L.M., Shotts, E.B., and George, J.C. (1993). Morbidity and mortality. In The Bowhead Whale, J.J. Burns, J.J. Montague, and C.J. Cowles, eds. (Lawrence, Kansas: Allen Press), pp. 275–312.

Quesada, V., Ordóñez, G.R., Sánchez, L.M., Puente, X.S., and López-Otín, C. (2009). The Degradome database: mammalian proteases and diseases of proteolysis. Nucleic Acids Res. *37*, D239–D243.

Rogina, B., Helfand, S.L., and Frankel, S. (2002). Longevity regulation by Drosophila Rpd3 deacetylase and caloric restriction. Science *298*, 1745.

Ross, P.L., Cheng, I., Liu, X., Cicek, M.S., Carroll, P.R., Casey, G., and Witte, J.S. (2009). Carboxypeptidase 4 gene variants and early-onset intermediate-to-high risk prostate cancer. BMC Cancer *9*, 69.

Seim, I., Ma, S., Zhou, X., Gerashchenko, M.V., Lee, S.G., Suydam, R., George, J.C., Bickham, J.W., and Gladyshev, V.N. (2014). The transcriptome of the bowhead whale Balaena mysticetus reveals adaptations of the longest-lived mammal. Aging (Albany, N.Y. Online) *6*, 879–899.

Tacutu, R., Craig, T., Budovsky, A., Wuttke, D., Lehmann, G., Taranukha, D., Costa, J., Fraifeld, V.E., and de Magalhães, J.P. (2013). Human Ageing

OPEN
ACCESS
CellPress

Genomic Resources: integrated databases and tools for the biology and genetics of ageing. Nucleic Acids Res. *41*, D1027–D1033.

Tanno, M., Ogihara, M., and Taguchi, T. (1996). Age-related changes in proliferating cell nuclear antigen levels. Mech. Ageing Dev. *92*, 53–66.

Tervo, O.M., Christoffersen, M.F., Parks, S.E., Kristensen, R.M., and Madsen, P.T. (2011). Evidence for simultaneous sound production in the bowhead whale (Balaena mysticetus). J. Acoust. Soc. Am. *130*, 2257–2262.

Tian, X., Azpurua, J., Hine, C., Vaidya, A., Myakishev-Rempel, M., Ablaeva, J., Mao, Z., Nevo, E., Gorbunova, V., and Seluanov, A. (2013). High-molecular-mass hyaluronan mediates the cancer resistance of the naked mole rat. Nature *499*, 346–349.

van Beekum, O., Gao, Y., Berger, R., Koppen, A., and Kalkhoven, E. (2012). A novel RNAi lethality rescue screen to identify regulators of adipogenesis. PLoS ONE *7*, e37680.

van Dam, S., Craig, T., and de Magalhães, J.P. (2015). GeneFriends: a human RNA-seq-based gene and transcript co-expression database. Nucleic Acids Res. http://dx.doi.org/10.1093/nar/gku1042

Vervoort, V.S., Viljoen, D., Smart, R., Suthers, G., DuPont, B.R., Abbott, A., and Schwartz, C.E. (2002). Sorting nexin 3 (SNX3) is disrupted in a patient with a translocation t(6;13)(q21;q12) and microcephaly, microphthalmia, ectrodactyly, prognathism (MMEP) phenotype. J. Med. Genet. *39*, 893–899.

Vilella, A.J., Severin, J., Ureta-Vidal, A., Heng, L., Durbin, R., and Birney, E. (2009). EnsemblCompara GeneTrees: Complete, duplication-aware phylogenetic trees in vertebrates. Genome Res. *19*, 327–335.

Vindeløv, L.L., and Christensen, I.J. (1994). Detergent and proteolytic enzyme-based techniques for nuclear isolation and DNA content analysis. Methods Cell Biol. *41*, 219–229.

Vinogradov, A.E. (1998). Genome size and GC-percent in vertebrates as determined by flow cytometry: the triangular relationship. Cytometry *31*, 100–109.

Weeda, G., Donker, I., de Wit, J., Morreau, H., Janssens, R., Vissers, C.J., Nigg, A., van Steeg, H., Bootsma, D., and Hoeijmakers, J.H.J. (1997). Disruption of mouse ERCC1 results in a novel repair syndrome with growth failure, nuclear abnormalities and senescence. Curr. Biol. *7*, 427–439.

West, G.B., Woodruff, W.H., and Brown, J.H. (2002). Allometric scaling of metabolic rate from molecules and mitochondria to cells and mammals. Proc. Natl. Acad. Sci. USA *99* (*Suppl 1*), 2473–2478.

Yang, Z. (2007). PAML 4: phylogenetic analysis by maximum likelihood. Mol. Biol. Evol. *24*, 1586–1591.

Yang, Z., and dos Reis, M. (2011). Statistical properties of the branch-site test of positive selection. Mol. Biol. Evol. *28*, 1217–1228.

Yang, Z., Wong, W.S.W., and Nielsen, R. (2005). Bayes empirical bayes inference of amino acid sites under positive selection. Mol. Biol. Evol. *22*, 1107–1118.

Yim, H.S., Cho, Y.S., Guang, X., Kang, S.G., Jeong, J.Y., Cha, S.S., Oh, H.M., Lee, J.H., Yang, E.C., Kwon, K.K., et al. (2014). Minke whale genome and aquatic adaptation in cetaceans. Nat. Genet. *46*, 88–92.

Zhang, J., Zhang, Y.P., and Rosenberg, H.F. (2002). Adaptive evolution of a duplicated pancreatic ribonuclease gene in a leaf-eating monkey. Nat. Genet. *30*, 411–415.

Zhang, J., Nielsen, R., and Yang, Z. (2005). Evaluation of an improved branch-site likelihood method for detecting positive selection at the molecular level. Mol. Biol. Evol. *22*, 2472–2479.

Cell Reports, Volume *10*

Supplemental Information

Insights into the Evolution of Longevity from the Bowhead Whale Genome

Michael Keane, Jeremy Semeiks, Andrew E. Webb, Yang I. Li, Víctor Quesada, Thomas Craig, Lone Bruhn Madsen, Sipko van Dam, David Brawand, Patrícia I. Marques, Pawel Michalak, Lin Kang, Jong Bhak, Hyung-Soon Yim, Nick V. Grishin, Nynne Hjort Nielsen, Mads Peter Heide-Jørgensen, Elias M. Oziolor, Cole W. Matson, George M. Church, Gary W. Stuart, John C. Patton, J. Craig George, Robert Suydam, Knud Larsen, Carlos López-Otín, Mary J. O’Connell, John W. Bickham, Bo Thomsen, and João Pedro de Magalhães

Supplemental Data

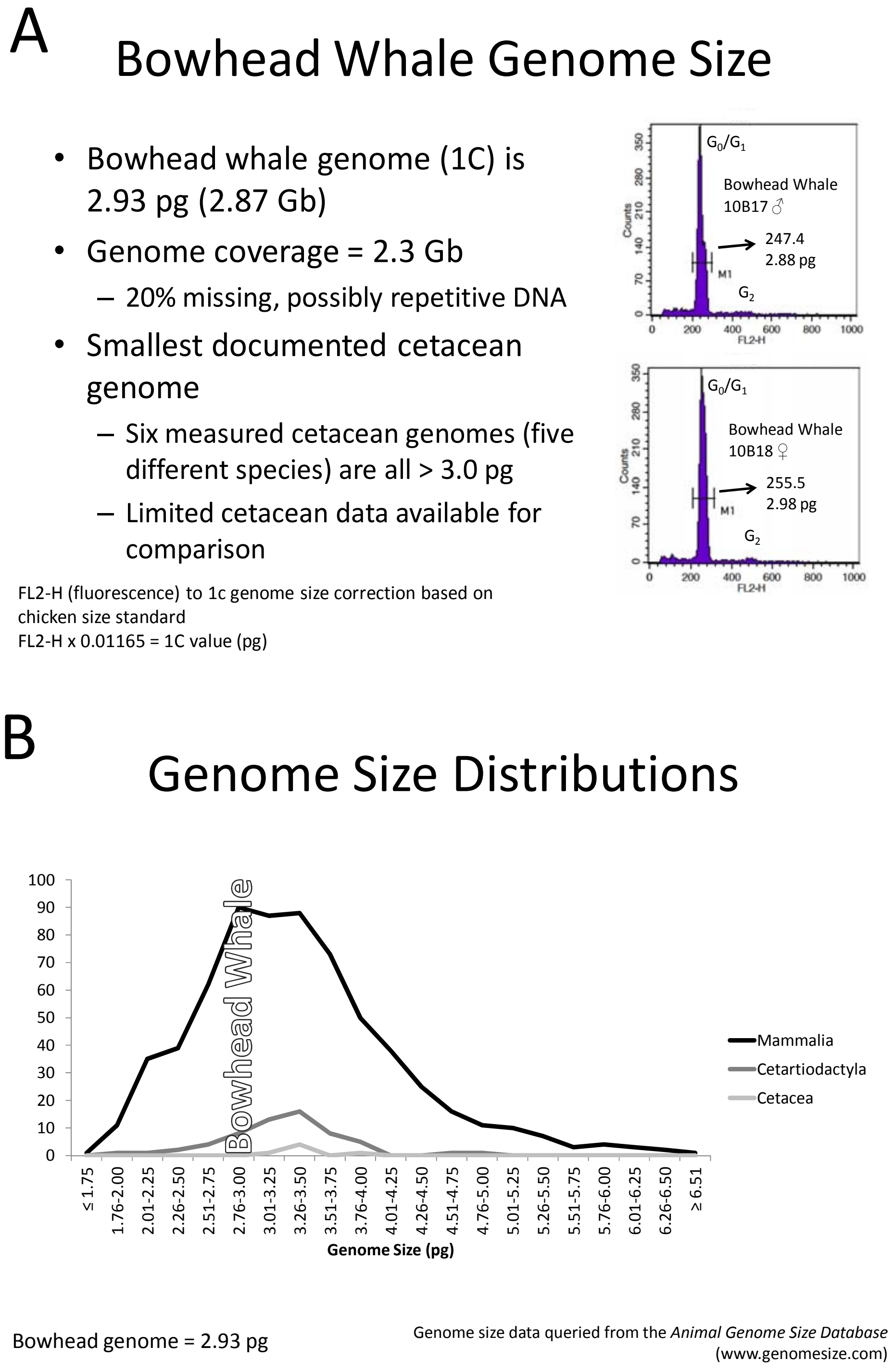

Figure S1: Bowhead whale genome size, Related to Table 1. S1A—DNA flow histograms (right two panels) of a male and female bowhead whale showing an approximately 3% difference

in estimated genome sizes. The mean estimated genome size is C = 2.93 pg. S1B—Distribution of genome sizes of Mammalia, Cetartiodactyla, and Cetacea. Bowhead whales have an estimated genome size (2.93 pg) well below the mammalian mean (3.5 pg). This is the first species of baleen whale to be reported and has the lowest C-value of any cetacean. Some cetartiodactyls have lower genome sizes but most are higher than bowheads. Related to Results.

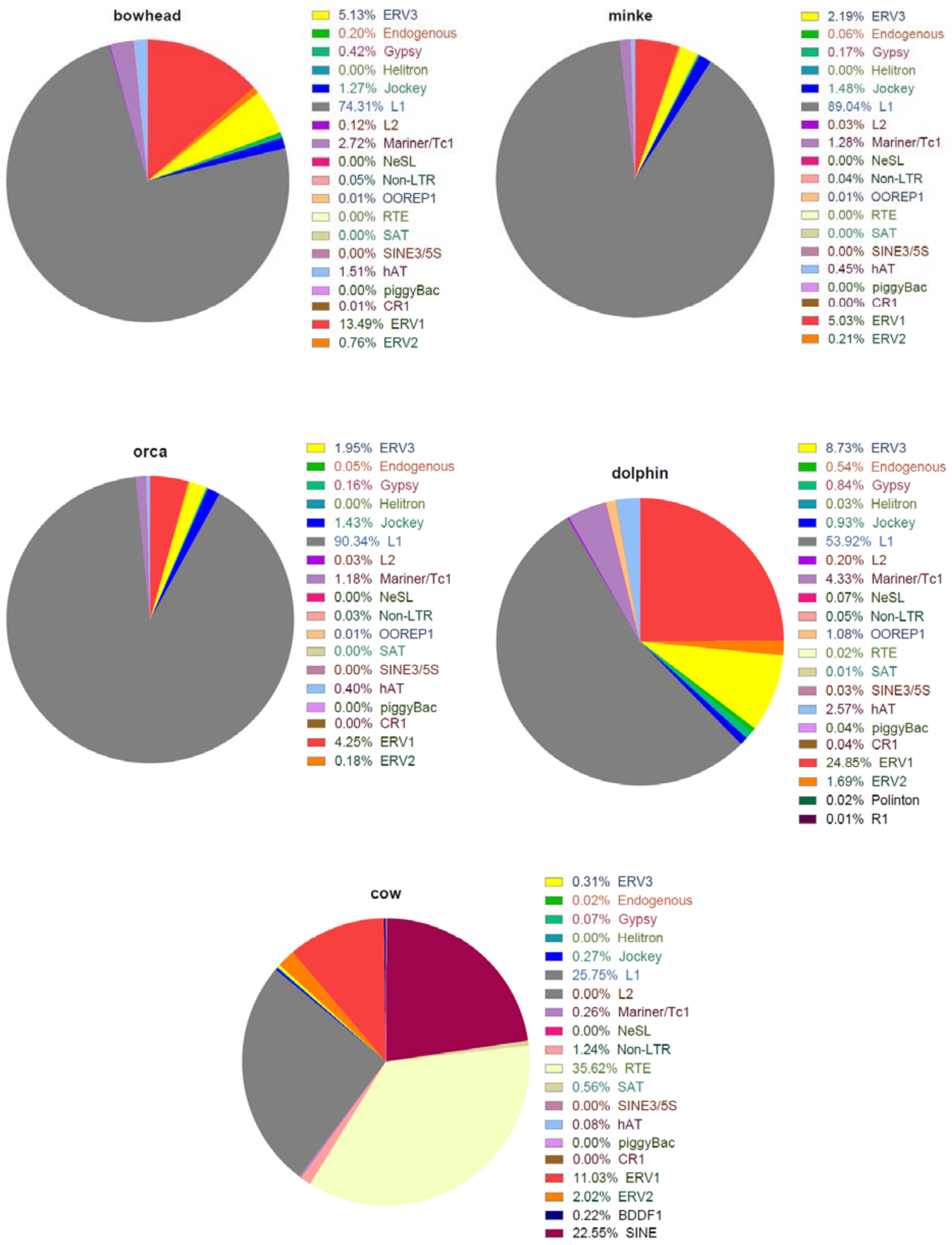

Figure S2: Repeat sequences, Related to Table 1. Transposable elements in bowhead whale and related species.

```
ENSBTAG00000003001   MGCCYSSENEDSDQDREERKLLLDPSSPPTKALNGAEPNYHSLPSARTDEQALLSSILAKTASNIIDVSAADSQG
ENSOANG00000001786   ---CKLTLPPHPRQEREERKLLLDPSSPPTKALNGTEPNYHSLPSARTDEQALLSSILAKTASNIIDVSAADSQG
ENSTTRG00000010763   MGCCYSSENEDSDQDREERKLLLDPSSPPTKALNGAEPNYHSLPSARTDEQALLSSILAKTASNIIDVSAADSQG
bmy_03663            MGCCYSSENEDSDQDREERKLLLDPSSPPTKALNGAEPNYHSLPSARTDEQALLSSILAKTASNIIDVSAADSQG
ENSMUSG00000030842   MGCCYSSENEDSDQDREERKLLLDPSSTPTKALNGAEPNYHSLPSARTDEQALLSSILAKTASNIIDVSAADSQG
ENSCAFG00000005788   MGCCYSSENEDSDQDREERKLLLDPSSPPTKALNGAEPNYHSLPPTRTDEQALLSSILAKTASNIIDVSAADSQG
ENSG00000149357      MGCCYSSENEDSDQDREERKLLLDPSSPPTKALNGAEPNYHSLPSARTDEQALLSSILAKTASNIIDVSAADSQG
BACU019752G          MGRCYGSGNGDWDQDREERKLLLDP--PPPKALNGAEPNYHSLPSARTDEQALLSSVLAKTAGNIIDVCASDSQG
bmy_21325            MACCYSSENEDSDQDREERKLLLDPSSPPTKALNGAEPNYHSLPSASTDEQALLSSILAETAGNIIDVSAADSQG


ENSBTAG00000003001   MEQHEYMDRARQYSTRLAVLSSSLTHWKKLPPLPSLTSQPHQVLASEPVPFSDLQQ-------------------
ENSOANG00000001786   MEPHEYMDRARQYSTRLAVLSSSLTHWKKLPPLPSLTNQPHQVLASDPVPFADLQQ-------------------
ENSTTRG00000010763   MEQHEYMDRARQYSTRLAVLSSSLTHWKKLPPLPSLTSQPHQVLASEPVPFADLQQ-------------------
bmy_03663            MEQHEYMDRARQYSTRLAVLSSSLTHWKKLPPLPSLTSQPHQVLASEPVPFADLQQ-------------------
ENSMUSG00000030842   MEQHEYMDRARQYSTRLAVLSSSLTHWKKLPPLPSLTSQPHQVLASEPIPFSDLQQ-------------------
ENSCAFG00000005788   MEQHEYMDRARQYSTRLAVLSSSLTHWKKLPPLPSLTSQPHQVLASEPIPFSDLQQ-------------------
ENSG00000149357      MEQHEYMDRARQYSTRLAVLSSSLTHWKKLPPLPSLTSQPHQVLASEPIPFSDLQQVRHPSPAPAHPSHTAQGMA
BACU019752G          TEQHEGVDRARQCSTCLAVLSSSLTHWEKLPPRPSLSSQPHRVLASEPVPFADWQH-------------------
bmy_21325            TERHGYMDRARQYSTRLAVLSSSLTRWEKLPPLPSLTSQPHRVLASEPVLFADLQQ-------------------


ENSBTAG00000003001   ----------VSRIAAYAYSALSQIRVDAKEELVVQFGIP----------------------
ENSOANG00000001786   ----------VSRIAAYAYSALSQIRVDAKEELVVQFGIP----------------------
ENSTTRG00000010763   ----------VSRIAAYAYSALSQIRVDAKEELVVQFGIP----------------------
bmy_03663            ----------VSRIAAYAYSALSQIRVDAKEELVVQFGIPX---------------------
ENSMUSG00000030842   ----------VSRIAAYAYSALSQIRVDAKEELVVQFGIP----------------------
ENSCAFG00000005788   ----------VSRIAAYAYSALSQIRVDAKEELVVQFGIP----------------------
ENSG00000149357      EGSPTLPQRRVSRIAAYAYSALSQIRVDAKEELVVQFGIPRHTGHHTEKELVQLFQSTPCSQ
BACU019752G          ----------VSRIAAYAYGALSQIRVDAQEELVVQFGIPX---------------------
bmy 21325            ----------VSRIAAYAYGALSQIRVDAKEELVVQFGIPX---------------------
```

Figure S3: Putative LAMTOR1 gene duplication in the bowhead, Related to Figure 3.

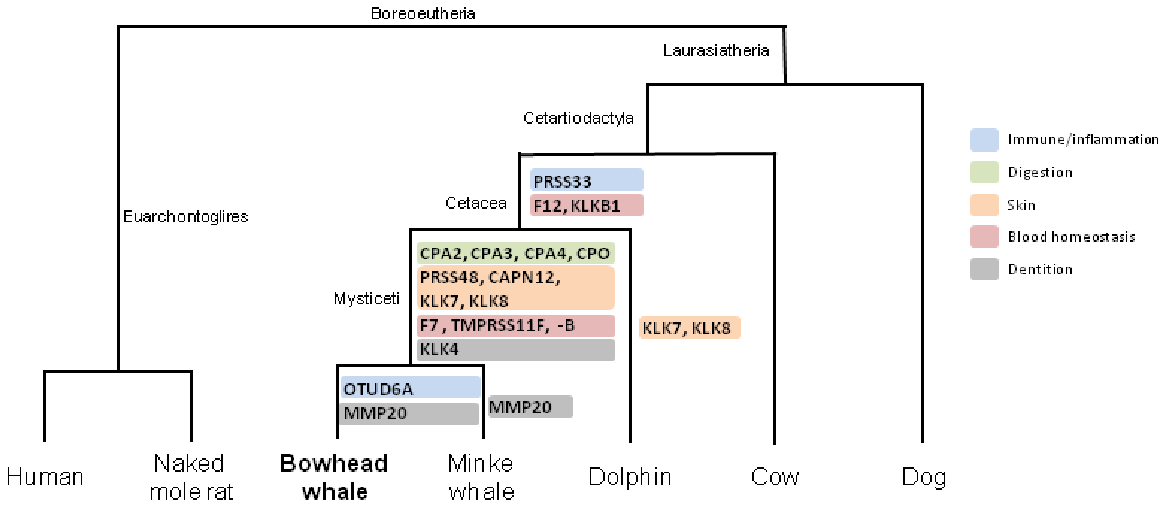

Figure S4: Genomic losses in the bowhead whale degradome, Related to Results. Each gene is depicted on the right side of the branch where each loss is inferred. Putative roles of each protease are shown in different colours.

Table S1: RNA sequencing of 5 tissues from two bowhead whales, Related to Results and Experimental Procedures. All Reads refers to all sequenced fragments of any size, Large Contigs includes contigs comprised of multiple reads of 500 bp or larger, and All Contigs refers to small and large contigs combined.

**All Reads**	**Total reads**	138,495,774
	**Total bases**	13,162,565,851
	**Size range of reads**	2-101
	**N50 (modal size)**	101
	**Average length**	95
**Large Contigs**	**Contig size**	≥500
	**Total large contigs**	157,699
	**Total number of bases**	322,342,312
	**Contig size range**	500-24765
	**N50 (modal size)**	3,442
	**Average length**	2,044
**All Contigs**	**Total number of contigs**	423,657
	**Total number of bases**	401,340,157
	**Contig size range**	201-24765
	**N50 (modal size)**	2,436
	**Average length**	947
**Annotations**	**Number of annotated contigs**	81,319

Table S2: SNP frequencies estimated for each tissue per size class of contigs, Related to Results. Tissues 1-4 are from bowhead 10B16 and retina is from 10B20.

Contig Size (bp)	Tissue				
	1. Cerebellum	2. Heart	3. Liver	4. Testes	5. Retina	Tissues 1-4
≥201	2.7E-04	2.7E-04	2.7E-04	2.8E-04	3.1E-04	3.9E-04
>500	3.3E-04	3.2E-04	3.2E-04	3.4E-04	3.8E-04	4.8E-04
>1000	3.6E-04	3.5E-04	3.6E-04	3.8E-04	4.2E-04	5.2E-04
>2000	3.9E-04	3.8E-04	3.8E-04	4.1E-04	4.5E-04	5.6E-04
>3000	4.0E-04	3.9E-04	3.9E-04	4.2E-04	4.6E-04	5.7E-04
>4000	4.2E-04	4.0E-04	4.0E-04	4.4E-04	4.7E-04	5.9E-04
>5000	4.3E-04	4.0E-04	4.0E-04	4.5E-04	4.7E-04	6.0E-04
>6000	4.5E-04	4.2E-04	4.2E-04	4.7E-04	4.9E-04	6.2E-04

Table S3: Branch-site test Bayes empirical Bayes values for putative positively selected sites in PCNA, Related to Figure 3. *Indicates statistical significance.

Site	Sub.	BEB
34	V	0.774
38	H	0.753
58	S	0.983*
103	L	0.758
231	T	0.748

Supplemental Results

Genome size estimation

Simple ratios, assuming a chicken genome size of C = 1.25 pg, were used to convert mean fluorescence to pg of DNA. Mouse and rat tissues, which were included as an additional confirmation of genome size estimation accuracy, were within 2% and 3%, respectively, of published values (data not shown). Bowhead whale genome sizes were estimated using both chicken as a size standard, and by averaging the estimates produced from all three size standards (chicken, mouse, and rat) independently. The results from these two methods yielded estimates of 2.93 and 2.92 pg, respectively. Of particular interest was the variability in individual bowhead whale genome size estimates, an approximately 3% difference between our two samples (Figure S1A). While not known during sample processing and initial analysis, bowhead #10B17, the individual with the smaller genome (2.88 pg), was a male, whereas bowhead #10B18, the individual with the larger genome (2.98 pg) was a female. This difference in genome size is entirely accounted for by the expected differences in masses of X and Y chromosomes. As is customary, the final bowhead whale genome size estimate was calculated as the average of the male and female genome sizes, 2.93 pg or 2.87 Gb (Figure S1A).

This is the first cytometric-based estimate of genome size for a baleen whale. The value C = 2.93 pg is the lowest value yet for a cetacean (Figure S1B) and is on the low end of values for Cetartiodactyla (artiodactyls and cetaceans). The average of all mammals is C = 3.5 pg, so bowheads are low for mammals. Most of the mammalian species with lower genome sizes are animal with small body size and high metabolic rates including bats, shrews and some rodents. Only toothed whales are available for comparison and thus it is not known if bowheads are atypical for baleen whales. Nevertheless it is apparent from these results that bowheads are at the low end of the scale for mammals in general.

There are two possible explanations for the relatively small genome of the bowhead whale. The first is that it could be a plesiomorphic character unchanged during the evolution and diversification of cetartiodactyls. This is possible given the fact that low genome sizes are also found in suids, camelids, giraffids, cervids and bovids, notwithstanding the fact that most cetartiodactyls have higher values (http://www.genomesize.com/) and the ancestral character state is not known.

The second possible explanation is that the low genome size of the bowhead is a derived, adaptive, character state that has evolved as a result of nucleotypic effects. A correlate to small genome size is not obvious but could be related to metabolic rate or gas exchange in this highly specialized diving mammal.

Significance of the genome size estimate of bowheads also relates to its genome sequence. There is a discrepancy in the genome size as measured in base pairs (one picogram = 978 megabases) with flow cytometry compared to the total sequence length in the genome sequence (Figure S1A). The flow cytometric method is 20% higher than the sequence total and this is likely due to the inability of the bioinformatics methods to assemble repetitive DNA sequences. So, the estimated genome size gives us an independent estimate of the amount of sequence not represented in the assembled genome sequence.

Additional studies of genome size are needed for baleen whales in order to determine if the bowhead is an outlier or if this group of mammals has an unexpectedly small genome size. In this way perhaps the adaptive correlates, if any exist, can be determined. In addition, it is anticipated that other baleen whales will be the subjects of genome sequences and a better understanding of the amount of DNA sequence not assembled is useful for determining the overall percent coverage of the genome sequence.

RNA sequencing in Alaskan specimens

Sequence analysis of RNA from 5 tissues representing two bowhead whales produced a total of 138,495,774 sequence reads comprising >13 billion bp after quality control and primer trimming. The numbers and sizes of reads and contigs are reported in Table S1. The total number of annotated contigs was 81,319. The estimated number of bowhead contigs identified as being homologous to human genes was approximately 14,000 or ca. 60% of the known human genes.

Table S2 shows the estimated frequencies of SNPs among the 5 tissues sampled. The two individuals sampled can be compared by reference to retina (bowhead 10B20) and Tissues 1-4 (bowhead 10B16). The data are shown for 8 size classes of contigs. As contigs size increases, the frequency of estimated SNPs increases. With this method, there appears to be approximately 0.5-0.6 SNPs per 1,000 bases of RNA.

Analysis of bowhead whale protease genes

Proteases form a diverse group of enzymes that share the ability to hydrolyze peptide bonds. The biological and pathological significance of this enzymatic activity has prompted the definition of the degradome as the complete repertoire of proteases in an organism[1]. From a genomic point of view, the degradome is highly attractive for several reasons. First, it is composed of a large number of genes. Thus, the human degradome includes about 600 protease genes, which represents almost 3% of the total annotated human protein-coding genes. Moreover, catalytic domains of proteases exhibit a high sequence diversity, which is further increased by the frequent attachment of auxiliary, non-proteolytic domains to the catalytic moieties[2]. Some of the protease genes have been shown to occur in genomic clusters, which is convenient for the study of short-term evolution. By contrast, most protease genes are randomly distributed throughout the annotated genomes. Therefore, the degradome forms a representative subset of the coding

genome of a species. Notably, this structural diversity also reflects the multiple biological roles of proteases in every organism. Thus, beyond their obvious role in protein digestion, proteases also mediate regulatory processes through their ability to perform highly specific reactions of proteolytic processing, which have contributed to the acquisition of different functional capacities during evolution.

The comparison of the degradomes of the bowhead whale to those of minke whale, human and other mammals shows multiple events of gene loss in cetaceans and very few events of productive gene duplication. As expected, both whales share most of these genomic hallmarks, which probably reflect milestones in their evolution, including immune challenges, diet specialization, skin adaptation to the aquatic environment and changes in blood pressure and coagulation. Nevertheless, there are also some features specific for bowhead whale (Fig. S4).

Immunity and inflammation

The immune system and inflammatory pathways must respond to a very different environment in aquatic mammals compared to their terrestrial counterparts. In addition, there is a large and growing body of research on the influence of the immune system in the ageing process[3]. As long-lived mammals, whales, and particularly the bowhead whale, provide adequate models to understand the physiological adaptations that allow individuals to survive past their reproductive age[4]. Consistent with this, we have found several high-impact variants in proteases related to these functions in cetaceans. Thus, the cysteine protease ***CASP12***, a modulator of the activity of inflammatory caspases, has at least one conserved premature stop codon in bowhead and minke whales (see alignments in Supplemental Data File 1). Interestingly, while this protease is conserved and functional in almost all of the terrestrial mammals, most human populations display different deleterious variants[5], presumably with the same functional consequences as the premature stop codons in whales. Human individuals who display the uninterrupted version of CASP12, as well as animal models simulating this variant, are more sensitive to infection and sepsis[6,7]. Related to this loss, we have found that one of the splicing forms of the immunoproteasome subunit ***PSMB8***, a threonine protease, was pseudogenized through a frameshift mutation causing two premature stop codons in a common ancestor to baleen whales (Supplemental Data File 1). The immunoproteasome is a modified form of the proteasome induced by interferon gamma which is important in MHC class I peptide display. Thus, while in most mammals there are two major splicing forms of this gene, both of them expressed in multiple tissues (http://www.ncbi.nlm.nih.gov/IEB/Research/Acembly/av.cgi?db=35g&c=Gene&l=PSMB8), baleen whales only have one. In humans, a missense mutation of *PSMB8* which would affect both major splicing forms, leads to an autoinflammatory syndrome with lipodystrophy[8]. Notably, ***THOP1***, another modulator of MHC class I peptide display[9], is one of the most important targets of selection in cetaceans, with specific variants which we have confirmed in bowhead whale (Supplemental Data File 1). Similarly, a bowhead whale-specific change could be the loss of ***OTUD6A***, also known as *DUB2A*, which has a putative role in the innate immune system[10,11]. However, these results need independent confirmation, since

complete losses can be mimicked by assembly artifacts. The serine protease ***PRSS33*** has been lost in cetaceans through two conserved premature stop codons (Supplemental Data File 1). Notably, all known losses of this macrophage-specific gene in mammals are independent. Chimpanzees lost *PRSS33* through an Alu-mediated recombination mechanism[12,13], whereas the orthologs in orangutans and rhesus monkeys show different premature stop codons[14]. Therefore, this protease has been independently lost in multiple mammals, including cetaceans, probably reflecting the need for quick evolution of the immune system in different circumstances. Finally, the haptoglobin cluster of serine proteases (***HP*** and ***HPN***) has been previously singled out as a target for selection in cetaceans[15]. Bowhead *HP* is not in fact an ortholog of human *HP*, but of both human *HP* and *HPR* after a primate-specific duplication. After adding human *HPR* and several additional mammalian sequences to the alignment, we have confirmed most of the cetacean-specific residue changes, with the exception of N259D, which is also an aspartic acid in dogs (ENSCAFP00000029992) (Supplemental Data File 1). This result supports the hypothesis that *HP*, encoding an antioxidant and proangiogenic protein, has undergone selective pressure in cetaceans, as has also been shown in primates. Taken together, these events show that, similar to other mammalian species, selective pressure in cetaceans has been significant on proteins involved in the immune system. It is noteworthy that some of the cetacean targets of selective pressure have also been selected in primates, in spite of their very different environment.

Coagulation and blood pressure control

Multiple coagulation factors, most of them from the S01 family of serine proteases, have been lost in bowhead and minke whales. One of these proteases, F12, has also been inactivated in dolphins (Supplemental Data File 1), and therefore its loss probably occurred at an early stage of adaptation to the aquatic medium. Thus, all three orthologs show a change in the catalytic site of the protease which would yield an inactive protease. In the case of the whales, early stop codons suggest that the protein is not produced. In humans, a deficiency in F12 causes alterations in the coagulation process[16]. This shows one example of how adaptation to a new environment is sometimes driven through changes that may be harmful in the original circumstances, in a process known as Dobzhansky anomaly. A related serine protease gene, KLKB1, has also been pseudogenized in a common ancestor to both whales, and is not found in dolphins. Both F12 and KLKB1 participate in the kinin-kallikrein system, with known roles in inflammation, blood pressure control, coagulation and pain. In fact, a genome association analysis has found variants of these serine proteases related to increased levels of vasoactive peptides[17]. Another protease involved in this system, MME or neprilysin, has been singled out as one the preferential targets of selection in cetaceans[15], with specific changes that we have also found in bowhead whale (Supplemental Data File 1). Similarly, ACE2 and LNPEP, involved in the related renin-angiotensin system, show multiple cetacean-specific sites with functional consequences, which we have confirmed in bowhead whales[15]. Finally, the related serine proteases F7, TMPRSS11F and TMPRSS11B are pseudogenes in bowhead and minke whales, but seem to be functional genes in dolphins. These changes suggest that the mammalian potential for clotting and blood

pressure are excessive in an aquatic environment, and these systems had to be modulated through the loss of proteases implicated in related proteolytic cascades.

Digestive system

Several paralogues of carboxypeptidase A from the M14 family of metalloproteases have been pseudogenized in bowhead and minke whales. Thus, ***CPA2*** and ***CPA3*** show premature stop codons in bowhead whale (Supplemental Data File 1). Most of these stop codons are conserved in the genome of the minke whale. However, the overall sequence of the predicted proteins is well conserved, which suggests that these pseudogenization events took place recently in a common ancestor. Consistent with this, dolphins show normal orthologs for each of the human CPA genes. The pattern of specific inactivation by point mutations instead of by gene loss might be related to the fact that all CPA-like genes are clustered in the genome. This mechanism might be related to the need to preserve CPA1 and CPA5 active. Both CPA1 and CPA2 are expressed mainly in pancreas and play an important role in protein digestion and absorption[18]. Therefore, the loss of CPA2 is likely to be related to the specialized diet of cetaceans. Supporting this hypothesis, we have also found conserved premature stop codons in the cetacean orthologs of CPO (Supplemental Data File 1), an additional carboxypeptidase from the same family which is expressed in intestinal epithelial cells[19]. The specific evolution of proteases involved in the digestion of dietary proteins in cetaceans is further supported by the finding of five cetacean-specific sites in ***ANPEP***, not present in other mammals[15]. *ANPEP* encodes a metalloprotease implicated in the final digestion of peptides generated from hydrolysis of proteins by gastric and pancreatic proteases[20]. The loss of CPA3 might be related to the same adaptive mechanism, since this enzyme is also found in pancreatic secretions[21]. Interestingly, CPA3 has also been studied in connection to the modulation of innate immune response and blood pressure[22], which suggests that the loss of this protein might be involved in adaptation to the aquatic environment.

Skin

Multiple kallikreins from the S01 family of serine proteases have been likewise pseudogenized in both bowhead and minke whales (Supplemental Data File 1). Interestingly, two of the lost kallikreins, ***KLK7*** and ***KLK8***, have been implicated in skin homeostasis[23] and are also absent in dolphins. While bowhead and minke whales show conserved premature stop codons in the predicted sequence of these genes, dolphins display premature stop codons at different positions, suggesting a case of converging molecular evolution. The specific loss of two genes through independent mechanisms strongly suggests that this is an important evolutionary event, which could be related to the adaptation of the mammalian skin to aquatic environments. In fact, KLK8 has been directly related to terminal differentiation and desquamation of the stratum corneum, the outmost layer of the skin in mammals[24]. An additional skin-specific but not so well characterized serine protease, ***PRSS48***, has been similarly lost in both whales. Finally, ***CAPN12***, a cysteine protease preferentially expressed at the cortex of the hair follicle[25], has been lost in bowhead and minke whales (Supplemental Data File 1). According to these observations, some of the differential characteristics of cetacean skin, like their parakeratotic stratum corneum with

incomplete keratinization or its renewal through flaking rather than desquamation, might be related to the loss of several proteases[26,27]. Also noteworthy is the duplication of the cysteine protease ***UCHL3*** through a retrotranscription-mediated process. While this duplication seems to have happened in a common ancestor to mysticetes, only the genome of the bowhead whale shows a complete, putatively functional coding sequence for a *UCHL3*-like protease. This protease has been linked to adipogenesis, which suggests that this duplication might be related to the adaptation to the harsh arctic climate where this whale thrives.

Dentition

KLK4 was pseudogenized through a frameshift mutation in a common ancestor to both whales, but not in dolphins (Supplemental Data File 1). This protease is involved in dental enamel formation, and its pseudogenization in mammals, in concert with that of the metalloprotease ***MMP20***, leads to amelogenesis imperfecta in mammals[28,29]. The loss of MMP20 in mysticetes has been previously documented[15,30]. We have found that the pseudogenization of bowhead whale *MMP20* has followed a different path to that of minke whale (Supplemental Data File 1). Thus, unlike the minke whale ortholog, the predicted open reading frame of bowhead whale *MMP20* contains no early stop codons. Instead, the initiation methionine has been mutated to an isoleucine, which is expected to hamper translation of an active protein. Even if a different methionine residue were used as initiator, the resulting protein would lose its signal peptide, which is necessary for its extracellular function. Therefore, the loss of both *KLK4* and *MMP20* is likely to be related to the loss of teeth in the suborder Mysticeti. Even though an insertion of a SINE element has been proposed as a common mechanism for the loss of *MMP20* in mysticetes, our data support different independent mechanisms in several of the species.

Supplemental References

1 Quesada, V., Ordonez, G. R., Sanchez, L. M., Puente, X. S. & Lopez-Otin, C. The Degradome database: mammalian proteases and diseases of proteolysis. *Nucleic Acids Res* **37**, D239-243 (2009).
2 Lopez-Otin, C. & Overall, C. M. Protease degradomics: a new challenge for proteomics. *Nat Rev Mol Cell Biol* **3**, 509-519 (2002).
3 Lopez-Otin, C., Blasco, M. A., Partridge, L., Serrano, M. & Kroemer, G. The hallmarks of aging. *Cell* **153**, 1194-1217 (2013).
4 Sierra, E. *et al.* Muscular senescence in cetaceans: adaptation towards a slow muscle fibre phenotype. *Sci Rep* **3**, 1795 (2013).
5 Fischer, H., Koenig, U., Eckhart, L. & Tschachler, E. Human caspase 12 has acquired deleterious mutations. *Biochem Biophys Res Commun* **293**, 722-726 (2002).
6 Saleh, M. *et al.* Differential modulation of endotoxin responsiveness by human caspase-12 polymorphisms. *Nature* **429**, 75-79 (2004).
7 Yeretssian, G. *et al.* Gender differences in expression of the human caspase-12 long variant determines susceptibility to Listeria monocytogenes infection. *Proc Natl Acad Sci U S A* **106**, 9016-9020 (2009).
8 Kitamura, A. *et al.* A mutation in the immunoproteasome subunit PSMB8 causes autoinflammation and lipodystrophy in humans. *J Clin Invest* **121**, 4150-4160 (2011).
9 York, I. A. *et al.* The cytosolic endopeptidase, thimet oligopeptidase, destroys antigenic peptides and limits the extent of MHC class I antigen presentation. *Immunity* **18**, 429-440 (2003).
10 Kayagaki, N. *et al.* DUBA: a deubiquitinase that regulates type I interferon production. *Science* **318**, 1628-1632 (2007).
11 Meenhuis, A., Verwijmeren, C., Roovers, O. & Touw, I. P. The deubiquitinating enzyme DUB2A enhances CSF3 signalling by attenuating lysosomal routing of the CSF3 receptor. *Biochem J* **434**, 343-351 (2011).
12 Puente, X. S., Gutierrez-Fernandez, A., Ordonez, G. R., Hillier, L. W. & Lopez-Otin, C. Comparative genomic analysis of human and chimpanzee proteases. *Genomics* **86**, 638-647 (2005).
13 Johnson, M. E. *et al.* Recurrent duplication-driven transposition of DNA during hominoid evolution. *Proc Natl Acad Sci U S A* **103**, 17626-17631 (2006).
14 Locke, D. P. *et al.* Comparative and demographic analysis of orang-utan genomes. *Nature* **469**, 529-533 (2011).
15 Yim, H. S. *et al.* Minke whale genome and aquatic adaptation in cetaceans. *Nat Genet* **46**, 88-92 (2014).
16 Renne, T., Schmaier, A. H., Nickel, K. F., Blomback, M. & Maas, C. In vivo roles of factor XII. *Blood* **120**, 4296-4303 (2012).
17 Verweij, N. *et al.* Genome-wide association study on plasma levels of midregional-proadrenomedullin and C-terminal-pro-endothelin-1. *Hypertension* **61**, 602-608 (2013).
18 Szmola, R. *et al.* Chymotrypsin C is a co-activator of human pancreatic procarboxypeptidases A1 and A2. *J Biol Chem* **286**, 1819-1827 (2011).
19 Lyons, P. J. & Fricker, L. D. Carboxypeptidase O is a glycosylphosphatidylinositol-anchored intestinal peptidase with acidic amino acid specificity. *J Biol Chem* **286**, 39023-39032 (2011).
20 Fairweather, S. J., Broer, A., O'Mara, M. L. & Broer, S. Intestinal peptidases form functional complexes with the neutral amino acid transporter B(0)AT1. *Biochem J* **446**, 135-148 (2012).
21 Whitcomb, D. C. & Lowe, M. E. Human pancreatic digestive enzymes. *Dig Dis Sci* **52**, 1-17 (2007).

22 Pejler, G., Knight, S. D., Henningsson, F. & Wernersson, S. Novel insights into the biological function of mast cell carboxypeptidase A. *Trends Immunol* **30**, 401-408 (2009).
23 Kishibe, M. *et al.* Kallikrein 8 is involved in skin desquamation in cooperation with other kallikreins. *J Biol Chem* **282**, 5834-5841 (2007).
24 Kuwae, K. *et al.* Epidermal expression of serine protease, neuropsin (KLK8) in normal and pathological skin samples. *Mol Pathol* **55**, 235-241 (2002).
25 Dear, T. N., Meier, N. T., Hunn, M. & Boehm, T. Gene structure, chromosomal localization, and expression pattern of Capn12, a new member of the calpain large subunit gene family. *Genomics* **68**, 152-160 (2000).
26 Spearman, R. I. The epidermal stratum corneum of the whale. *J Anat* **113**, 373-381 (1972).
27 Haldiman, J. T. *et al.* Epidermal and papillary dermal characteristics of the bowhead whale (Balaena mysticetus). *Anat Rec* **211**, 391-402 (1985).
28 Wang, S. K. *et al.* Novel KLK4 and MMP20 mutations discovered by whole-exome sequencing. *J Dent Res* **92**, 266-271 (2013).
29 Yamakoshi, Y. *et al.* Enamel proteins and proteases in Mmp20 and Klk4 null and double-null mice. *Eur J Oral Sci* **119 Suppl 1**, 206-216 (2011).
30 Meredith, R. W., Gatesy, J., Cheng, J. & Springer, M. S. Pseudogenization of the tooth gene enamelysin (MMP20) in the common ancestor of extant baleen whales. *Proc Biol Sci* **278**, 993-1002 (2011).
